# Supplementary material for: Cost-effectiveness of craniotomy versus decompressive craniectomy for UK patients with traumatic acute subdural haematoma
Source: BMJ Open. 2024 Jun 16;14(6):e085084. doi: 10.1136/bmjopen-2024-085084 (PMC11184173; doi:10.1136/bmjopen-2024-085084)
Supplement: Supplementary data [file bmjopen-2024-085084supp002.pdf]

BMJ Publishing Group Limited (BMJ) disclaims all liability and responsibility arising from any reliance placed on this supplemental material which has been supplied by the author(s)

SUPPLEMENTAL MATERIAL (TABLES)

Supplemental Table S1. Unit costs, for the 2018/19 financial year

Resource use	Unit cost (£)	Assumptions
Neurosurgical costs		
Index craniotomy or DC (hourly rate)	1,422[1]	Hourly rate applied to the duration of the operation, whether craniotomy or DC. Includes the time from entering pre-med until leaving theatre.
DC, not index procedure (hourly rate)	1,422[1]	Hourly rate applied to two-thirds of the mean length recorded for index DC. This accounts for the presence of previous skin incision and bone cuts.
Cranioplasty (operation cost, index or revision)	2,464[1,2]	Based on hourly rate above and 104 min duration, with an additional cost for both any synthetic material (if applicable, see below) and an additional associated NSU length of stay of 4 days if post-discharge (see below rates).
Haematoma evacuation (all types)	2,132[1]	Based on hourly rate above and 90 min duration (expert opinion)
Wound revision	2,132[1]	Based on hourly rate above and 90 min duration (expert opinion)
‘Other’ neurosurgical intervention	2,132[1]	Based on hourly rate above and 90 min duration (expert opinion)
Shunt placement (index or revision)	2,132[1]	Based on hourly rate above and 90 min duration (expert opinion) with an additional material cost (see below) and an associated NSU length of stay of 2 days if post-discharge
Synthetic material costs (design/parts) for cranioplasty	2,500	Estimated based on expert opinion (only added if the use of synthetic material was indicated on the relevant form). Not applicable for revisions.
Material costs for shunt	500	Estimated based on expert opinion. Not applicable for revisions.
Over-night stay costs		
Cost per bed day in Neuro-rehabilitation unit	504[3]	
Cost per bed day in NSU	365[4,5]	
Cost per bed day in ICU	1,691[6]	Assumes neurosciences adult patient in critical care, 2 or more organs supported (ICU)
Cost per bed day (other ward type)	354[4,5]	Weighted average of elective and non-elective excess bed days

Health professional visit costs	*Community*	*Hospital*	*Home*	*Assumptions*

Hospital doctor	33.00[4]	186.74[6]	59.40[4,7]	Community: as hospital doctors do not work in the community, the unit cost for a community GP visit was applied.<br>Home: as hospital doctors do not usually visit homes, the unit cost for a home GP visit was applied.
Nurse	12.31[4,7]	69.51[6]	19.64[4,7]	Home: costed as for community visit, plus 12 mins travel time
General Practitioner	33.00[4]	186.74[6]	59.40[4,7]	Hospital: as GPs do not work in hospitals, the unit cost for a hospital doctor visit was applied. Home: costed as for community visit, plus 12 mins travel time.
Physiotherapist	62.90[6]	54.96[6]	69.67[4,6,7]	Home: costed as for community visit, plus 12 mins travel time.
Occupational therapist	83.17[6]	65.54[6]	89.94[4,6,7]	Home: costed as for community visit, plus 12 mins travel time.
Speech therapist	106.51[6]	100.06[6]	113.28[4,6,7]	Home: costed as for community visit, plus 12 mins travel time
Social worker	118.81[4,8]	118.81[4,8]	127.72[4,7,8]	Home: costed as for community visit, plus 12 mins travel time.
Community care assistant	19.87[4,9]	19.87[4,9]	24.64[4,7,9]	Home: costed as for community visit, plus 12 mins travel time.
Emergency department	166.05[6]	166.05[6]	166.05[6]	Single rate costed for an emergency visit
Psychologist/neuropsychologist	141.17[4,10]	146.67[4,10]	156.57[4,7,10]	Home: costed as for community visit, plus 12 mins travel time.
Other	33.00[4]	186.74[6]	69.67[4,6,7]	The cost of the most commonly reported visits from each location are assigned.<br>Community: GP, Hospital: hospital doctor, home: physiotherapist
Other costs				*Assumptions*
MRI scan		120.83[6]		
CT scan		77.95[6]		
Unknown scan		77.95[6]		Assumed the cost of a CT scan
Care home (cost per week in residence)		1,854[11]		As no cost for adults with these specific needs has been estimated, we have used a cost for adults with autism and complex needs.
Carer time		17.27[12]		Gross hourly rate. Used to value carer time whether paid or not
Work time		17.27[12]		Gross hourly rate. Used to value lost work time, assigned to estimated time worked since their brain injury

DC, decompressive craniectomy; ICU, intensive care unit; NSU, neurosurgical care unit; MRI, magnetic resonance imaging; CT, computed tomography
Inflated to 2018/19 financial year prices, where necessary, using the NHSCII pay and prices.[4]

Supplemental Table S2. Proportion of Missing values (%) for key variables

Variable	Craniotomy	DC	Total
Baseline variables			
Treatment allocation	0	0	0
Age	0	0	0
Sex	0	0	0
EQ-5D-5L at baseline	39/126 (31.0%)	31/122 (25.4%)	70/248 (28.2%)
GCS score	6/126 (4.8%)	3/122 (2.5%)	9/248 (3.6%)
Cost variables			
Index admission costs (hospital-recorded data)*	17/126 (13.5%)	20/122 (16.4%)	37/248 (14.9%)
Cranioplasty and shunt costs (hospital-recorded data)†	2/126 (1.6%)	4/122 (3.3%)	6/248 (2.4%)
Post-discharge costs (patient self-report data)	27/126 (21.4%)	41/122 (33.6%)	68/248 (27.4%)
Outcome variables for health-related quality of life			
EQ-5D at 6 months	24/126 (19.1%)	28/122 (23.0%)	52/248 (21.0%)
EQ-5D at 12 months	15/126 (11.9%)	19/122 (15.6%)	34/248 (13.7%)
Outcome variables for GOSE			
GOSE at 6 months	13/126 (10.3%)	16/122 (13.1%)	29/248 (11.7%)
GOSE at 12 months	5/126 (4.0%)	7/122 (5.7%)	12/248 (4.8%)
Outcomes for cost-utility and cost-effectiveness analyses			
Total costs	40/126 (31.8%)	55/122 (45.1%)	95/248 (38.3%)
Total QALYS	58/126 (46.0%)	58/122 (47.5%)	116/248 (46.8%)
Binary GOSE at 12 months	5/126 (4.0%)	7/122 (5.7%)	12/248 (4.8%)
Binary GOSE dependent on GCS at 12 months	11/126 (8.7%)	10/122 (8.2%)	21/248 (8.5%)

*Includes index surgery, length of stay, neurosurgical interventions (excluding cranioplasties and shunts) during index admission.
†Includes cranioplasties and shunts (including revisions) during index admission and post-discharge.

References

1. Public Health Scotland. Scottish health service costs. Costs Book 19 (April 2018 to March 2019). See file R142X, available on the archive web-site, https://webarchive.nrscotland.gov.uk/20231203022410/https:/www.isdscotland.org/Health-Topics/Finance/Costs/File-Listings-2019.asp (2019, accessed 24 April 2024).

2. Fountain DM, Henry J, Honeyman S, et al. First Report of a Multicenter Prospective Registry of Cranioplasty in the United Kingdom and Ireland. *Neurosurgery* 2021; 89: 518–526.

3. Turner-Stokes L, Williams H, Bill A, et al. Cost-efficiency of specialist inpatient rehabilitation for working-aged adults with complex neurological disabilities: a multicentre cohort analysis of a national clinical data set. *BMJ Open* 2016; 6: e010238.

4. Curtis L, Burns A. *Unit costs of health and social care 2019*. Personal Social Services Research Unit, University of Kent, Canterbury, 2019. Epub ahead of print 2019. DOI: 10.22024/UniKent/01.02.79286.

5. Health and social Care Information Centre. *NHS Schedule of Reference Costs 2017/18*. London: Department of Health, 2018.

6. Health and Social Care Information Centre. *NHS Schedule of Reference Costs 2018/19*. London: Department of Health, 2019.

7. Curtis L, Burns A. *Unit costs of health and social care 2015*. Personal Social Services Research Unit, University of Kent, Canterbury, 2015.

8. Curtis L. *Unit costs of health and social care 2010*. Personal Social Services Research Unit, University of Kent, Canterbury, 2010.

9. Curtis L, Burns A. *Unit costs of health and social care 2017*. Personal Social Services Research Unit, University of Kent, Canterbury, 2017. Epub ahead of print 2017. DOI: 10.22024/UniKent/01.02/65559.

10. Curtis L, Burns A. *Unit costs of health and social care 2016*. Personal Social Services Research Unit, University of Kent, Canterbury, 2016.

11. Curtis L, Burns A. *Unit costs of health and social care 2018*. Personal Social Services Research Unit, University of Kent, Canterbury, 2018. Epub ahead of print 2018. DOI: 10.22024/UniKent/01.02.70995.

12. Office for National Statistics. *Annual Survey of Hours and Earnings (ASHE) Table 1 (revised)*, https://www.ons.gov.uk/employmentandlabourmarket/peopleinwork/earningsandworkinghours/datasets/allemployeesashetable1 (2019).
