# Supplementary material for: Cost-effectiveness of craniotomy versus decompressive craniectomy for UK patients with traumatic acute subdural haematoma
Source: BMJ Open. 2024 Jun 16;14(6):e085084. doi: 10.1136/bmjopen-2024-085084 (PMC11184173; doi:10.1136/bmjopen-2024-085084)
Supplement: Supplementary data [file bmjopen-2024-085084supp001.pdf]

SUPPLEMENTAL MATERIAL (APPENDICES)

APPENDIX 1:

AUTHOR AFFILIATIONS: Norwich Medical School, University of East Anglia, Norwich (Sarah C. Pyne, Garry R. Barton, David A Turner), the Division of Neurosurgery, Addenbrooke's Hospital (Angelos G. Kolias, Carole Turner, Hadie Adams, Midhun Mohan, Edoardo Viaroli, Adel E. Helmy, Ivan S. Timofeev, Peter J. Hutchinson), the Department of Clinical Neurosciences (Harry Mee, Angelos G. Kolias, Carole Turner, Hadie Adams, Midhun Mohan, Matthew G. Stovell, Edoardo Viaroli, Adel E. Helmy, Ivan S. Timofeev, Peter J. Hutchinson), and the Division of Anaesthesia (David K. Menon), University of Cambridge, and the Cambridge Clinical Trials Unit, Cambridge University Hospitals NHS Foundation Trust, Cambridge (Carole Turner), the Neurosurgery Department, Royal London Hospital (Christopher Uff, Shumaila Hasan), the Department of Neurosurgery, Imperial Neurotrauma Centre, Imperial College Academic Health Sciences Centre, St. Mary's Hospital (Mark H. Wilson), London, Wessex Neurological Centre, University Hospitals Southampton NHS Foundation Trust, Southampton (Diederik Bulters, Ardalan Zolnourian), the Department of Neurosurgery, Walton Centre NHS Foundation Trust, Liverpool (Catherine J. McMahon, Matthew G. Stovell), the Department of Neurosurgery and the Academic Directorate of Neurosciences, Sheffield Teaching Hospital NHS Foundation Trust, Sheffield (Yahia Z. Al-Tamimi), the Department of Neurosurgery, Leeds General Infirmary, Leeds (Simon Thomson), the Department of Neurosurgery, University Hospitals Birmingham NHS Foundation Trust, Birmingham (Antonio Belli), the Department of Neurosurgery, Manchester Centre for Clinical Neurosciences, Manchester (Andrew T. King), and the Neurosurgical Trials Group, Wolfson Research Centre, Newcastle University, Newcastle upon Tyne (Barbara Gregson) — all in the United Kingdom.

INVESTIGATORS AND TRIAL OVERSIGHT COMMITTEES

<u>RESCUE-ASDH trial collaborators (to be indexed on PubMed):</u>

- Harry Mee, Angelos G. Kolias, Carole Turner, Hadie Adams, Midhun Mohan, Edoardo Viaroli, Adel E. Helmy, Ivan S. Timofeev, David K. Menon, Peter J. Hutchinson – *University of Cambridge, UK*
- Peter J. Kirkpatrick, Hadie Adams, Nicola Owen, Kirsty Grieve, Alexis Joannides, Ellie Edlmann, Karen Caldwell, Silvia Tarantino, Francesca Hill, Liz Corteen, Janet Corn, Matthew G Stovell, Mathew Guilfoyle, Mohammad Naushahi, Richard Mair, Ibrahim Jalloh, Mark Kotter, Kamila Walker, Tamara Tajsic, Sara Venturini, Selma Tülü – *Cambridge University Hospital, Cambridge, UK*

- Christopher Uff, Shumaila Hasan, Chipo Chitsenega, Geetha Boyapati – *Royal London Hospital, London, UK*
- Muhammad Bhatti, Malik Zaben, Belinda Gunning, Natalia Ermalai, Dmitri Shastin, Mutwakil Abdulla, Liudmila, Joseph Merola – *University Hospital of Wales, Cardiff, UK*
- Stuart Smith, Laurence Glanz, Ashwin Kumaria, Lani Patterson – *Queen's Medical Centre, Nottingham, UK*
- Antonio Belli – *1. NIHR Surgical Reconstruction and Microbiology Research Centre, Birmingham, UK. 2. Institute of Inflammation and Ageing, University of Birmingham, Birmingham, UK*
- Colin Bergin, Maximina Ventura, Emma Toman – *NIHR Surgical Reconstruction and Microbiology Research Centre, Birmingham, UK*
- Giles Critchley, Laura Ortiz-Ruiz de Gordoa, Prasanna Epaliyanage, Husam Georges – *Royal Sussex County Hospital, Brighton, UK*
- Peter Whitfield, Sam Jeffrey, Charlotte Eglinton, Natasha Wilmhurst – *Derriford, Hospital, Plymouth, UK*
- Manjunath Prasad, Shahid Khan, Philip Kane, Emanuel Cirstea – *James Cook University Hospital, Middlesbrough, UK*
- Catherine McMahon, Geraint Sunderland, John Kitchen, Rushid Zakaria – *The Walton Centre, Liverpool, UK*
- Simon Thomson, Soumya Mukherjee, Ian Anderson, Linetty Makawa, Mary Kambafwile – *Leeds General Infirmary, Leeds, UK*
- Marios C. Papadopoulos, Mathew Joseph Gallagher, Siobhan Kearney, Sarah Trippier – *St. George's Hospital, London, UK*
- Mark Wilson, Hani Marcus, Rhys Thomas, Sonia Fernandez Lopez, Louise Young, Andrea D'Mello, Jo-Anna Conyngham – *St. Mary's Hospital, London, UK*
- Diederik Bulters, Ardalan Zolnourian, Patrick Holton, Miriam Taylor, Charlaine Reeve – *Southampton University Hospital, Southampton, UK*
- Jonathan Pollock, Ciaran Hill, Elisa Visentin, Laura Parker – *Queen's Hospital, Romford, UK*
- Kismet Hossain-Ibrahim, Paul Johnston – *Ninewells Hospital, Dundee, UK*
- Vasileios Arzoglou, Emma Clarkson, Arif Zafar, Efosa Ukponmwan – *Hull Royal Infirmary, Hull, UK*
- Christos Tolias, Joseph Frantzias, Anastasios Giamouriadis – *King's College London, London, UK*
- Yahia Al-Tamimi, Adam Wahba, Patrick Easton, Rose Clegg, Grace Cole – *Royal Hallamshire Hospital, Sheffield, UK*

- Damian Holliman, Louise Finlay – *Royal Victoria Infirmary, Newcastle, UK*
- Daniel Holsgrove, Alex Leggate, Jane Perez, Louise Harrison – *Salford Royal Hospital, Manchester, UK*
- Gareth Roberts, Terrie-Louise Cromie, Marianne Hare, Sonia Raj – *Royal Preston Hospital, Preston, UK*
- Tim Lawrence, Ruichong Ma, Javier Magan Ventura, Neil Davidson – *John Radcliffe Hospital, Oxford, UK*
- Richard Nelson, Ruth Womer, Beverley Fulkner – *Frenchay Hospital/North Bristol NHS Trust, Bristol, UK*
- Shabin Joshi, Geraldine Ward – *Walsgrave General Hospital, Coventry, UK*
- Ioannis Fouyas, Julie Woodfield, Himanshu Shekhar, Kareen Damley, Emma Fleming – *Western General Hospital, Edinburgh, UK*
- Roddy O'Kane, Michael Canty – *Queen Elizabeth University Hospital, Glasgow, UK*
- Indira Devi Bhagavatula, Dhananjaya Ishwar Bhat, Dhaval Prem Shukla, Kanti Konar, Nagesh Shanhag, Vaishali Nl Valluri – *National Institute of Mental Health and Neurosciences (NIMHANS), Bangalore, India*
- Manoj Kumar Tewari, Manjul Tripathi – *Post Graduate Institute of Medical Education and Research (PGIMER), Chandigarh, India*
- Deepak Gupta, Ashish Bindra, Kaveri Sharma – *All India Institute of Medical Sciences (AIIMS), Delhi, India*
- Mathew Joseph – *Christian Medical College, Vellore, India*
- Nicole Keong, Christine Lock, Chen Min Wei, Julian Han, Janell Kwok, Nicolas Kon Kam King – *National Neuroscience Institute, Singapore*
- Martina Stippler, Emmalin Nelton – *Beth Israel Medical Center, USA*
- Louis Anthony Whitworth, Sonja Stutzman, Christopher Madden, Caryn Harper, Tracey Moore, Vin Shen Ban, Alice Salazar – *The University of Texas Southwestern Medical Center, USA*
- Rocco Armonda, Jason J. Chang, George B. Moses, Patricia Tanjucto – *MedStar Washington Hospital Center, Washington, USA*
- Jamie Ullman, Orseola Arapi, Betsy Moclair – *Northwell Shore University Hospital (Northwell Health), New York, USA*
- Nrupen Baxi, Erin Lewis, Melvin Stone – *Jacobi Medical Center and Montefiore Medical Center, New York, USA*
- John Adair Prall, Meghan Baldwin, Jamie Jones – *Littleton Adventist Hospital (Centura Health), Colorado, USA*
- Clare Gallagher, Ish Bains – *Foothills Medical Centre, Calgary, Canada*

- Kesava Reddy, Paula Carroll – *Hamilton Health Sciences, Ontario, Canada*
- Leodante Da Costa, Nadia Scantlebury – *Sunnybrook Hospital, University of Toronto, Toronto, Canada*
- Fahad Alkherayf, Rafael Ochoa Sanchez, Ioana Moldovan – *The Ottawa Hospital, Ottawa, Canada*
- Sean Christie, Lisa Julien – *Queen Elizabet II Health Sciences Centre, Halifax, Canada*
- Kostas Fountas, Thanasis Paschalis – *University Hospital of Larissa, Medical School (University of Thessaly), Larissa, Greece*
- Sandro Krieg, Sebastian Ille – *Technische Universität München, Munich, Germany*
- Maria Luisa Gandia Gomez – *Hospital Universitario La Paz, Madrid, Spain*
- Alfonso Legares, Ana Maria Castaño Leon – *Hospital Universitario 12 de Octubre, Madrid, Spain*
- András Büki, Gábor Lenzsér – *University of Pécs, Pécs, Hungary*
- Tariq Khan, Mukhtar Khan – *Northwest General Hospital and Research Center, Peshawar, Pakistan*
- Franco Servadei, Massimo Tomei – *Humanitas Research Hospital, Milan, Italy*
- Vairavan Narayanan, Ronie Romelean Jayapalan – *University of Malaya Medical Centre, Kuala Lumpur, Malaysia*
- Martin Hunn, Panagiota Gkolia, Emily Galea – *The Alfred Hospital, Melbourne, Australia*
- Sarah C. Pyne, Garry R. Barton, David A Turner – *Health Economics Group, Norwich Medical School, University of East Anglia, UK*
- Barbara Gregson – *Neurosurgical Trials Group, Wolfson Research Centre, Newcastle University, Newcastle upon Tyne, UK*

Trial co-applicants and/or protocol contributors (to be indexed on PubMed):
Peter J. Hutchinson, Garry R. Barton, Clare Gallagher, Paula Kareclas, Angelos G. Kolias, Peter J. Kirkpatrick Sridevi Nagarajan, David Turner, James Piercy, Peter Kirkpatrick, A. David Mendelow, David K. Menon, Andrew King, Antonio Belli, John D. Pickard, Paul Brennan, Christopher Cowie

Cambridge Clinical Trials Unit staff (to be indexed on PubMed):
Carol Davis-Wilkie, Tapiwa Tungamirai, Kerstin Wolf, Kamila Walker, Natalia Igosheva, Alicia Gore, Paula Kareclas, Michele Jillings, Christopher Bushell

Trial Steering Committee members (to be indexed on PubMed):
Anthony Bell (chair), Allison Hirst, Peter McCabe, Simon Shaw Independent Data Monitoring and Ethics Committee members (to be indexed on PubMed):
Martin Smith (chair), Joan Grieve, Jonathan Cook

APPENDIX 2: BASELINE GCS-ADJUSTED GOSE (SLIDING-DICHOTOMY ANALYSIS)

Methods

In addition to the extended Glasgow Outcome Scale (GOSE) fixed dichotomy analysis described in the main paper, a further GOSE sliding dichotomy analysis was undertaken, in which the favourable/unfavourable categorisation was defined as follows: if GCS (Glasgow Coma Scale) at randomisation was between 3 and 8 (patient comatosed) (1), a favourable outcome was defined as upper severe disability or better but if GCS at randomisation was between 9 and 15 (responsive patient), a favourable outcome was defined as lower moderate disability or better. The cost-effectiveness analysis using this sliding dichotomy, replicated that described in the main paper for the fixed dichotomy, with a view to estimating the cost per additional favourable outcome.

Results and conclusions

For the craniotomy group compared with the DC group, the mean difference in cost was -£6,091 (95% CI -£18,857 to £6,675) with an odds ratio of favourable outcome on the GOSE score of 1.741 (95% CI 1.019 to 2.977). Craniotomy therefore dominated DC.

APPENDIX 3: FURTHER SENSITIVITY ANALYSES

Methods

In addition to the two sensitivity analyses described in the main paper, a further four were defined in the Health Economic Analysis Plan (HEAP) and analysed. The consequence of excluding patient self-reported resource use data (more missing data was expected from this source), and using only hospital-recorded costs was assessed as a sensitivity analysis (SA hospital-recorded post-discharge operations only). Another sensitivity analysis (SA patient-reported post-discharge operations only) included only patient-reported post-discharge skull/brain operations (with associated length of stay) instead of hospital-reported post-discharge cranioplasties and shunts. A further sensitivity analysis (SA per protocol) re-analysed the data on a per protocol basis, excluding patients whose primary treatment was not as allocated, e.g. allocated to DC but received craniotomy and vice versa. A complete case analysis based on the base-case was also undertaken (SA complete case analysis), where participants were only included if they have complete hospital records, participant self-report and QALY data, with no imputation undertaken. These sensitivity analyses were undertaken for both the cost-utility analysis and cost-effectiveness analysis.

Results and Conclusions

The results of the four sensitivity analyses described are presented in Table A1. In all sensitivity analyses, craniotomy was found to dominate DC. This is in keeping with the base-case cost-utility and cost-effectiveness analyses and other sensitivity analyses presented in this paper.

Supplemental Table A1 | Estimates of the mean incremental cost, incremental effect (QALY gain or odds ratio), and cost effectiveness of craniotomy compared with DC for additional sensitivity analyses.

Analysis (N craniotomy,N DC)	Incremental cost (95% CI)	QALY gain (95% CI)	ICER	CEAC*
SA hospital-recorded post-discharge operations only: (126,122) MI	-£6,252 (-£12,180 to -£325)	0.092 (0.031 to 0.153)	Dominant	99%
SA patient-reported post-discharge operations only: (126,122) MI	-£6,328 (-19,389 to £6,733)	0.093 (0.032 to 0.154)	Dominant	89%
SA per protocol: (113,114) MI	-£10,711 (-£23,361 to £1,939)	0.121 (0.056 to 0.185)	Dominant	98%
SA complete case analysis: (60,44)	-£1,917 (-£15,564 to £11,729)	0.071 (-0.0106 to 0.153)	Dominant	68%
**Analysis (N craniotomy,N DC)**	**Incremental cost (95% CI)**	**Odds ratio (95% CI) ‡**	**ICER**	
SA hospital-recorded post-discharge operations only: (126,122)	-£5,709 (-£11,783 to £365)	1.704 (1.010 to 2.888)	Dominant	-
SA patient-reported post-discharge operations only: (126,122)	-£5,374 (-£18,782 to £8,033)	1.687 (0.999 to 2.849	Dominant	-
SA per protocol: (113,114)	-£10,567 (-£23,434 to £2,299)	2.189 (1.252 to 3.827)	Dominant	-
SA complete case analysis: (83,67)	-£4,335 (-£18,545 to £9,876)	1.360 (0.698 to 2.649)	Dominant	-

DC= decompressive craniectomy; 95% CI=95% confidence interval; ICER =incremental cost-effectiveness ratio; Dominant = lower mean costs and higher mean effect; N craniotomy (N DC) = number Randomized to craniotomy/decompressive craniectomy who were included in the analysis; SA:sensitivity analysis, described in the Methods; QALY=Quality Adjusted Life Years; ICER =incremental cost-effectiveness ratio, described in the Methods; *Probability of being cost-effective on the CEAC at a threshold of £20,000 per QALY;‡ for a favourable outcome for craniotomy compared with DC, based on the GOSE (Extended Glasgow Outcome Scale), as described in the Methods.

APPENDIX 4: DEVIATION FROM THE HEAP IN "SA WIDER COST PERSPECTIVE"

Within the Health Economic Analysis Plan (HEAP) it was stated that lost productivity costs would be estimated. Below we explain why this was not undertaken.

The following was stated within the 'Costs' section for the HEAP:

"…Participants were asked to report a) whether they were currently working (paid or unpaid), with the following additional questions (if applicable); b) how many hours per week they work (paid or unpaid); c) whether the number of hours was the same as before their brain injury; d) whether they currently work fewer or more hours per week than before your brain injury; e) when they returned to work following the brain injury; f) whether they have taken any days off due to sickness since returning; g) if they have had to leave work / change job since their brain injury and why. In order to estimate lost productivity, in line with the opportunity cost method (2), the mean lost work time over the 12 month follow-up period (regardless of whether a payment was made) will be estimated and valued at the 2019 UK mean hourly gross wage (£17.25) (3)…"

Within the 'Analysis' section for the HEAP we stated that the base-case analysis would be from the cost perspective of the NHS and PSS. However, it was stated that the first sensitivity analysis (SA) ("SA wider cost perspective" in this paper) would take a more societal perspective and include lost productivity costs, as well as care home and carer costs.

We attempted to include lost productivity costs at the analysis stage but found that we did not have information as to the number of hours participants were working before their brain injury, as intended. The main reason for this was that if a participant reported that they were *not* currently working in response to the above question a) they were not asked to complete questions b-f. In hindsight, this was an error in how the questionnaire was formulated, and they should have been asked to complete questions c and d as well. Considering this error in the framing of the questionnaire we chose to deviate from the HEAP and not estimate lost productivity costs. Consequently, as detailed in the paper, in "SA wider cost perspective" only the care home and carer costs were added to the (base-case) NHS and PSS costs.

References

1. Bullock MR, Chesnut R, Ghajar J, et al.: Surgical management of acute subdural hematomas. *Neurosurgery* 2006; 58:S2-16-S2-24
2. Hoefman RJ, Van Exel J, Brouwer W: How to include informal care in economic evaluations. *Pharmacoeconomics* 2013; 31:1105–1119
3. Office for National Statistics: Annual Survey of Hours and Earnings (ASHE) Table 1 (revised) [Internet]. 2019. Available from: https://www.ons.gov.uk/employmentandlabourmarket/peopleinwork/earningsandworkinghours/datasets/allemployeesashetable1
