# Supplementary material for: Cost-effectiveness of craniotomy versus decompressive craniectomy for UK patients with traumatic acute subdural haematoma
Source: BMJ Open. 2024 Jun 16;14(6):e085084. doi: 10.1136/bmjopen-2024-085084 (PMC11184173; doi:10.1136/bmjopen-2024-085084)
Supplement: Author's manuscript [file bmjopen-2024-085084.draft_revisions.pdf]

BMJ Open

BMJ Open is committed to open peer review. As part of this commitment we make the peer review history of every article we publish publicly available.

When an article is published we post the peer reviewers' comments and the authors' responses online. We also post the versions of the paper that were used during peer review. These are the versions that the peer review comments apply to.

The versions of the paper that follow are the versions that were submitted during the peer review process. They are not the versions of record or the final published versions. They should not be cited or distributed as the published version of this manuscript.

BMJ Open is an open access journal and the full, final, typeset and author-corrected version of record of the manuscript is available on our site with no access controls, subscription charges or pay-per-view fees (http://bmjopen.bmj.com).

If you have any questions on BMJ Open's open peer review process please email info.bmjopen@bmj.com

BMJ Open

Cost-effectiveness of craniotomy versus decompressive craniectomy, for UK patients with traumatic acute subdural hematoma

Journal:	*BMJ Open*
Manuscript ID	bmjopen-2024-085084
Article Type:	Original research
Date Submitted by the Author:	06-Feb-2024
Complete List of Authors:	Pyne, Sarah; University of East Anglia, Norwich Medical School<br>Barton, Garry ; University of East Anglia Faculty of Medicine and Health Sciences, Norwich Medical School<br>Turner, David; University of East Anglia, Norwich Medical School<br>Mee, Harry; University of Cambridge, Department of Clinical Neurosciences<br>Gregson, Barbara; Newcastle University, Neurosurgical Trials Group, Wolfson Research Centre<br>Kolias, Angelos; Cambridge University, Division of Neurosurgery<br>Turner, Carole; University of Cambridge, Department of Clinical Neurosciences; Addenbrooke's Hospital, Division of Neurosurgery<br>Adams, Hadie; University of Cambridge, Department of Clinical Neurosciences; Addenbrooke's Hospital, Division of Neurosurgery<br>Mohan, Midhun; University of Cambridge, Department of Clinical Neurosciences; Addenbrooke's Hospital, Division of Neurosurgery<br>Uff, Christopher; The Royal London Hospital<br>Hasan, Shumaila; The Royal London Hospital<br>Wilson, Mark; St Mary's Hospital, Department of Neurosurgery<br>Bulters, Diederik ; University Hospital Southampton NHS Foundation Trust, Wessex Neurological Centre<br>Zolnourian, Ardalan; University Hospital Southampton NHS Foundation Trust<br>McMahon, Catherine; The Walton Centre NHS Foundation Trust, Department of Neurosurgery<br>Stovell, Matthew G; University of Cambridge, Department of Clinical Neurosciences<br>Al-Tamimi, YAHIA; Sheffield Teaching Hospitals NHS Foundation Trust, Department of Neurosurgery; Sheffield Teaching Hospitals NHS Foundation Trust, Academic Directorate of Neurosciences<br>Thomson, Simon; Leeds General Infirmary, Department of Neurosurgery<br>Viaroli, Edoardo; Addenbrooke's Hospital, Division of Neurosurgery; University of Cambridge, Department of Clinical Neurosciences<br>Belli, Antonio; University Hospitals Birmingham NHS Foundation Trust, Department of Neurosurgery<br>King, Andrew; Salford Royal Hospital Manchester Centre for Clinical Neurosciences, Department of Neurosurgery<br>Helmy, Adel E; Addenbrooke's Hospital, Division of Neurosurgery; University of Cambridge, Department of Clinical Neurosciences<br>Timofeev, Ivan; University of Cambridge, Department of Clinical

	Neurosciences; Addenbrooke's Hospital, Division of Neurosurgery<br>Menon, David; University of Cambridge, Division of Anaesthesia<br>Hutchinson, Peter; University of Cambridge, Department of Clinical Neurosciences; Addenbrooke's Hospital, Division of Neurosurgery<br>RESCUE-ASDH, trial collaborators; University of Cambridge

Keywords:	HEALTH ECONOMICS, Health economics < HEALTH SERVICES ADMINISTRATION & MANAGEMENT, NEUROSURGERY, Randomized Controlled Trial, Neurosurgery < SURGERY, Brain Injuries

SCHOLARONE™
Manuscripts

I, the Submitting Author has the right to grant and does grant on behalf of all authors of the Work (as defined in the below author licence), an exclusive licence and/or a non-exclusive licence for contributions from authors who are: i) UK Crown employees; ii) where BMJ has agreed a CC-BY licence shall apply, and/or iii) in accordance with the terms applicable for US Federal Government officers or employees acting as part of their official duties; on a worldwide, perpetual, irrevocable, royalty-free basis to BMJ Publishing Group Ltd ("BMJ") its licensees and where the relevant Journal is co-owned by BMJ to the co-owners of the Journal, to publish the Work in this journal and any other BMJ products and to exploit all rights, as set out in our licence.

The Submitting Author accepts and understands that any supply made under these terms is made by BMJ to the Submitting Author unless you are acting as an employee on behalf of your employer or a postgraduate student of an affiliated institution which is paying any applicable article publishing charge ("APC") for Open Access articles. Where the Submitting Author wishes to make the Work available on an Open Access basis (and intends to pay the relevant APC), the terms of reuse of such Open Access shall be governed by a Creative Commons licence – details of these licences and which Creative Commons licence will apply to this Work are set out in our licence referred to above.

Other than as permitted in any relevant BMJ Author's Self Archiving Policies, I confirm this Work has not been accepted for publication elsewhere, is not being considered for publication elsewhere and does not duplicate material already published. I confirm all authors consent to publication of this Work and authorise the granting of this licence.

For peer review only

Cost-effectiveness of craniotomy versus decompressive craniectomy, for patients with traumatic acute subdural hematoma

Authors:

Sarah C Pyne, Garry R Barton*, David A Turner, Harry Mee, Barbara Gregson, Angelos G Kolias, Carole Turner, Hadie Adams, Midhun Mohan, Christopher Uff, Shumaila Hasan, Mark H Wilson, Diederik Bulters, Ardalan Zolnourian, Catherine J McMahon, Matthew G Stovell, Yahia Z Al-Tamimi, Simon Thomson, Edoardo Viaroli, Antonio Belli, Andrew T King, Adel E Helmy, Ivan S Timofeev, David K Menon, Peter J Hutchinson, RESCUE-ASDH trial collaborators †

*Corresponding author: Professor Garry Barton, Health Economics Group, Norwich Medical School, University of East Anglia, Norwich NR4 7TJ. Email: g.barton@uea.ac.uk

†Author affiliations, along with a complete list of the investigators and members of the trial oversight committees is provided in the Supplemental Material (Appendix 1).

Competing interests: No support from any organisation other than the National Institute for Health and Care Research was received for the submitted work. Barbara Gregson has received consulting fees from Cambridge University Hospitals NHS Foundation Trust. Angelos Kolias is supported by a Senior Lectureship at the School of Clinical Medicine, University of Cambridge, the Wellcome Trust, and the Royal College of Surgeons of England. Mark Wilson has received support for attending meetings and/or travel for presentations with the Wilderness Medical Society and Royal College of Surgeons of Edinburgh, is a member of the Trauma Clinical Reference group for the NHS, meetings secretary for the Society of British Neurosurgeons and a non-salaried medical director of GoodSAM. Peter Hutchinson is supported by a Research professorship and Senior Investigator award from the NIHR, the NIHR Cambridge Biomedical Research Centre, and the Royal College of Surgeons of England.

ABSTRACT

Objective: To estimate the cost-effectiveness of craniotomy, compared with decompressive craniectomy (DC) in UK patients undergoing evacuation of acute subdural haematoma (ASDH).

Design: Economic evaluation undertaken using health resource use and outcome data from the 12-month multi-centre, pragmatic, parallel-group, randomised, RESCUE-ASDH trial.

Setting: UK secondary care.

Participants: 248 UK patients undergoing surgery for traumatic ASDH randomised to craniotomy (n=126) or DC (n=122).

Interventions: Surgical evacuation via craniotomy (bone flap replaced) or decompressive craniectomy (bone flap left out with a view to replace later cranioplasty surgery).

Main outcome measures: In the base-case analysis costs were estimated from an NHS and personal social services perspective. Outcomes were assessed via the Quality-Adjusted Life Years (QALY) derived from the EQ-5D-5L questionnaire (cost-utility analysis) and the Extended Glasgow Outcome Scale (GOSE) (cost-effectiveness analysis). Multiple imputation and regression analyses were conducted to estimate the mean incremental cost and effect of craniotomy compared to DC. The most cost-effective option was selected, irrespective of the associated level of uncertainty as is argued by economists.

Results: In the cost-utility analysis the mean incremental cost of craniotomy compared to DC was estimated to be -£5,520 (95% confidence interval (CI) -£18,060 to £7,020) with a mean QALY gain of 0.093 (95% CI 0.029 to 0.156). In the cost-effectiveness analysis, the mean incremental cost was estimated to be -£4,536 (95% CI -£17,374 to £8,301) with an odds ratio of 1.682 (95% CI 0.995 to 2.842) for a favourable outcome on the GOSE.

Conclusions: In a UK population with traumatic ASDH craniotomy was estimated to be cost-effective compared to decompressive craniectomy: craniotomy was estimated to have a lower mean cost, higher mean QALY gain and higher probability of a more favourable outcome on the GOSE (though the estimated differences between the two approaches were not statistically significant).

For peer review only - http://bmjopen.bmj.com/site/about/guidelines.xhtml

Trial registration and ethics: Ethical approval for the trial was obtained from the North West – Haydock Research Ethics Committee in the United Kingdom on 17th July 2014 (14/NW/1076). The trial was registered prospectively: ISRCTN87370545.

STRENGTHS AND LIMITATIONS OF THIS STUDY

- This study is based on individual patient-level data from a large, pragmatic, multi-centre randomised trial.
- It is both the first randomised trial and the first economic evaluation to compare craniotomy to decompressive craniectomy.
- Multiple imputation was undertaken to account for missing data.
- For ethical reasons, baseline EQ-5D-5L scores were taken at discharge from neurosurgical unit (NSU), rather than at randomisation.
- A number of sensitivity analyses were undertaken to assess the robustness of conclusions to different assumptions in relation these and other aspects.

BACKGROUND

In the UK an estimated 1.3 million people live with a traumatic brain injury related disability and the annual societal cost has been estimated to be £15 billion (2015 cost levels).[1] Acute subdural hematoma (ASDH) is a common consequence[2] where craniotomy and decompressive craniectomy (DC) are the two mainstay treatments for surgical evacuation of the hematoma.[3] Both involve the removal of a piece of skull (bone-flap) to evacuate the hematoma. With craniotomy the bone-flap is replaced, whereas with DC it is not. DC may help alleviate brain swelling and is undertaken with the view to a further operation being performed to rebuild the skull (cranioplasty). A systematic review found few studies comparing the two procedures, none of which were randomised, with contrasting evidence as to which was superior.[3]

Given this uncertainty as to whether craniotomy or DC is the more effective treatment for patients with ASDH, the choice of treatment is generally left to the discretion of the surgeon.[3] However, guidance/recommendations for the provision of different treatment options are now often based on estimated levels of cost-effectiveness.[4] Moreover, levels of cost-effectiveness may differ between these two surgical procedures as, for example, DC often requires cranial reconstruction by means of cranioplasty, which has additional costs and a significant complication profile,[5] but may better alleviate brain swelling, translating into

For peer review only - http://bmjopen.bmj.com/site/about/guidelines.xhtml

quality-of-life benefits.[5] Thus, here we report an economic evaluation[6] that was conducted alongside the RESCUE-ASDH (Randomized Evaluation of Surgery with Craniectomy for Patients Undergoing Evacuation of Acute Subdural Hematoma) trial,[5] to compare the cost-effectiveness of craniotomy versus DC for UK patients with traumatic ASDH.

METHODS

Participants

The RESCUE-ASDH trial[5] is a multicentre, international, pragmatic, parallel-group, randomised trial that compared craniotomy with DC. Patients were eligible if they were ≥16 years, had an ASDH on CT scan, and the admitting surgeon felt that the hematoma needed evacuating either by craniotomy or DC. The economic evaluation was nested within the RESCUE-ASDH trial and based on UK participants only.

Treatment and randomisation

Enrolled patients had their ASDH evacuated in the operating room under general anaesthesia. The bone-flap was raised, the dura opened, and the hematoma evacuated, after which patients were randomly assigned to receive either craniotomy (bone-flap restored before skin closure) or DC (bone-flap removed prior to skin closure with a view to being restored later). Patients were only randomised if either treatment was feasible, those patients whose brain was too swollen to allow replacement of the bone flap were not randomised. These patients would have the bone flap left out and were not included in the ITT analysis presented within this paper. As a pragmatic study, management of patients pre-, intra-, and post-operatively was undertaken according to each centre's standard of care.

Blocked randomisation (block size 4) with 1:1 ratio was used, with allocation stratified by geographical region, age group, severity of injury and CT findings.[5] Patients randomised to craniotomy could have a DC at a later stage if their condition deteriorated and at the discretion of the treating clinician. It was not possible to blind patients, relatives and treating clinicians but the primary outcome (see below) was adjudicated centrally by blinded investigators.

Measuring costs

Costs were estimated from a UK NHS and Personal Social Services (PSS) perspective.[7] Resource use data was collected via two methods: hospital-recorded data and a patient self-report (12-month follow-up) questionnaire (PSRQ). Both methods of data collection were developed in consultation with hospital staff/patients and focussed on big cost drivers/resources that were expected to differ between arms.[8] All resource use items that were costed (see below) were estimated in £ Sterling for the 2018/19 financial year (see

For peer review only - http://bmjopen.bmj.com/site/about/guidelines.xhtml

Supplemental Table S1 for unit costs), resource items undertaken for research purposes were not costed.

The hospital-recorded data included: details of the intervention (craniotomy or DC) including length of operation and graft details; time spent in the intensive care unit (ICU) and neurosurgical unit (NSU) during initial (index) admission; cranioplasties and shunt placements received during index admission and after discharge from the NSU; any further neurosurgical procedures received during index admission.

The 12-month follow-up PSRQ could be completed by a relative/friend/carer if the participant was unable to complete it and referred only to the time since discharge from NSU. Information requested included: overnight stays in a hospital or other healthcare facility (length of stay, ward type, any associated skull/brain operation); healthcare professional visits (professional seen, frequency and most common location); head/brain scans (MRI, CT or 'other'); time in a care home; help received from a family member/friend or carer.

Component costs, excluding wider societal costs (care home and help/carer costs), were summed to estimate the total NHS and PSS cost per participant. For each group the mean total and component costs were estimated over the 12-month follow-up period, along with the associated p-value for the mean cost difference between groups. An exception to the above was that, to avoid double-counting, patient self-reported post-discharge overnight stays with an associated skull/brain operation would not be costed if the total reported number was less than the total reported number of hospital-recorded post-discharge cranioplasty and shunt procedures (including revisions). In line with previous work,[9] the higher of the two values was considered the most accurate.

Measuring outcomes

To estimate health-related quality-of-life, and conduct a cost-utility analysis,[6] in line with UK NICE guidance,[4,10] the five-dimension EuroQoL five-level questionnaire (EQ-5D-5L)[11] was combined with mortality data to estimate quality-adjusted life-year (QALY)[6] scores. Participants completed the EQ-5D-5L at discharge from NSU (assumed baseline score), 6- and 12-months follow-up (if discharged from NSU by these time points). As recommended at the time of analysis,[12] the crosswalk mapping function[13] was used to convert responses into utility scores (range: -0.594 (worse than death) to 1 (full health)). Participants who died were assigned a utility score of 0 on their date of death. Utility values were used to estimate QALYs over 12 months, based on the total area under the curve method and linear interpolation.[14]

For peer review only - http://bmjopen.bmj.com/site/about/guidelines.xhtml

The trial primary outcome measure, the extended Glasgow Outcome Scale (GOSE),[15–18] at 12 months was collapsed into a fixed dichotomy analysis (i.e. favourable vs. unfavourable)[5] and used to undertake a cost-effectiveness analysis.[6] Favourable outcomes were defined as upper severe disability or better; while unfavourable outcomes included death, vegetative state and lower severe disability. A sliding dichotomy analysis[5] was also undertaken and is described in the Supplemental Material (Appendix 2).

Missing data

Missing data is common in randomised trials and can lead to bias and lack of precision.[19] As recommended, patterns of missing data were examined to explore the mechanism of missingness.[19] Accordingly, multiple imputation (MI) with chained equations (MICE) under MAR (missing at random) was used to impute missing data, by treatment group. The "mi impute chained" command (Stata 17.0 [StataCorp LP, College Station, TX]) was used to create 30 data sets (in line with the level of missing data[19] that were then pooled using Rubin's rules.[20]

In addition to the costs (index admission, cranioplasty and shunt, and post-discharge), EQ-5D-5L scores (baseline, 6 and 12 months), Glasgow Coma Scale (GCS) score[21,22] (baseline) and GOSE scores (12 months), the MI model included age (years), sex and time post-discharge (the number of days from discharge to the 12-month point or death).

Incremental analyses

For both the cost-utility and cost-effectiveness analyses, a 12-month within-trial, intention-to-treat (ITT) approach was adopted. In this base-case analysis, patients were analysed according to the treatment to which they were randomised, regardless of treatment received. No discounting was undertaken.

For the cost-utility analysis, to estimate the mean incremental cost and incremental effect (QALY gain) associated with craniotomy compared with DC, seemingly unrelated regression analysis was undertaken.[23] Regressions included those baseline variables expected to be predictive of total costs and outcomes: age (years), sex and baseline utility score. Assuming dominance,[6] where an intervention was both more costly and less effective, did not occur the incremental cost-effectiveness ratio (ICER = mean incremental cost/QALY),[10] for craniotomy versus DC, would be estimated.[6] In the UK, NICE refers to a cost-effectiveness threshold of £20,000 to £30,000 per QALY.[10] As such, if craniotomy had an ICER below this level, this would suggest it is cost-effective, compared with DC. It should be noted that economists have argued that decisions about treatment adoption should be made based on mean estimates, irrespective of whether such differences are statistically significant.[24] Therefore, the treatment option which is estimated to be most cost-effective should be provided,

For peer review only - http://bmjopen.bmj.com/site/about/guidelines.xhtml

regardless of the associated level of uncertainty.[25] This approach is consistent with the objective of maximizing benefits for a given expenditure.

The cost-effectiveness analysis mirrored that of the cost-utility analysis, but (in the absence of dominance) would estimate a cost per additional favourable outcome, as measured by the GOSE. As the outcome is binary (favourable/unfavourable), logistic (logit) regression was undertaken to estimate the odds ratio (95% CI) of a favourable outcome for craniotomy compared with DC. Mean incremental costs associated with craniotomy compared with DC were estimated using linear regression. Both regressions included variables age (years) and sex, which were expected to be predictive of total costs and GOSE outcomes.

Decision uncertainty

To estimate the level of uncertainty associated with the decision regarding cost-effectiveness, Fieller's theorem[19] was used to calculate the probability of craniotomy being cost-effective, compared with DC, at the threshold of £20,000/QALY on the cost-effectiveness acceptability curve (CEAC).[25]

Sensitivity analyses

The above analysis constituted the base-case analysis[6] and was carried out in accordance with a pre-specified health economic analysis plan (HEAP) (see: https://www.rescueasdh.org/trial-documents). To assess the robustness of conclusions, sensitivity analyses (SA) were undertaken.[6] To analyse the data from a wider cost perspective the care home and carer costs (which were excluded from the base-case analysis) were added to the total NHS and PSS costs (SA wider cost perspective). A further sensitivity analysis (for the cost-utility analysis only) tested the use of the EQ-5D-5L score at discharge from NSU, as the baseline for QALY calculations. As any benefits could already have been partially/wholly achieved by discharge, QALY scores were re-estimated with the assumption that, given the grave nature of the condition and following expert advice, participants had the lowest possible EQ-5D-5L score at baseline (date of index surgery): -0.594 (SA lowest EQ-5D baseline score). Four further sensitivity analyses (including a per protocol analysis) were conducted and are presented in the Supplemental Material (Appendix 3). "SA wider cost perspective" deviated from the HEAP, for reasons explained in the Supplemental Material (Appendix 4)

Patient and public involvement

The aforementioned patient self-report questionnaire was developed in consultation with non-trial patients.

For peer review only - http://bmjopen.bmj.com/site/about/guidelines.xhtml

RESULTS

Participants

Between September 2014 and April 2019 248 UK patients were recruited, 126 in the craniotomy arm and 122 in the DC arm. Compared with the full analysis population (452 patients),[5] these UK patients are slightly older (3.5 years on average) and more likely to be on antithrombotic medication (Table 1).

Levels of missing data were slightly lower in the craniotomy group compared with the DC group for cost variables and outcome variables (except at baseline) (Supplemental Table S2).

Costs

Levels of resource use by intervention arm are summarised in Table 2, under three main categories: (i) hospital-recorded index-admission; (ii) Hospital-recorded 12-month follow-up, cranioplasties and shunts; (iii) Patient-reported (PSRQ) post-discharge.

The hospital-recorded index-admission data shows that, length of stay in ICU and NSU was slightly lower in the craniotomy group compared with the DC group, but not significantly so. Only small numbers of other neurosurgical operations were reported. The hospital-recorded 12-month follow-up data shows that, as expected, more patients in the DC group had cranioplasties than in the craniotomy group (DC is pre-requisite to a cranioplasty). There were, however, 30/126 patients who were randomised to craniotomy that went on to have a DC, 21 of which went on to have a cranioplasty in the 12-month follow-up period. Most cranioplasties used a synthetic material. Shunts were uncommon and occurred at a similar frequency between the groups. In terms of the patient-reported (PSRQ) post-discharge resource use, there were no significant differences between the groups for any of the parameters measured.

Mean cost estimates are summarised in Table 3 and divided into the same three main categories. As expected, given the procedure complexity and recovery time, total NHS and PSS costs are high in both groups. High index admission costs particularly accounted for this, largely due to the high cost of ICU stays, along with post-discharge costs, largely due to the high cost of overnight stays on a rehabilitation unit. There were however few significant differences between groups, the only notable one being the cost of cranioplasty procedures which, for aforementioned reasons, was significantly higher in the DC group. As the number of post-discharge hospital-recorded cranioplasty/shunt procedures exceeded patient-reported over-night stays with an associated skull/brain operation (Table 2), the latter has not been costed.

For peer review only

Outcomes

Outcomes are summarised in Table 4. Follow-up mean EQ-5D-5L scores were higher in the craniotomy group compared with the DC group, significantly so at 12 months. Furthermore, the change in EQ-5D-5L score from baseline was significantly higher at both 6 and 12 months in the craniotomy group compared with the DC group. There was no significant difference between groups for the total QALY score, based on available data.

At 12 months the percentage of favourable GOSE scores was higher, but not significantly, in the craniotomy group compared with the DC group.

Analyses

Cost-utility analysis

For the base-case (based on ITT/MI), the mean difference in cost for the craniotomy group compared with the DC group was -£5,520 (95% CI -£18,060 to £7,020) with a mean QALY difference of 0.093 (95% CI 0.029 to 0.156) (Table 5). Craniotomy therefore dominated DC; it was estimated to be associated with both lower costs and more benefit.

Cost-effectiveness analysis

For the craniotomy group compared with the DC group, the mean difference in cost was -£4,536 (95% CI -£17,374 to £8,301) with an odds ratio of favourable outcome on the GOSE score of 1.682 (95% CI 0.995 to 2.842) (Table 5). Again, craniotomy therefore dominated DC.

Decision uncertainty

The base-case probability that craniotomy was cost-effective compared with DC, at a threshold of £20,000/QALY, was 87% (Table 5). This indicates a high degree of certainty associated with the cost-utility analysis decision that craniotomy compared with DC is cost-effective at that threshold.

Sensitivity analyses

In the sensitivity analyses, from a wider cost perspective and using the lowest EQ-5D baseline score (for the cost-utility analysis only), craniotomy was again found to dominate DC (see Table 5). Results of further sensitivity analyses, all of which are consistent with the base-case results, are presented in Supplemental Table A1 (Supplemental Material, Appendix 3).

For peer review only

DISCUSSION

Main findings

In this UK population of patients with traumatic ASDH that warrants surgical evacuation, based on the results of the cost-utility and cost-effectiveness analyses, craniotomy dominated DC as it was estimated to have a lower mean cost, a higher mean QALY gain / higher probability of a more favourable outcome on the GOSE (though none of these estimated differences were statistically significant). (Table 5). Craniotomy was therefore estimated to be cost-effective, on the basis that the associated level of significance is considered to be irrespective.[24,25] In the cost-utility analysis (QALY outcome), there was only an estimated 13% probability (at a threshold of £20,000/QALY) of making the wrong decision by choosing craniotomy. The results of the sensitivity analyses are in keeping with this result.

Within this study it is important to highlight that costs were estimated from the viewpoint of the UK NHS and PSS and that associated resource use and outcome data was based only on participants from UK sites. As e.g. unit costs may differ outside the UK it is important to note that it is unclear whether these results are generalisable to sites outside the UK. Further associated research may therefore be warranted in relation to this and that approximately 25% of patients who were randomised to craniotomy went on to have a DC (as an ITT approach was adopted these patients were included in the craniotomy arm in the base-case analysis).

Strengths and limitations

In line with good practice recommendations for cost-effectiveness analyses,[8] we concentrated on the large cost drivers and excluded resources that were not expected to differ between treatment arms (e.g. routine monitoring scans/tests). That said, the mean resource use levels could be heavily influenced by outliers, due to the high unit costs associated with items such as length of stay in ICU and operation costs.

Regarding health-related quality-of-life, QALY scores (EQ-5D-5L recorded at all time points) were available for 53% of participants only and the amount of missing data was greater at discharge than at 6 and 12 months (Supplemental Table S2). Some missing EQ-5D-5L baseline (NSU discharge) data may be due to participants being discharged at short notice or at the weekend when a research nurse was not available. As some patients had not yet been discharged from hospital by 6 months, this may explain the higher rates of missing data at this time point compared with 12 months. Post-discharge costs (based on patient self-report data) were also missing for 27.4% of patients at 12 months (Supplemental Table

For peer review only - http://bmjopen.bmj.com/site/about/guidelines.xhtml

S2). Such missing data is a limitation, but we did impute missing data and take an ITT approach, which meant that all patients were still included in the analysis.

A further limitation is that, for ethical reasons, baseline EQ-5D-5L scores were taken at discharge from NSU, rather than at randomisation. Therefore, any benefits could be underestimated by assuming this score is the baseline score. To test the potential impact of this, a sensitivity analysis (SA lowest EQ-5D baseline score) assumed the baseline EQ-5D-5L score to be that of worst possible health state (-0.594). The results differ little from the base-case (Table 5) with craniotomy still dominating DC.

The main strength of this economic evaluation is that it is based on a large, multi-centre, randomised trial. Previously there were no randomised trials investigating this topic and the existing evidence was inconclusive and highlighted the uncertainty in how to treat patients with ASDH.[3,26,27]

Comparisons with other studies

We are not aware of any previous economic evaluations that have specifically compared craniotomy with DC for patients with ASDH. Previous economic evaluations of DC have been undertaken,[28–32] but these have had different comparators, and used a variety of different populations/methods (most developed a decision analytical model to estimate costs and benefits,[30–32] and the two papers[28,29] that used actual patient data were not based on randomised data and were of a smaller sample size than used here), with different cost perspectives and timeframes. Thus, it is difficult to make direct comparisons to our study, and the use of different methods may explain why there were differences in the results as to whether DC was estimated to be cost-effective or not.[28–32]

Implications

In a UK population of patients with traumatic ASDH, craniotomy was estimated to have a lower mean cost, a higher mean QALY gain and a higher probability of a more favourable outcome on the GOSE, dominating DC. Based on the QALY, there was a high probability that craniotomy, compared with DC, was cost-effective (at a threshold of £20,000/QALY). When sensitivity analyses were conducted, the main conclusion (that craniotomy was therefore estimated to be cost-effective) remained unchanged. Consequently, the health economic analysis supports the recommendation, based on the primary outcome,[5] that a craniotomy should be undertaken, rather than a DC, if it is operatively feasible to replace the bone-flap.

For peer review only - http://bmjopen.bmj.com/site/about/guidelines.xhtml

Funding statement: This project was supported by the Health Technology Assessment (HTA) Programme (project number 12/35/57) and will be published in full in the HTA journal at https://fundingawards.nihr.ac.uk/award/12/35/57; The RESCUE-ASDH trial is an "embedded study" linked with the CENTER-TBI project (https://www.center-tbi.eu/) of the European Brain Injury Consortium. CENTER-TBI was a large-scale collaborative project, supported by the FP7 Program of the European Union (grant number 602150); RESCUE-ASDH ISRCTN Registry number, ISRCTN87370545.

Study protocol is available at https://fundingawards.nihr.ac.uk/award/12/35/57

The views expressed are those of the authors and not necessarily those of the NHS, the National Institute for Health and Care Research (NIHR), or the Department of Health and Social Care.

We thank the patients who participated in the RESCUE-ASDH trial, their families, and all the collaborating clinicians and research staff, and we thank the staff of the Cambridge Clinical Trials Unit for their support.

For peer review only

Contributors:

GRB, DAT, HM, BG, AGK, CT, HA, MM, CJM, AB, ATK, DKM and PJH contributed to the conception/design of the work. SCP, GRB, DAT, HM, BG, AGK, CT, HA, MM, CU, SH, MW, DB, AZ, CJM, MGS, YZA, ST, EV, AEH, IST, DKM, PJH contributed to the acquisition of data. SCP, GRB and DT conducted the analysis. All authors contributed to the interpretation of data/drafting of the paper (led by SCP and GRB) and approved the final manuscript.

Data availability statement: Reasonable requests to make relevant anonymised participant level data available will be considered by the trial team.

REFERENCES

1. Parsonage M. *Traumatic brain injury and offending. An economic analysis*. 2016.

2. Wilberger JE, Harris M, Diamond DL. Acute subdural hematoma: morbidity, mortality, and operative timing. *J Neurosurg* 1991; 74: 212–8.

3. Phan K, Moore JM, Griessenauer C, et al. Craniotomy versus decompressive craniectomy for acute subdural hematoma: systematic review and meta-analysis. *World Neurosurg* 2017; 101: 677-685.e2.

4. NICE. *NICE health technology evaluations: the manual (PMG 36)*. 2022.

5. Hutchinson PJ, Adams H, Mohan M, et al. Decompressive Craniectomy versus Craniotomy for Acute Subdural Hematoma. *NEJM* 2023; 388: 2219–2229.

6. Drummond M, Sculpher M, Claxton K, et al. *Methods for the economic evaluation of health care programmes*. 4th ed. Oxford, UK: Oxford University Press, 2015.

7. NICE. *Guide to the methods of technology appraisal 2013*. 2013. Epub ahead of print 2013. DOI: 10.2165/00019053-200826090-00002.

8. Ramsey SD, Willke RJ, Glick H, et al. Cost-effectiveness analysis alongside clinical trials II - An ISPOR good research practices task force report. *Value in Health* 2015; 18: 161–172.

9. Byford S, Leese M, Knapp M, et al. Comparison of alternative methods of collection of service use data for the economic evaluation health care interventions. *Health Econ* 2007; 16: 531–536.

10. NICE. *Guide to the methods of technology appraisal 2013*. National Institute for Health and Care Excellence, 2013.

11. Herdman M, Gudex C, Lloyd A, et al. Development and preliminary testing of the new five-level version of EQ-5D (EQ-5D-5L). *Qual Life Res* 2011; 20: 1727–36.

12. NICE. *Position statement on use of the EQ-5D-5L value set for England (updated October 2019)*. National Institute for Health and Care Excellence, 2019.

13. Van Hout B, Janssen MF, Feng YS, et al. Interim scoring for the EQ-5D-5L: Mapping the EQ-5D-5L to EQ-5D-3L value sets. *Value in Health* 2012; 15: 708–715.

14. Manca A, Hawkins N, Sculpher MJ. Estimating mean QALYs in trial-based cost-effectiveness analysis: The importance of controlling for baseline utility. *Health Econ* 2005; 14: 487–496.

15. Jennett B, Bond M. Assessment of outcome after severe brain damage. *The Lancet* 1975; 1: 480–484.

16. Teasdale GM, Pettigrew LEL, Wilson JTL, et al. Analyzing outcome of treatment of severe head injury: a review and update on advancing the use of the Glasgow Outcome Scale. *J Neurotrauma* 1998; 15: 587–597.

For peer review only - http://bmjopen.bmj.com/site/about/guidelines.xhtml

17. Wilson JTL, Slieker FJA, Legrand V, et al. Observer variation in the assessment of outcome in traumatic brain injury: experience from a multicenter, international randomized clinical trial. *Neurosurgery* 2007; 61: 123–128.

18. Wilson JTL, Pettigrew LEL, Teasdale GM. Structured interviews for the Glasgow Outcome Scale and the extended Glasgow Outcome Scale: guidelines for their use. *J Neurotrauma* 1998; 15: 573–580.

19. Faria R, Gomes M, Epstein D, et al. A Guide to Handling Missing Data in Cost-Effectiveness Analysis Conducted Within Randomised Controlled Trials. *Pharmacoeconomics* 2014; 32: 1157–1170.

20. Little R, Rubin D. *Statistical analysis with missing data*. 2nd ed. Hoboken, New Jersey: Wiley, 2002.

21. Teasdale G, Jennett B. Assessment of coma and impaired consciousness. A practical scale. *Lancet* 1974; 2: 81–4.

22. Teasdale G, Murray G, Parker L, et al. Adding up the Glasgow Coma Score. *Acta Neurochir Suppl (Wien)* 1979; 28: 13–6.

23. Willan AR, Briggs AH, Hoch JS. Regression methods for covariate adjustment and subgroup analysis for non-censored cost-effectiveness data. *Health Econ* 2004; 13: 461–475.

24. Claxton K. The irrelevance of inference: A decision-making approach to the stochastic evaluation of health care technologies. *J Health Econ* 1999; 18: 341–364.

25. Fenwick E, O'Brien BJ, Briggs AH. Cost-effectiveness acceptability curves-facts, fallacies and frequently asked questions. *Health Econ* 2004; 13: 405–415.

26. Ruggeri AG, Cappelletti M, Tempestilli M, et al. Surgical management of acute subdural hematoma: a comparison between decompressive craniectomy and craniotomy on patients treated from 2010 to the present in a single center. *J Neurosurg Sci* 2018; 66: 22–27.

27. Shibahashi K, Sugiyama K, Tomio J, et al. In-hospital mortality and length of hospital stay with craniotomy versus craniectomy for acute subdural hemorrhage: a multicenter, propensity score-matched analysis. *J Neurosurg* 2019; 133: 1–10.

28. Malmivaara K, Kivisaari R, Hernesniemi J, et al. Cost-effectiveness of decompressive craniectomy in traumatic brain injuries. *Eur J Neurol* 2011; 18: 656–662.

For peer review only

29. Ho KM, Honeybul S, Lind CRP, et al. Cost-effectiveness of decompressive craniectomy as a lifesaving rescue procedure for patients with severe traumatic brain injury. *J Trauma* 2011; 71: 1637–1644.

30. Whitmore RG, Thawani JP, Grady MS, et al. Is aggressive treatment of traumatic brain injury cost-effective? *J Neurosurg* 2012; 116: 1106–1113.

31. Alali AS, Naimark DMJ, Wilson JR, et al. Economic evaluation of decompressive craniectomy versus barbiturate coma for refractory intracranial hypertension following traumatic brain injury. *Crit Care Med* 2014; 42: 2235–2243.

32. Behranwala R, Aojula N, Hagana A, et al. An economic evaluation for the use of decompressive craniectomy in the treatment of refractory traumatic intracranial hypertension. *Brain Inj* 2021; 35: 444–452.

For peer review only

For peer review only - http://bmjopen.bmj.com/site/about/guidelines.xhtml

Table 1. Baseline characteristics of UK patients

**Characteristics**	**Craniotomy (N=126)**	**DC (N=122)**
Age (mean±SD) – yr	52.3±16.4	51.7±15.9
Male sex – N /total no. (%)	96/126 (76.2)	101/122 (82.8)
Any antithrombotic medication — N / n (%) [a]	21/115 (18.3)	22/110 (20.0)
Presence of major extracranial injury requiring admission — N / n (%)	66/123 (53.7)	57/120 (47.5)
Glasgow Coma Scale (GCS) 3-8*	85/120 (70.8)	72/119 (60.5)
Initial CT brain findings		
Presence of midline shift > 5mm — N / n (%)	106/124 (85.5)	105/121 (86.8)
Compression / absence of basal cisterns — N / n (%)	101/124 (81.5)	102/121 (84.3)
Presence of parenchymal contusions <25cc — N / n (%)	58/125 (46.4)	60/121 (49.6)

*A GCS score of 3–8 is defined as 'severe brain injury'; N=number of associated patients; n=number of patients for whom data were available

For peer review only - http://bmjopen.bmj.com/site/about/guidelines.xhtml

Table 2. Levels of resource use according to intervention arm over 12-month treatment period for all UK patients (based on available data)

Resource use	Craniotomy (N=126)	DC (N=122)	P-value‡
Hospital-recorded, index admission			
Primary intervention received, not as randomised, N	13 (n = 126)	8 (n = 122)	-
Duration of index surgery (hours), mean ± SD	2.57 ± 0.89 (n = 122)	2.50 ± 0.93 (n = 110)	0.603
ICU length of stay (index admission) (days), mean ± SD	11.85 ± 8.61 (n = 126)	13.52 ± 11.28 (n = 122)	0.189
NSU length of stay (index admission) (days), mean ± SD	16.75 ± 24.92 (n = 122)	21.30 ± 31.10 (n = 120)	0.210
Further DCs (index admission), N	15 (n=116)	4 (n = 116)	-
Further haematoma evacuations (index admission), N	9 (n = 116)	2 (n = 116)	-
Further wound revisions (index admission), N	1 (n = 116)	6 (n = 116)	-
Further other cranial operations (index admission)*, N	3 (n = 116)	2 (n = 2)	-
Hospital-recorded, 12-month follow-up (cranioplasties and shunts only)			
Primary cranioplasties, N	21 (n = 124)	62 (n = 121)	-
Cranioplasties requiring synthetic plate, N (%)	17 (81.0%) (n = 21)	46 (74.2%) (n = 62)	-
Cranioplasty revisions, N	5 (n = 124)	7 (n = 121)	-
Cranioplasties (primary/revisions) requiring re-admission, N	17 (n = 124)	58 (n = 121)	-
Primary shunts, N	5 (n = 126)	4 (n = 118)	-
Shunt revisions, N	5 (n = 126)	2 (n = 118)	-
Shunts (primary/revisions) requiring re-admission, N	4 (n = 126)	4 (n = 118)	-
Post-discharge cranioplasty/shunt related procedures (combined), N	21 (n = 124)	61 (n = 118)	-
Patient-reported, post-discharge			
Overnight stay with associated skull/brain operation, N	13 (n = 111)	32 (n = 95)	-

For peer review only - http://bmjopen.bmj.com/site/about/guidelines.xhtml

Any overnight stay excluding skull/brain operation, N reporting ≥1 stay	61 (n = 111)	54 (n = 95)	-
Overnight stay on rehabilitation unit,† (days), mean ± SD	32.51 ± 63.09 (n = 111)	35.10 ± 68.80 (n = 90)	0.782
Overnight stay on NSU,† (days), mean ± SD	0.49 ± 2.00 (n = 111)	1.14 ± 5.57 (n = 95)	0.252
Overnight stay on ICU,† (days), mean ± SD	0.13 ± 1.33 (n = 111)	0.07 ± 0.72 (n = 95)	0.731
Overnight stay on other ward,† (days), mean ± SD	4.94 ± 18.08 (n = 109)	3.04 ± 17.00 (n = 93)	0.447
Healthcare professional contact, N reporting ≥1 visit	64 (n = 109)	47 (n = 94)	-
Hospital doctor (visits), mean ± SD	0.60 ± 1.33 (n = 106)	0.61 ± 1.46 (n = 92)	0.980
Nurse (visits), mean ± SD	2.20 ± 16.53 (n = 107)	0.76 ± 5.28 (n = 92)	0.426
General Practitioner (visits), mean ± SD	1.23 ± 2.44 (n = 106)	1.09 ± 1.93 (n = 93)	0.656
Physiotherapist (visits), mean ± SD	2.38 ± 7.11 (n = 105)	4.03 ± 11.19 (n = 91)	0.213
Occupational therapist (visits), mean ± SD	1.56 ± 3.41 (n = 105)	2.22 ± 7.22 (n = 92)	0.407
Speech therapist (visits), mean ± SD	0.55 ± 2.29 (n = 107)	0.31 ± 1.40 (n = 90)	0.386
Social worker (visits), mean ± SD	0.16 ± 0.77 (n = 107)	0.12 ± 0.44 (n = 92)	0.665
Community care assistant (visits), mean ± SD	2.68 ± 21.44 (n = 106)	2.84 ± 20.45 (n = 92)	0.958
Emergency department (visits), mean ± SD	0.10 ± 0.53 (n = 107)	0.18 ± 0.61 (n = 93)	0.321
Psychologist/neuropsychologist (visits), mean ± SD	0.27 ± 1.24 (n = 107)	0.46 ± 2.72 (n = 93)	0.514
Other health care professional (visits), mean ± SD	0.03 ± 0.22 (n = 107)	0.04 ± 0.33 (n = 93)	0.699
Head/brain scan, N reporting ≥1 scan	47 (n = 111)	44 (n = 93)	-
MRI scans, mean ± SD	0.31 ± 0.62 (n = 111)	0.33 ± 0.56 (n = 93)	0.745
CT scans, mean ± SD	0.33 ± 0.67 (n = 111)	0.45 ± 0.73 (n = 93)	0.228
Other scans, mean ± SD	0.04 ± 0.19 (n = 111)	0.02 ± 0.15 (n = 93)	0.543

For peer review only - http://bmjopen.bmj.com/site/about/guidelines.xhtml

Patient-reported, post-discharge (wider resource use)			
Time in a care home (weeks), mean ± SD	1.79 ± 7.14 (n = 109)	3.53 ± 10.40 (n = 91)	0.164
Help from carer (hours), mean ± SD	971 ± 2,017 (n = 99)	1,000 ± 2,225 (n = 86)	0.925

DC= decompressive craniectomy; N=number of patients in receipt; n=number of patients for whom data were available; SD=standard deviation.
‡ for the mean cost difference between groups
*Excluding cranioplasties and shunts.
†Excluding those reported (by the patient) to be associated with a skull/brain operation (estimates were instead based on hospital-recorded data, see Table S1).
§Combines ‘Currently in paid/unpaid work’ with ‘hours working per week (paid or unpaid)’ and ‘reported return-to-work date’ to estimate mean hours worked per participant in 12 month follow-up period.

For peer review only

Table 3. Estimates of mean cost (UK £ sterling, 2018/19) by treatment group over 12-month treatment period for all patients (based on available data)

**Cost component**	**Craniotomy (N=126)**	**DC(N=122)**	**P-value‡**
Hospital-recorded, index admission			
Index neurosurgical procedure, mean cost ± SD	3,648 ± 1,264 (n=122)	3,560 ± 1,315 (n = 110)	0.603
Length of stay in NSU (index admission), mean cost ± SD	6,109 ± 9,085 (n = 122)	7,766 ± 11,339 (n = 120)	0.210
Length of stay in ICU (index admission), mean cost ± SD	20,039 ± 14,566 (n = 126)	22,873 ± 19,077 (n = 122)	0.189
Further DCs (index admission), mean cost ± SD*	307 ± 859 (n = 116)	82 ± 536 (n = 116)	0.017
Further haematoma evacuations (index admission), mean cost ± SD	165 ± 638 (n = 116)	37 ± 279 (n = 116)	0.048
Further wound revision (index admission), mean cost ± SD	18 ± 198 (n = 116)	110 ± 551 (n = 116)	0.092
Further other cranial operations (index admission),† mean cost ± SD	55 ± 340 (n = 116)	37 ± 279 (n = 116)	0.653
**Total cost per patient (index admission), mean cost ± SD**	**30,790 ± 19,710 (n = 109)**	**34,759 ± 24,481 (n = 102)**	**0.195**
Hospital-recorded cranioplasties and shunts (index admission and post-discharge)			
Cranioplasty procedures, mean cost ± SD	1,059 ± 2,485 (n = 124)	3,055 ± 3,352 (n = 122)	<0.0001
Shunt procedures, mean cost ± SD	212 ± 1,121 (n = 126)	150 ± 834 (n = 118)	0.626
Cranioplasty/shunt same day discount, mean cost ± SD §	-17 ± 132 (n = 124)	0 ± 0 (n = 118)	0.167
**Total cost per patient (cranioplasties and shunts), mean cost ± SD**	**1,258 ± 2,983 (n = 124)**	**3,228 ± 3,677 (n = 118)**	**<0.0001**
Patient-reported, post-discharge			
Overnight stays on rehabilitation unit, mean cost ± SD**	16,375 ± 31,784 (n = 111)	17,677 ± 34,660 (n = 90)	0.782
Overnight stays on NSU, mean cost ± SD**	177 ± 729 (n = 111)	415 ± 2,029 (n = 95)	0.252
Overnight stays on ICU/HDU, mean cost ± SD**	213 ± 2,247 (n = 111)	125 ± 1,215 (n = 95)	0.731
Overnight stays on 'other' ward, mean cost ± SD**	1,746 ± 6,396 (n = 109)	1,076 ± 6,015 (n = 93)	0.447
All healthcare professional visits, mean cost ± SD	682 ± 1,108 (n = 103)	782 ± 1,578 (n = 88)	0.612

For peer review only - http://bmjopen.bmj.com/site/about/guidelines.xhtml

All head/brain scans, mean cost ± SD	66 ± 105 (n = 111)	93 ± 101 (n = 93)	0.436
**Total cost per patient (post-discharge PSRQ), mean cost ± SD**	**19,699 ± 34,193 (n = 99)**	**17,948 ± 32,183 (n = 81)**	**0.726**
Time in a care home (wider perspective only), mean cost ± SD	3,321 ± 13,230 (n = 109)	6,550 ± 19,272 (n = 91)	0.164
Carer time (wider perspective only), mean cost ± SD	16,762 ± 34,828 (n = 99)	17,271 ± 38,419 (n = 86)	0.925
**Overall NHS and PSS cost per patient, mean cost ± SD**	**48,509 ± 46,934 (n = 86)**	**53,573 ± 47,092 (n = 67)**	**0.510**

n=number of patients for whom data were available; SD=standard deviation; PSS=Personal Social Services; DC= decompressive craniectomy
‡ for the mean cost difference between groups
*Based on mean duration of DC (from all index procedures) of 2.5042 (n=110) hours for all randomized patients.
†Excluding cranioplasties and shunts.
§A discount was applied to account for those shunt and cranioplasty procedures that occurred on the same day and were therefore assumed to be associated with a slightly shorter operation duration and NSU stay.
**Overnight stays excluding those associated with a skull/brain operation.

For peer review only - http://bmjopen.bmj.com/site/about/guidelines.xhtml

Table 4. Estimates of mean outcomes by treatment group over 12-month treatment period for all patients (based on available case)

Item	Craniotomy (N=126)	DC (N=122)	P-value‡
Baseline EQ-5D-5L score, mean ± SD	0.260 ± 0.353 (n = 87)	0.302 ± 0.366 (n = 91)	0.441
6-month EQ5D-5L score, mean ± SD	0.427 ± 0.392 (n = 102)	0.370 ± 0.393) (n = 94)	0.311
6-month change in EQ5D-5L score, mean ± SD	0.184 ± 0.345 (n = 74)	0.073 ± 0.319 (n = 71)	0.046
12-month EQ5D-5L score, mean ± SD	0.471 ± 0.402 (n = 111)	0.336 ± 0.414 (n = 103)	0.016
12-month change in EQ5D-5L score, mean ± SD	0.218 ± 0.367 (n = 79)	0.073 ± 0.361 (n = 78)	0.013
Total QALY score, mean ± SD	0.351 ± 0.335 (n = 68)	0.338 ± 0.366 (n = 64)	0.830
12-month GOSE score, % favourable**	47.9 (n = 121)	37.4 (n = 115)	0.102

n=Number for whom data were available; SD=standard deviation; QALY=Quality Adjusted Life Years. GOSE= Glasgow Outcome Scale Extended; GCS= Glasgow Coma Score.
‡ for the mean difference between groups
*Favourable for the GCS score was defined as 9–15 points (moderate to minor brain injury) while unfavourable was defined as 3–8 points (severe brain injury)
**Favourable for the GOSE score was defined as upper severe disability or better.
†If GCS at baseline is between 3 and 8, a favourable outcome will be defined as upper severe disability or better on 12-month GOSE. If GCS at baseline is between 9 and 15, a favourable outcome will be defined as lower moderate disability or better on 12-month GOSE.

For peer review only

For peer review only - http://bmjopen.bmj.com/site/about/guidelines.xhtml

Table 5. Estimates of the mean incremental cost, incremental effect (QALY gain or odds ratio), and cost effectiveness of craniotomy compared with DC in the base-case and two sensitivity analyses

**Cost utility analysis (N craniotomy, N DC)**	**Incremental cost (95% CI)**	**QALY gain (95% CI)**	**ICER**	**CEAC***
Base-case: imputed (126,122)	-£5,520<br>(-£18,060 to £7,020)	0.093<br>(0.029 to 0.156)	Dominant	87%
SA wider cost perspective (126,122)	-£17,793<br>(-34,658 to -928)	0.094<br>(0.030 to 0.159)	Dominant	99%
SA lowest EQ-5D baseline score (126,122)	-£5,445<br>(-£17,547 to £6,658)	0.089<br>(0.025 to 0.152)	Dominant	87%
**Cost effectiveness analysis (N craniotomy,N DC)**	**Incremental cost (95% CI)**	**Odds ratio (95% CI)**	**Cost per favourable outcome**	
Base-case: imputed 12m GOSE (126,122)	-£4,536<br>(-17,374 to £8,301)	1.682<br>(0.995 to 2.842)	Dominant	-
SA wider cost perspective 12m GOSE (126,122)	-16,900<br>(-£33,807 to £7)	1.693<br>(0.998 to 2.871)	Dominant	-

95% CI=95% confidence interval; ICER =incremental cost-effectiveness ratio; Dominant = lower mean costs and higher mean effect; N crandiotomy (N DC) = number randomised to craniotomy/decompressive craniectomy who were included in the analysis; SA:sensitivity analysis, described in the Methods; QALY=Quality Adjusted Life Years;
*Probability of being cost-effective on the cost-effectiveness acceptability curve (CEAC) at a threshold of £20,000 per QALY.

For peer review only - http://bmjopen.bmj.com/site/about/guidelines.xhtml

SUPPLEMENTAL MATERIAL (TABLES)

Supplemental Table S1. Unit costs, for the 2018/19 financial year

Resource use	Unit cost (£)	Assumptions
Neurosurgical costs		
Index craniotomy or DC (hourly rate)	1,422[1]	Hourly rate applied to the duration of the operation, whether craniotomy or DC. Includes the time from entering pre-med until leaving theatre.
DC, not index procedure (hourly rate)	1,422[1]	Hourly rate applied to two-thirds of the mean length recorded for index DC. This accounts for the presence of previous skin incision and bone cuts.
Cranioplasty (operation cost, index or revision)	2,464[1,2]	Based on hourly rate above and 104 min duration, with an additional cost for both any synthetic material (if applicable, see below) and an additional associated NSU length of stay of 4 days if post-discharge (see below rates).
Haematoma evacuation (all types)	2,132[1]	Based on hourly rate above and 90 min duration (expert opinion)
Wound revision	2,132[1]	Based on hourly rate above and 90 min duration (expert opinion)
'Other' neurosurgical intervention	2,132[1]	Based on hourly rate above and 90 min duration (expert opinion)
Shunt placement (index or revision)	2,132[1]	Based on hourly rate above and 90 min duration (expert opinion) with an additional material cost (see below) and an associated NSU length of stay of 2 days if post-discharge
Synthetic material costs (design/parts) for cranioplasty	2,500	Estimated based on expert opinion (only added if the use of synthetic material was indicated on the relevant form). Not applicable for revisions.
Material costs for shunt	500	Estimated based on expert opinion. Not applicable for revisions.
Over-night stay costs		
Cost per bed day in Neuro-rehabilitation unit	504[3]	
Cost per bed day in NSU	365[4,5]	
Cost per bed day in ICU	1,691[6]	Assumes neurosciences adult patient in critical care, 2 or more organs supported (ICU)
Cost per bed day (other ward type)	354[4,5]	Weighted average of elective and non-elective excess bed days

Health professional visit costs	*Community*	*Hospital*	*Home*	*Assumptions*

For peer review only

Hospital doctor	33.00[4]	186.74[6]	59.40[4,7]	Community: as hospital doctors do not work in the community, the unit cost for a community GP visit was applied. Home: as hospital doctors do not usually visit homes, the unit cost for a home GP visit was applied.
Nurse	12.31[4,7]	69.51[6]	19.64[4,7]	Home: costed as for community visit, plus 12 mins travel time
General Practitioner	33.00[4]	186.74[6]	59.40[4,7]	Hospital: as GPs do not work in hospitals, the unit cost for a hospital doctor visit was applied. Home: costed as for community visit, plus 12 mins travel time.
Physiotherapist	62.90[6]	54.96[6]	69.67[4,6,7]	Home: costed as for community visit, plus 12 mins travel time.
Occupational therapist	83.17[6]	65.54[6]	89.94[4,6,7]	Home: costed as for community visit, plus 12 mins travel time.
Speech therapist	106.51[6]	100.06[6]	113.28[4,6,7]	Home: costed as for community visit, plus 12 mins travel time
Social worker	118.81[4,8]	118.81[4,8]	127.72[4,7,8]	Home: costed as for community visit, plus 12 mins travel time.
Community care assistant	19.87[4,9]	19.87[4,9]	24.64[4,7,9]	Home: costed as for community visit, plus 12 mins travel time.
Emergency department	166.05[6]	166.05[6]	166.05[6]	Single rate costed for an emergency visit
Psychologist/neuropsychologist	141.17[4,10]	146.67[4,10]	156.57[4,7,10]	Home: costed as for community visit, plus 12 mins travel time.
Other	33.00[4]	186.74[6]	69.67[4,6,7]	The cost of the most commonly reported visits from each location are assigned. Community: GP, Hospital: hospital doctor, home: physiotherapist
Other costs				*Assumptions*
MRI scan		120.83[6]		
CT scan		77.95[6]		
Unknown scan		77.95[6]		Assumed the cost of a CT scan
Care home (cost per week in residence)		1,854[11]		As no cost for adults with these specific needs has been estimated, we have used a cost for adults with autism and complex needs.
Carer time		17.27[12]		Gross hourly rate. Used to value carer time whether paid or not
Work time		17.27[12]		Gross hourly rate. Used to value lost work time, assigned to estimated time worked since their brain injury

DC, decompressive craniectomy; ICU, intensive care unit; NSU, neurosurgical care unit; MRI, magnetic resonance imaging; CT, computed tomography
Inflated to 2018/19 financial year prices, where necessary, using the NHSCII pay and prices.[4]

For peer review only

Supplemental Table S2. Proportion of Missing values (%) for key variables

Variable	Craniotomy	DC	Total
Baseline variables			
Treatment allocation	0	0	0
Age	0	0	0
Sex	0	0	0
EQ-5D at baseline	39/126 (31.0%)	31/122 (25.4%)	70/248 (28.2%)
GCS score	6/126 (4.8%)	3/122 (2.5%)	9/248 (3.6%)
Cost variables			
Index admission costs (hospital-recorded data)*	17/126 (13.5%)	20/122 (16.4%)	37/248 (14.9%)
Cranioplasty and shunt costs (hospital-recorded data)†	2/126 (1.6%)	4/122 (3.3%)	6/248 (2.4%)
Post-discharge costs (patient self-report data)	27/126 (21.4%)	41/122 (33.6%)	68/248 (27.4%)
Outcome variables for health-related quality of life			
EQ-5D at 6 months	24/126 (19.1%)	28/122 (23.0%)	52/248 (21.0%)
EQ-5D at 12 months	15/126 (11.9%)	19/122 (15.6%)	34/248 (13.7%)
Outcome variables for GOSE			
GOSE at 6 months	13/126 (10.3%)	16/122 (13.1%)	29/248 (11.7%)
GOSE at 12 months	5/126 (4.0%)	7/122 (5.7%)	12/248 (4.8%)
Outcomes for cost-utility and cost-effectiveness analyses			
Total costs	40/126 (31.8%)	55/122 (45.1%)	95/248 (38.3%)
Total QALYS	58/126 (46.0%)	58/122 (47.5%)	116/248 (46.8%)
Binary GOSE at 12 months	5/126 (4.0%)	7/122 (5.7%)	12/248 (4.8%)
Binary GOSE dependent on GCS at 12 months	11/126 (8.7%)	10/122 (8.2%)	21/248 (8.5%)

*Includes index surgery, length of stay, neurosurgical interventions (excluding cranioplasties and shunts) during index admission.
†Includes cranioplasties and shunts (including revisions) during index admission and post-discharge.

For peer review only

References

1. Public Health Scotland. *Theatre - direct cost per hour by speciality*. Edinburgh, 2019.

2. Fountain DM, Henry J, Honeyman S, et al. First Report of a Multicenter Prospective Registry of Cranioplasty in the United Kingdom and Ireland. *Neurosurgery* 2021; 89: 518–526.

3. Turner-Stokes L, Williams H, Bill A, et al. Cost-efficiency of specialist inpatient rehabilitation for working-aged adults with complex neurological disabilities: a multicentre cohort analysis of a national clinical data set. *BMJ Open* 2016; 6: e010238.

4. Curtis L, Burns A. *Unit costs of health and social care 2019*. Personal Social Services Research Unit, University of Kent, Canterbury, 2019. Epub ahead of print 2019. DOI: 10.22024/UniKent/01.02.79286.

5. Health and social Care Information Centre. *NHS Schedule of Reference Costs 2017/18*. London: Department of Health, 2018.

6. Health and Social Care Information Centre. *NHS Schedule of Reference Costs 2018/19*. London: Department of Health, 2019.

7. Curtis L, Burns A. *Unit costs of health and social care 2015*. Personal Social Services Research Unit, University of Kent, Canterbury, 2015.

8. Curtis L. *Unit costs of health and social care 2010*. Personal Social Services Research Unit, University of Kent, Canterbury, 2010.

9. Curtis L, Burns A. *Unit costs of health and social care 2017*. Personal Social Services Research Unit, University of Kent, Canterbury, 2017. Epub ahead of print 2017. DOI: 10.22024/UniKent/01.02/65559.

10. Curtis L, Burns A. *Unit costs of health and social care 2016*. Personal Social Services Research Unit, University of Kent, Canterbury, 2016.

For peer review only

11. Curtis L, Burns A. *Unit costs of health and social care 2018*. Personal Social Services Research Unit, University of Kent, Canterbury, 2018. Epub ahead of print 2018. DOI: 10.22024/UniKent/01.02.70995.

12. Office for National Statistics. *Annual Survey of Hours and Earnings (ASHE) Table 1 (revised)*, https://www.ons.gov.uk/employmentandlabourmarket/peopleinwork/earningsandworkinghours/datasets/allemployeesashetable1 (2019).

For peer review only

SUPPLEMENTAL MATERIAL (APPENDICES)

APPENDIX 1:

AUTHOR AFFILIATIONS: Norwich Medical School, University of East Anglia, Norwich (Sarah C. Pyne, Garry R. Barton, David A Turner), the Division of Neurosurgery, Addenbrooke's Hospital (Angelos G. Kolias, Carole Turner, Hadie Adams, Midhun Mohan, Edoardo Viaroli, Adel E. Helmy, Ivan S. Timofeev, Peter J. Hutchinson), the Department of Clinical Neurosciences (Harry Mee, Angelos G. Kolias, Carole Turner, Hadie Adams, Midhun Mohan, Matthew G. Stovell, Edoardo Viaroli, Adel E. Helmy, Ivan S. Timofeev, Peter J. Hutchinson,), and the Division of Anaesthesia (David K. Menon), University of Cambridge, and the Cambridge Clinical Trials Unit, Cambridge University Hospitals NHS Foundation Trust (Carole Turner), Cambridge, the Neurosurgery Department, Royal London Hospital (Christopher Uff, Shumaila Hasan), the Department of Neurosurgery, Imperial Neurotrauma Centre, Imperial College Academic Health Sciences Centre, St. Mary's Hospital (Mark H. Wilson), London, Wessex Neurological Centre, University Hospitals Southampton NHS Foundation Trust, Southampton (Diederik Bulters, Ardalan Zolnourian), the Department of Neurosurgery, Walton Centre NHS Foundation Trust, Liverpool (Catherine J. McMahon, Matthew G. Stovell), the Department of Neurosurgery and the Academic Directorate of Neurosciences, Sheffield Teaching Hospital NHS Foundation Trust, Sheffield (Yahia Z. Al-Tamimi), the Department of Neurosurgery, Leeds General Infirmary, Leeds (Simon Thomson), the Department of Neurosurgery, University Hospitals Birmingham NHS Foundation Trust, Birmingham (Antonio Belli), the Department of Neurosurgery, Manchester Centre for Clinical Neurosciences, Manchester (Andrew T. King), and the Neurosurgical Trials Group, Wolfson Research Centre, Newcastle University, Newcastle upon Tyne (Barbara Gregson) — all in the United Kingdom.

INVESTIGATORS AND TRIAL OVERSIGHT COMMITTEES

<u>RESCUE-ASDH trial collaborators (to be indexed on PubMed):</u>

- Peter J. Kirkpatrick, Hadie Adams, Nicola Owen, Kirsty Grieve, Alexis Joannides, Ellie Edlmann, Karen Caldwell, Silvia Tarantino, Francesca Hill, Liz Corteen, Janet Corn, Matthew G Stovell, Mathew Guilfoyle, Mohammad Naushahi, Richard Mair, Ibrahim Jalloh, Mark Kotter, Kamila Walker, Tamara Tajsic, Sara Venturini, Selma Tülü – *Cambridge University Hospital, Cambridge, UK*
- Christopher Uff, Shumaila Hasan, Chipo Chitsenega, Geetha Boyapati – *Royal London Hospital, London, UK*

For peer review only

- Muhammad Bhatti, Malik Zaben, Belinda Gunning, Natalia Ermalai, Dmitri Shastin, Mutwakil Abdulla, Liudmila, Joseph Merola – *University Hospital of Wales, Cardiff, UK*
- Stuart Smith, Laurence Glanz, Ashwin Kumaria, Lani Patterson – *Queen's Medical Centre, Nottingham, UK*
- Antonio Belli – *1. NIHR Surgical Reconstruction and Microbiology Research Centre, Birmingham, UK. 2. Institute of Inflammation and Ageing, University of Birmingham, Birmingham, UK*
- Colin Bergin, Maximina Ventura, Emma Toman – *NIHR Surgical Reconstruction and Microbiology Research Centre, Birmingham, UK*
- Giles Critchley, Laura Ortiz-Ruiz de Gordoa, Prasanna Epaliyanage, Husam Georges – *Royal Sussex County Hospital, Brighton, UK*
- Peter Whitfield, Sam Jeffrey, Charlotte Eglinton, Natasha Wilmhurst – *Derriford, Hospital, Plymouth, UK*
- Manjunath Prasad, Shahid Khan, Philip Kane, Emanuel Cirstea – *James Cook University Hospital, Middlesbrough, UK*
- Catherine McMahon, Geraint Sunderland, John Kitchen, Rushid Zakaria – *The Walton Centre, Liverpool, UK*
- Simon Thomson, Soumya Mukherjee, Ian Anderson, Linetty Makawa, Mary Kambafwile – *Leeds General Infirmary, Leeds, UK*
- Marios C. Papadopoulos, Mathew Joseph Gallagher, Siobhan Kearney, Sarah Trippier – *St. George's Hospital, London – UK*
- Mark Wilson, Hani Marcus, Rhys Thomas, Sonia Fernandez Lopez, Louise Young, Andrea D'Mello, Jo-Anna Conyngham – *St. Mary's Hospital, London, UK*
- Diederik Bulters, Ardalan Zolnourian, Patrick Holton, Miriam Taylor, Charlaine Reeve – *Southampton University Hospital, Southampton, UK*
- Jonathan Pollock, Ciaran Hill, Elisa Visentin, Laura Parker – *Queen's Hospital, Romford, UK*
- Kismet Hossain-Ibrahim, Paul Johnston – *Ninewells Hospital, Dundee, UK*
- Vasileios Arzoglou, Emma Clarkson, Arif Zafar, Efosa Ukponmwan – *Hull Royal Infirmary, Hull, UK*
- Christos Tolias, Joseph Frantzias, Anastasios Giamouriadis – *King's College London, London, UK*
- Yahia Al-Tamimi, Adam Wahba, Patrick Easton, Rose Clegg, Grace Cole – *Royal Hallamshire Hospital, Sheffield, UK*
- Damian Holliman, Louise Finlay – *Royal Victoria Infirmary, Newcastle, UK*

For peer review only

- Daniel Holsgrove, Alex Leggate, Jane Perez, Louise Harrison – *Salford Royal Hospital, Manchester, UK*
- Gareth Roberts, Terrie-Louise Cromie, Marianne Hare, Sonia Raj – *Royal Preston Hospital, Preston, UK*
- Tim Lawrence, Ruichong Ma, Javier Magan Ventura, Neil Davidson – *John Radcliffe Hospital, Oxford, UK*
- Richard Nelson, Ruth Womer, Beverley Fulkner – *Frenchay Hospital/North Bristol NHS Trust, Bristol, UK*
- Shabin Joshi, Geraldine Ward – *Walsgrave General Hospital, Coventry, UK*
- Ioannis Fouyas, Julie Woodfield, Himanshu Shekhar, Kareen Damley, Emma Fleming – *Western General Hospital, Edinburgh, UK*
- Roddy O'Kane, Michael Canty – *Queen Elizabeth University Hospital, Glasgow, UK*
- Indira Devi Bhagavatula, Dhananjaya Ishwar Bhat, Dhaval Prem Shukla, Kanti Konar, Nagesh Shanhag, Vaishali NI Valluri – *National Institute of Mental Health and Neurosciences (NIMHANS), Bangalore, India*
- Manoj Kumar Tewari, Manjul Tripathi – *Post Graduate Institute of Medical Education and Research (PGIMER), Chandigarh, India*
- Deepak Gupta, Ashish Bindra, Kaveri Sharma – *All India Institute of Medical Sciences (AIIMS), Delhi, India*
- Mathew Joseph – *Christian Medical College, Vellore, India*
- Nicole Keong, Christine Lock, Chen Min Wei, Julian Han, Janell Kwok, Nicolas Kon Kam King – *National Neuroscience Institute, Singapore*
- Martina Stippler, Emmalin Nelton – *Beth Israel Medical Center, USA*
- Louis Anthony Whitworth, Sonja Stutzman, Christopher Madden, Caryn Harper, Tracey Moore, Vin Shen Ban, Alice Salazar – *The University of Texas Southwestern Medical Center, USA*
- Rocco Armonda, Jason J. Chang, George B. Moses, Patricia Tanjucto – *MedStar Washington Hospital Center, Washington, USA*
- Jamie Ullman, Orseola Arapi, Betsy Moclair – *Northwell Shore University Hospital (Northwell Health), New York, USA*
- Nrupen Baxi, Erin Lewis, Melvin Stone – *Jacobi Medical Center and Montefiore Medical Center, New York, USA*
- John Adair Prall, Meghan Baldwin, Jamie Jones – *Littleton Adventist Hospital (Centura Health), Colorado, USA*
- Clare Gallagher, Ish Bains – *Foothills Medical Centre, Calgary, Canada*
- Kesava Reddy, Paula Carroll – *Hamilton Health Sciences, Ontario, Canada*

For peer review only

- Leodante Da Costa, Nadia Scantlebury – *Sunnybrook Hospital, University of Toronto, Toronto, Canada*
- Fahad Alkherayf, Rafael Ochoa Sanchez, Ioana Moldovan – *The Ottawa Hospital, Ottawa, Canada*
- Sean Christie, Lisa Julien – *Queen Elizabet II Health Sciences Centre, Halifax, Canada*
- Kostas Fountas, Thanasis Paschalis – *University Hospital of Larissa, Medical School (University of Thessaly), Larissa, Greece*
- Sandro Krieg, Sebastian Ille – *Technische Universität München, Munich, Germany*
- Maria Luisa Gandia Gomez – *Hospital Universitario La Paz, Madrid, Spain*
- Alfonso Legares, Ana Maria Castaño Leon – *Hospital Universitario 12 de Octubre, Madrid, Spain*
- András Büki, Gábor Lenzsér – *University of Pécs, Pécs, Hungary*
- Tariq Khan, Mukhtar Khan – *Northwest General Hospital and Research Center, Peshawar, Pakistan*
- Franco Servadei, Massimo Tomei – *Humanitas Research Hospital, Milan, Italy*
- Vairavan Narayanan, Ronie Romelean Jayapalan – *University of Malaya Medical Centre, Kuala Lumpur, Malaysia*
- Martin Hunn, Panagiota Gkolia, Emily Galea – *The Alfred Hospital, Melbourne, Australia*

Trial co-applicants and/or protocol contributors (to be indexed on PubMed):
Clare Gallagher, Paula Kareclas, Sridevi Nagarajan, David Turner, James Piercy, Peter Kirkpatrick, A. David Mendelow, Andrew King, Antonio Belli, John D. Pickard, Paul Brennan, Christopher Cowie

Cambridge Clinical Trials Unit staff (to be indexed on PubMed):
Carol Davis-Wilkie, Tapiwa Tungamirai, Kerstin Wolf, Kamila Walker, Natalia Igosheva, Alicia Gore, Paula Kareclas, Michele Jillings, Christopher Bushell

Trial Steering Committee members (to be indexed on PubMed):
Anthony Bell (chair), Allison Hirst, Peter McCabe, Simon Shaw

Independent Data Monitoring and Ethics Committee members (to be indexed on PubMed):
Martin Smith (chair), Joan Grieve, Jonathan Cook

For peer review only

APPENDIX 2: BASELINE GCS-ADJUSTED GOSE (SLIDING-DICHOTOMY ANALYSIS)

Methods

In addition to the extended Glasgow Outcome Scale (GOSE) fixed dichotomy analysis described in the main paper, a further GOSE sliding dichotomy analysis was undertaken, in which the favourable/unfavourable categorisation was defined as follows: if GCS (Glasgow Coma Scale) at randomisation was between 3 and 8 (patient comatosed) (1), a favourable outcome was defined as upper severe disability or better but if GCS at randomisation was between 9 and 15 (responsive patient), a favourable outcome was defined as lower moderate disability or better. The cost-effectiveness analysis using this sliding dichotomy, replicated that described in the main paper for the fixed dichotomy, with a view to estimating the cost per additional favourable outcome.

Results and conclusions

For the craniotomy group compared with the DC group, the mean difference in cost was -£6,091 (95% CI -£18,857 to £6,675) with an odds ratio of favourable outcome on the GOSE score of 1.741 (95% CI 1.019 to 2.977). Craniotomy therefore dominated DC.

For peer review only

APPENDIX 3: FURTHER SENSITIVITY ANALYSES

Methods

In addition to the two sensitivity analyses described in the main paper, a further four were defined in the Health Economic Analysis Plan (HEAP) and analysed. The consequence of excluding patient self-reported resource use data (more missing data was expected from this source), and using only hospital-recorded costs was assessed as a sensitivity analysis (SA hospital-recorded post-discharge operations only). Another sensitivity analysis (SA patient-reported post-discharge operations only) included only patient-reported post-discharge skull/brain operations (with associated length of stay) instead of hospital-reported post-discharge cranioplasties and shunts. A further sensitivity analysis (SA per protocol) re-analysed the data on a per protocol basis, excluding patients whose primary treatment was not as allocated, e.g. allocated to DC but received craniotomy and vice versa. A complete case analysis based on the base-case was also undertaken (SA complete case analysis), where participants were only included if they have complete hospital records, participant self-report and QALY data, with no imputation undertaken. These sensitivity analyses were undertaken for both the cost-utility analysis and cost-effectiveness analysis.

Results and Conclusions

The results of the four sensitivity analyses described are presented in Table A1. In all sensitivity analyses, craniotomy was found to dominate DC. This is in keeping with the base-case cost-utility and cost-effectiveness analyses and other sensitivity analyses presented in this paper.

For peer review only

Supplemental Table A1 | Estimates of the mean incremental cost, incremental effect (QALY gain or odd ratio), and cost effectiveness of craniotomy compared with DC for additional sensitivity analyses.

Analysis (N craniotomy,N DC)	Incremental cost (95% CI)	QALY gain (95% CI)	ICER	CEAC*
SA hospital-recorded post-discharge operations only: (126,122) MI	-£6,252 (-£12,180 to -£325)	0.092 (0.031 to 0.153)	Dominant	99%
SA patient-reported post-discharge operations only: (126,122) MI	-£6,328 (-19,389 to £6,733)	0.093 (0.032 to 0.154)	Dominant	89%
SA per protocol: (113,114) MI	-£10,711 (-£23,361 to £1,939)	0.121 (0.056 to 0.185)	Dominant	98%
SA complete case analysis: (60,44)	-£1,917 (-£15,564 to £11,729)	0.071 (-0.0106 to 0.153)	Dominant	68%
**Analysis (N craniotomy,N DC)**	**Incremental cost (95% CI)**	**GOSE odds ratio (95% CI)**	**Cost per favourable outcome**	
SA hospital-recorded post-discharge operations only: (126,122)	-£5,709 (-£11,783 to £365)	1.704 (1.010 to 2.888)	Dominant	-
SA patient-reported post-discharge operations only: (126,122)	-£5,374 (-£18,782 to £8,033)	1.687 (0.999 to 2.849	Dominant	-
SA per protocol: (113,114)	-£10,567 (-£23,434 to £2,299)	2.189 (1.252 to 3.827)	Dominant	-
SA complete case analysis: (83,67)	-£4,335 (-£18,545 to £9,876)	1.360 (0.698 to 2.649)	Dominant	-

95% CI=95% confidence interval; ICER =incremental cost-effectiveness ratio; Dominant = lower mean costs and higher mean effect; N crandiotomy (N DC) = number Randomized to craniotomy/decompressive craniectomy who were included in the analysis; SA:sensitivity analysis, described in the Methods; QALY=Quality Adjusted Life Years;
*Probability of being cost-effective on the CEAC at a threshold of £20,000 per QALY

For peer review only

APPENDIX 4: DEVIATION FROM THE HEAP IN “SA WIDER COST PERSPECTIVE”

Within the Health Economic Analysis Plan (HEAP) it was stated that lost productivity costs would be estimated. Below we explain why this was not undertaken.

The following was stated within the ‘Costs’ section for the HEAP:

“…Participants were asked to report a) whether they were currently working (paid or unpaid), with the following additional questions (if applicable); b) how many hours per week they work (paid or unpaid); c) whether the number of hours was the same as before their brain injury; d) whether they currently work fewer or more hours per week than before your brain injury; e) when they returned to work following the brain injury; f) whether they have taken any days off due to sickness since returning; g) if they have had to leave work / change job since their brain injury and why. In order to estimate lost productivity, in line with the opportunity cost method (2), the mean lost work time over the 12 month follow-up period (regardless of whether a payment was made) will be estimated and valued at the 2019 UK mean hourly gross wage (£17.25) (3)…“

Within the ‘Analysis’ section for the HEAP we stated that the base-case analysis would be from the cost perspective of the NHS and PSS. However, it was stated that the first sensitivity analysis (SA) (“SA wider cost perspective” in this paper) would take a more societal perspective and include lost productivity costs, as well as care home and carer costs.

We attempted to include lost productivity costs at the analysis stage but found that we did not have information as to the number of hours participants were working before their brain injury, as intended. The main reason for this was that if a participant reported that they were *not* currently working in response to the above question a) they were not asked to complete questions b-f. In hindsight, this was an error in how the questionnaire was formulated, and they should have been asked to complete questions c and d as well. Considering this error in the framing of the questionnaire we chose to deviate from the HEAP and not estimate lost productivity costs. Consequently, as detailed in the paper, in “SA wider cost perspective” only the care home and carer costs were added to the (base-case) NHS and PSS costs.

For peer review only

References

1. Bullock MR, Chesnut R, Ghajar J, et al.: Surgical management of acute subdural hematomas. *Neurosurgery* 2006; 58:S2-16-S2-24

2. Hoefman RJ, Van Exel J, Brouwer W: How to include informal care in economic evaluations. *Pharmacoeconomics* 2013; 31:1105–1119

3. Office for National Statistics: Annual Survey of Hours and Earnings (ASHE) Table 1 (revised) [Internet]. 2019. Available from: https://www.ons.gov.uk/employmentandlabourmarket/peopleinwork/earningsandworkinghours/datasets/allemployeesashetable1

For peer review only

CHEERS 2022 Checklist

Topic	No.	Item	Location where item is reported
**Title**			
	1	Identify the study as an economic evaluation and specify the interventions being compared.	Title page: "Cost-effectiveness of craniotomy versus decompressive craniectomy, for patients with traumatic acute subdural hematoma"
**Abstract**			
	2	Provide a structured summary that highlights context, key methods, results, and alternative analyses.	See Abstract
**Introduction**			
**Background and objectives**	3	Give the context for the study, the study question, and its practical relevance for decision making in policy or practice.	See the 'Background' section
**Methods**			
**Health economic analysis plan**	4	Indicate whether a health economic analysis plan was developed and where available.	In the 'sensitivity analyses' section of the Methods we state that there was "…a pre-specified health economic analysis plan (HEAP) (see: https://www.rescueasdh.org/trial-documents)."
**Study population**	5	Describe characteristics of the study population (such as age range, demographics, socioeconomic, or clinical characteristics).	See the 'Participants' section of the Results and Table 1.
**Setting and location**	6	Provide relevant contextual information that may influence findings.	See the 'Participants' section of the Methods
**Comparators**	7	Describe the interventions or strategies being compared and why chosen.	The interventions are described in the 'Treatment and randomisation' section of the Methods. The rationale is covered in the 'Background' section

For peer review only - http://bmjopen.bmj.com/site/about/guidelines.xhtml

Topic	No.	Item	Location where item is reported
**Perspective**	8	State the perspective(s) adopted by the study and why chosen.	Costs were estimated from a UK National Health Service (NHS) and Personal and Social Services (PSS) perspective, as stated in the 'Measuring Costs' section of the Methods
**Time horizon**	9	State the time horizon for the study and why appropriate.	12 month follow-up period (which aligns for that for the trial) is stated in both the 'Measuring Costs' and 'Measuring Outcomes' section of the Methods
**Discount rate**	10	Report the discount rate(s) and reason chosen.	Given the 12 month follow-up period, no discounting was undertaken, as stated in the 'Incremental analyses' section of the Methods
**Selection of outcomes**	11	Describe what outcomes were used as the measure(s) of benefit(s) and harm(s).	See the 'Measuring outcomes' section of the Methods
**Measurement of outcomes**	12	Describe how outcomes used to capture benefit(s) and harm(s) were measured.	See the 'Measuring outcomes' section of the Methods
**Valuation of outcomes**	13	Describe the population and methods used to measure and value outcomes.	See the 'Measuring outcomes' section of the Methods
**Measurement and valuation of resources and costs**	14	Describe how costs were valued.	See the 'Measuring costs' section of the Methods and Supplemental Table S1 for unit costs
**Currency, price date, and conversion**	15	Report the dates of the estimated resource quantities and unit costs, plus the currency and year of conversion.	The dates of the estimated resource quantities are reported in the 'Participants' section of the Results section. Other items are reported in the 'Measuring costs' section of the same chapter
**Rationale and description of model**	16	If modelling is used, describe in detail and why used. Report if the model is publicly available and where it can be accessed.	Not applicable, a within trial cost effectiveness analysis was conducted
**Analytics and assumptions**	17	Describe any methods for analysing or statistically transforming data, any extrapolation methods, and approaches for validating any model used.	See the 'Incremental analyses' section of the Methods

Topic	No.	Item	Location where item is reported
**Characterising heterogeneity**	18	Describe any methods used for estimating how the results of the study vary for subgroups.	See the fifth sensitivity analysis (SA per protocol) in 'Appendix 3: further sensitivity analyses' of the Supplemental Material (Appendices)
**Characterising distributional effects**	19	Describe how impacts are distributed across different individuals or adjustments made to reflect priority populations.	Not reported (Not conducted) – the HEAP was developed before this updated CHEERS checklist was available
**Characterising uncertainty**	20	Describe methods to characterise any sources of uncertainty in the analysis.	See the 'Decision uncertainty' and 'Sensitivity analyses' section of the Methods
**Approach to engagement with patients and others affected by the study**	21	Describe any approaches to engage patients or service recipients, the general public, communities, or stakeholders (such as clinicians or payers) in the design of the study.	The patient self-report questionnaire was developed in consultation with non-trial patients. See the 'Measuring Costs' section of the Methods
**Results**			
**Study parameters**	22	Report all analytic inputs (such as values, ranges, references) including uncertainty or distributional assumptions.	Not applicable, a within trial cost effectiveness analysis was conducted
**Summary of main results**	23	Report the mean values for the main categories of costs and outcomes of interest and summarise them in the most appropriate overall measure.	See Tables 2 (Costs) and 3 (Outcomes), these are referred to in the 'Costs' and 'Outcomes' section of the Results.
**Effect of uncertainty**	24	Describe how uncertainty about analytic judgments, inputs, or projections affect findings. Report the effect of choice of discount rate and time horizon, if applicable.	Sensitivity analyses are reported in Table 5 and Supplemental Table A1. Within these Tables the estimated probability values for the cost-effectiveness acceptability curve (CEAC) are also reported for base-case and sensitivity analyses.
**Effect of engagement with patients and others affected by the study**	25	Report on any difference patient/service recipient, general public, community, or stakeholder involvement made to the approach or findings of the study	The patient self-report questionnaire was developed in consultation with non-trial patients. See the 'Measuring Costs' section of the Methods
**Discussion**			
**Study findings, limitations, generalisability, and current knowledge**	26	Report key findings, limitations, ethical or equity considerations not captured, and how these could affect patients, policy, or practice.	See the Discussion

Topic	No.	Item	Location where item is reported
**Other relevant information**			
**Source of funding**	27	Describe how the study was funded and any role of the funder in the identification, design, conduct, and reporting of the analysis	See the 'Conflicts of Interest and Source of Funding' section
**Conflicts of interest**	28	Report authors conflicts of interest according to journal or International Committee of Medical Journal Editors requirements.	See the 'Conflicts of Interest and Source of Funding' section

From: Husereau D, Drummond M, Augustovski F, et al. Consolidated Health Economic Evaluation Reporting Standards 2022 (CHEERS 2022) Explanation and Elaboration: A Report of the ISPOR CHEERS II Good Practices Task Force. Value Health 2022;25. doi:10.1016/j.jval.2021.10.008

For peer review only

For peer review only - http://bmjopen.bmj.com/site/about/guidelines.xhtml

BMJ Open

Cost-effectiveness of craniotomy versus decompressive craniectomy, for UK patients with traumatic acute subdural hematoma

Journal:	*BMJ Open*
Manuscript ID	bmjopen-2024-085084.R1
Article Type:	Original research
Date Submitted by the Author:	18-Apr-2024

Complete List of Authors:	Pyne, Sarah; University of East Anglia, Norwich Medical School<br>Barton, Garry ; University of East Anglia Faculty of Medicine and Health Sciences, Norwich Medical School<br>Turner, David; University of East Anglia, Norwich Medical School<br>Mee, Harry; University of Cambridge, Department of Clinical Neurosciences<br>Gregson, Barbara; Newcastle University, Neurosurgical Trials Group, Wolfson Research Centre<br>Kolias, Angelos; Cambridge University, Division of Neurosurgery<br>Turner, Carole; University of Cambridge, Department of Clinical Neurosciences; Addenbrooke's Hospital, Division of Neurosurgery<br>Adams, Hadie; University of Cambridge, Department of Clinical Neurosciences; Addenbrooke's Hospital, Division of Neurosurgery<br>Mohan, Midhun; University of Cambridge, Department of Clinical Neurosciences; Addenbrooke's Hospital, Division of Neurosurgery<br>Uff, Christopher; The Royal London Hospital<br>Hasan, Shumaila; The Royal London Hospital<br>Wilson, Mark; St Mary's Hospital, Department of Neurosurgery<br>Bulters, Diederik ; University Hospital Southampton NHS Foundation Trust, Wessex Neurological Centre<br>Zolnourian, Ardalan; University Hospital Southampton NHS Foundation Trust<br>McMahon, Catherine; The Walton Centre NHS Foundation Trust, Department of Neurosurgery<br>Stovell, Matthew G; University of Cambridge, Department of Clinical Neurosciences<br>Al-Tamimi, YAHIA; Sheffield Teaching Hospitals NHS Foundation Trust, Department of Neurosurgery; Sheffield Teaching Hospitals NHS Foundation Trust, Academic Directorate of Neurosciences<br>Thomson, Simon; Leeds General Infirmary, Department of Neurosurgery<br>Viaroli, Edoardo; Addenbrooke's Hospital, Division of Neurosurgery; University of Cambridge, Department of Clinical Neurosciences<br>Belli, Antonio; University Hospitals Birmingham NHS Foundation Trust, Department of Neurosurgery<br>King, Andrew; Salford Royal Hospital Manchester Centre for Clinical Neurosciences, Department of Neurosurgery<br>Helmy, Adel E; Addenbrooke's Hospital, Division of Neurosurgery; University of Cambridge, Department of Clinical Neurosciences<br>Timofeev, Ivan; University of Cambridge, Department of Clinical

	Neurosciences; Addenbrooke's Hospital, Division of Neurosurgery<br>Menon, David; University of Cambridge, Division of Anaesthesia<br>Hutchinson, Peter; University of Cambridge, Department of Clinical Neurosciences; Addenbrooke's Hospital, Division of Neurosurgery<br>RESCUE-ASDH, trial collaborators; University of Cambridge
**Primary Subject Heading**:	Health economics
Secondary Subject Heading:	Neurology, Surgery
Keywords:	HEALTH ECONOMICS, Health economics < HEALTH SERVICES ADMINISTRATION & MANAGEMENT, NEUROSURGERY, Randomized Controlled Trial, Neurosurgery < SURGERY, Brain Injuries

SCHOLARONE™
Manuscripts

I, the Submitting Author has the right to grant and does grant on behalf of all authors of the Work (as defined in the below author licence), an exclusive licence and/or a non-exclusive licence for contributions from authors who are: i) UK Crown employees; ii) where BMJ has agreed a CC-BY licence shall apply, and/or iii) in accordance with the terms applicable for US Federal Government officers or employees acting as part of their official duties; on a worldwide, perpetual, irrevocable, royalty-free basis to BMJ Publishing Group Ltd ("BMJ") its licensees and where the relevant Journal is co-owned by BMJ to the co-owners of the Journal, to publish the Work in this journal and any other BMJ products and to exploit all rights, as set out in our licence.

The Submitting Author accepts and understands that any supply made under these terms is made by BMJ to the Submitting Author unless you are acting as an employee on behalf of your employer or a postgraduate student of an affiliated institution which is paying any applicable article publishing charge ("APC") for Open Access articles. Where the Submitting Author wishes to make the Work available on an Open Access basis (and intends to pay the relevant APC), the terms of reuse of such Open Access shall be governed by a Creative Commons licence – details of these licences and which Creative Commons licence will apply to this Work are set out in our licence referred to above.

Other than as permitted in any relevant BMJ Author's Self Archiving Policies, I confirm this Work has not been accepted for publication elsewhere, is not being considered for publication elsewhere and does not duplicate material already published. I confirm all authors consent to publication of this Work and authorise the granting of this licence.

For peer review only

Cost-effectiveness of craniotomy versus decompressive craniectomy, for UK patients with traumatic acute subdural hematoma

Authors:

Sarah C Pyne, Garry R Barton*, David A Turner, Harry Mee, Barbara Gregson, Angelos G Kolias, Carole Turner, Hadie Adams, Midhun Mohan, Christopher Uff, Shumaila Hasan, Mark H Wilson, Diederik Bulters, Ardalan Zolnourian, Catherine J McMahon, Matthew G Stovell, Yahia Z Al-Tamimi, Simon Thomson, Edoardo Viaroli, Antonio Belli, Andrew T King, Adel E Helmy, Ivan S Timofeev, David K Menon, Peter J Hutchinson, RESCUE-ASDH trial collaborators†

*Corresponding author: Professor Garry Barton, Health Economics Group, Norwich Medical School, University of East Anglia, Norwich NR4 7TJ. Email: g.barton@uea.ac.uk

†Author affiliations, along with a complete list of the investigators and members of the trial oversight committees is provided in the Supplemental Material (Appendix 1).

For peer review only

ABSTRACT

Objective: To estimate the cost-effectiveness of craniotomy, compared with decompressive craniectomy (DC) in UK patients undergoing evacuation of acute subdural haematoma (ASDH).

Design: Economic evaluation undertaken using health resource use and outcome data from the 12-month multi-centre, pragmatic, parallel-group, randomised, RESCUE-ASDH trial.

Setting: UK secondary care.

Participants: 248 UK patients undergoing surgery for traumatic ASDH randomised to craniotomy (N=126) or DC (N=122).

Interventions: Surgical evacuation via craniotomy (bone flap replaced) or decompressive craniectomy (bone flap left out with a view to replace later cranioplasty surgery).

Main outcome measures: In the base-case analysis costs were estimated from an NHS and personal social services perspective. Outcomes were assessed via the Quality-Adjusted Life Years (QALY) derived from the EQ-5D-5L questionnaire (cost-utility analysis) and the Extended Glasgow Outcome Scale (GOSE) (cost-effectiveness analysis). Multiple imputation and regression analyses were conducted to estimate the mean incremental cost and effect of craniotomy compared to DC. The most cost-effective option was selected, irrespective of the level of statistical significance as is argued by economists.

Results: In the cost-utility analysis the mean incremental cost of craniotomy compared to DC was estimated to be -£5,520 (95% confidence interval (CI) -£18,060 to £7,020) with a mean QALY gain of 0.093 (95% CI 0.029 to 0.156). In the cost-effectiveness analysis, the mean incremental cost was estimated to be -£4,536 (95% CI -£17,374 to £8,301) with an odds ratio of 1.682 (95% CI 0.995 to 2.842) for a favourable outcome on the GOSE.

Conclusions: In a UK population with traumatic ASDH craniotomy was estimated to be cost-effective compared to decompressive craniectomy: craniotomy was estimated to have a lower mean cost, higher mean QALY gain and higher probability of a more favourable outcome on the GOSE (though not all estimated differences between the two approaches were statistically significant).

For peer review only - http://bmjopen.bmj.com/site/about/guidelines.xhtml

For peer review only

Trial registration and ethics: Ethical approval for the trial was obtained from the North West – Haydock Research Ethics Committee in the United Kingdom on 17th July 2014 (14/NW/1076). The trial was registered prospectively: ISRCTN87370545.

STRENGTHS AND LIMITATIONS OF THIS STUDY

- This study is based on individual patient-level data from a large, pragmatic, multi-centre randomised trial.
- It is both the first randomised trial and the first economic evaluation to compare craniotomy to decompressive craniectomy.
- Multiple imputation was undertaken to account for missing data.
- For ethical reasons, baseline EQ-5D-5L scores were taken at discharge from neurosurgical unit (NSU), rather than at randomisation.
- A number of sensitivity analyses were undertaken to assess the robustness of conclusions to different assumptions in relation to these and other aspects.

For peer review only

BACKGROUND

In the UK an estimated 1.3 million people live with a traumatic brain injury related disability and the annual societal cost has been estimated to be £15 billion (2015 cost levels).[1] Acute subdural hematoma (ASDH) is a common consequence[2] where craniotomy and decompressive craniectomy (DC) are the two mainstay treatments for surgical evacuation of the hematoma.[3] Both involve the removal of a piece of skull (bone-flap) to evacuate the hematoma. With craniotomy the bone-flap is replaced, whereas with DC it is not. DC may help alleviate brain swelling and is undertaken with the view to a further operation being performed to rebuild the skull (cranioplasty). Craniotomy has the advantage that a patient will not need a later operation to rebuild the skull, but it may fail to control brain swelling in some patients. A systematic review found few studies comparing the two procedures, none of which were randomised, with contrasting evidence as to which was superior.[3]

Given this uncertainty as to whether craniotomy or DC is the more effective treatment for patients with ASDH, the choice of treatment is generally left to the discretion of the surgeon.[3] However, guidance/recommendations for the provision of different treatment options are now often based on estimated levels of cost-effectiveness.[4] Moreover, levels of cost-effectiveness may differ between these two surgical procedures as, for example, DC often requires cranial reconstruction by means of cranioplasty, which has additional costs and a

For peer review only - http://bmjopen.bmj.com/site/about/guidelines.xhtml

significant complication profile,[5] but may better alleviate brain swelling, translating into quality-of-life benefits.[5] Thus, here we report an economic evaluation[6] that was conducted alongside the RESCUE-ASDH (Randomized Evaluation of Surgery with Craniectomy for Patients Undergoing Evacuation of Acute Subdural Hematoma) trial,[5] to compare the cost-effectiveness of craniotomy versus DC for UK patients with traumatic ASDH.

METHODS

Participants

The RESCUE-ASDH trial[5] is a multicentre, international, pragmatic, parallel-group, randomised trial that compared craniotomy with DC. Patients were eligible if they were ≥16 years, had an ASDH on CT scan, and the admitting surgeon felt that the hematoma needed evacuating either by craniotomy or DC. The economic evaluation was nested within the RESCUE-ASDH trial and based on UK participants only.

Treatment and randomisation

Enrolled patients had their ASDH evacuated in the operating room under general anaesthesia. The bone-flap was raised, the dura opened, and the hematoma evacuated, after which patients were randomly assigned to receive either craniotomy (bone-flap restored before skin closure) or DC (bone-flap removed prior to skin closure with a view to being restored later). Patients were only randomised if either treatment was feasible, those patients whose brain was too swollen to allow replacement of the bone flap were not randomised. These patients would have the bone flap left out and were not included in the ITT analysis presented within this paper. As a pragmatic study, management of patients pre-, intra-, and post-operatively was undertaken according to each centre's standard of care.

Blocked randomisation (block size 4) with 1:1 ratio was used, with allocation stratified by geographical region, age group, severity of injury and CT findings.[5] Patients randomised to craniotomy could have a DC at a later stage if their condition deteriorated and at the discretion of the treating clinician. It was not possible to blind patients, relatives and treating clinicians but the primary outcome (see below) was adjudicated centrally by blinded investigators.

Measuring costs

Costs were estimated from a UK NHS and Personal Social Services (PSS) perspective.[7] Resource use data was collected via two methods: hospital-recorded data and a patient self-report (12-month follow-up) questionnaire (PSRQ). Both methods of data collection were developed in consultation with hospital staff/patients and focussed on big cost drivers/resources that were expected to differ between arms.[8] All resource use items that

For peer review only - http://bmjopen.bmj.com/site/about/guidelines.xhtml

were costed (see below) were estimated in £ Sterling for the 2018/19 financial year, resource use items undertaken for research purposes were not costed.

The hospital-recorded data included the following resource use items: details of the intervention (craniotomy or DC) including length of operation and graft details; time spent in the intensive care unit (ICU) and neurosurgical unit (NSU) during initial (index) admission; cranioplasties and shunt placements (these could be received as part of the index admission and/or after discharge from the NSU); any further neurosurgical procedures received during index admission.

The 12-month follow-up PSRQ could be completed by a relative/friend/carer if the participant was unable to complete it and referred only to the time since discharge from NSU. Information requested included the following resource use items: overnight stays in a hospital or other healthcare facility (length of stay, ward type, any associated skull/brain operation); healthcare professional visits (professional seen, frequency and most common location); head/brain scans (MRI, CT or 'other'); time in a care home; help received from a family member/friend or carer.

After assigning unit costs to the resource use items (see Supplemental Table S1 for unit costs), the costs associated with both the hospital-recorded data and PSRQ resource use items, excluding wider societal costs (care home and help/carer costs), were summed to estimate the total NHS and PSS cost per participant. For each group the mean total costs were estimated over the 12-month follow-up period, along with the associated p-value for the mean cost difference between groups. An exception to the above was that, to avoid double-counting, patient self-reported post-discharge overnight stays with an associated skull/brain operation would not be costed if the total reported number was less than the total reported number of hospital-recorded post-discharge cranioplasty and shunt procedures (including revisions). In line with previous work,[9] the higher of the two values was considered the most accurate. It should also be noted that patients who were known to have died post-discharge (mortality is collected as part of the primary outcome, see below) were not sent the PSRQ. As such, post-discharge costs for these participants would have been treated as missing and estimated via imputation (see below for details of the imputation methods, where time post-discharge was included in the MI model). In contrast, cost data for participants who died before discharge from their index admission would not have been considered missing as hospital-recorded data would still have been available for such participants (post-discharge costs were set as equal to zero for such participants).

For peer review only - http://bmjopen.bmj.com/site/about/guidelines.xhtml

Measuring outcomes

To estimate health-related quality-of-life, and conduct a cost-utility analysis,[6] in line with UK NICE guidance,[4,10] the five-dimension EuroQoL five-level questionnaire (EQ-5D-5L)[11] was combined with mortality data to estimate quality-adjusted life-year (QALY)[6] scores. Participants completed the EQ-5D-5L at discharge from NSU (assumed baseline score), 6- and 12-months follow-up (if discharged from NSU by these time points). As recommended at the time of analysis,[12] the crosswalk mapping function[13] was used to convert responses into utility scores (range: -0.594 (worse than death) to 1 (full health)). Participants who died were assigned a utility score of 0 on their date of death (death was collected as part of the hospital-recorded data as it was required for the primary outcome, see below). Utility values were used to estimate QALYs over 12 months, based on the total area under the curve method and linear interpolation.[14]

For ease of interpretation, as is convention,[15] the trial primary outcome measure, the (ordinal) extended Glasgow Outcome Scale (GOSE),[16–19] at 12 months, was converted into a binary scale using a fixed dichotomy analysis (i.e. favourable vs. unfavourable)[5] to enable a cost-effectiveness analysis[6] to be undertaken. Favourable outcomes were defined as upper severe disability or better; while unfavourable outcomes included death, vegetative state and lower severe disability. A sliding dichotomy analysis[5] was also undertaken and is described in the Supplemental Material (Appendix 2).

Missing data

Missing data is common in randomised trials and can lead to bias and lack of precision.[20] As recommended, patterns of missing data were examined to explore the mechanism of missingness.[20] Accordingly, multiple imputation (MI) with chained equations (MICE) under MAR (missing at random) was used to impute missing data, by treatment group. The “mi impute chained” command (Stata 17.0 [StataCorp LP, College Station, TX]) was used to create 30 data sets (based on recommendations in relation to the level of missing data[20]) that were then pooled using Rubin’s rules.[21]

For costs missing data was imputed at the level of total costs for: index admission, cranioplasty and shunt, and post-discharge. For outcomes missing data was imputed for utility scores (EQ-5D-5L) at baseline, 6 and 12 months, Glasgow Coma Scale (GCS) score[22,23] at baseline and GOSE score at 12 months. In addition to these costs and outcomes, the MI model also included age (years), sex and time post-discharge (the number of days from discharge to the 12-month point or death).

For peer review only - http://bmjopen.bmj.com/site/about/guidelines.xhtml

Incremental analyses

For both the cost-utility and cost-effectiveness analyses, a 12-month within-trial, intention-to-treat (ITT) approach was adopted. In this base-case analysis, patients were analysed according to the treatment to which they were randomised, regardless of treatment received. No discounting was undertaken.

For the cost-utility analysis, to estimate the mean incremental cost and incremental effect (QALY gain) associated with craniotomy compared with DC, seemingly unrelated regression analysis was undertaken.[24] Regressions included those baseline variables expected to be predictive of total costs and outcomes: age (years), sex and baseline utility score. Assuming dominance,[6] where an intervention was both more costly and less effective, did not occur the incremental cost-effectiveness ratio (ICER = mean incremental cost / mean incremental QALY),[10] for craniotomy versus DC, would be estimated.[6] In the UK, NICE refers to a cost-effectiveness threshold of £20,000 to £30,000 per QALY.[10] As such, if craniotomy had an ICER below this level, this would suggest it is cost-effective, compared with DC. It should be noted that economists have argued that decisions about treatment adoption should be made based on mean estimates, irrespective of whether such differences are statistically significant.[25] Therefore, the treatment option which is estimated to be most cost-effective should be provided.[26] This approach is consistent with the objective of maximizing benefits from a given budget.

For the cost-effectiveness analysis, in terms of the incremental effect, the outcome (based on the GOSE) had a binary scale (favourable/unfavourable) and logistic regression[27] was undertaken to estimate the odds ratio (95% CI) of a favourable outcome for craniotomy compared with DC. Separately, the mean incremental cost associated with craniotomy compared with DC was estimated using linear regression. Both regressions included variables age (years) and sex, which were expected to be predictive of total costs and GOSE outcomes. Together, in the absence of dominance, the incremental cost and incremental effect would enable the ICER to be estimated in terms of the cost per percentage increase in the odds of a favourable outcome.

Decision uncertainty

To estimate the level of uncertainty associated with the decision regarding cost-effectiveness estimates of the mean coefficients and covariance matrix were combined, as described in Faria et al.[20], to calculate the probability of craniotomy being cost-effective, compared with DC, at the threshold of £20,000/QALY on the cost-effectiveness acceptability curve (CEAC).[26] The CEAC was only estimated in relation to the cost-utility analysis.

For peer review only - http://bmjopen.bmj.com/site/about/guidelines.xhtml

Sensitivity analyses

The above analysis constituted the base-case analysis[6] and was carried out in accordance with a pre-specified health economic analysis plan (HEAP) (see: https://www.rescueasdh.org/trial-documents). To assess the robustness of conclusions, sensitivity analyses (SA) were undertaken.[6] To analyse the data from a wider cost perspective the care home and carer costs (which were excluded from the base-case analysis) were added to the total NHS and PSS costs (SA wider cost perspective). A further sensitivity analysis (for the cost-utility analysis only) tested the use of the EQ-5D-5L score at discharge from NSU as the baseline for QALY calculations. As any benefits could already have been partially/wholly achieved by discharge, QALY scores were re-estimated with the assumption that, given the grave nature of the condition and following expert advice, participants had the lowest possible EQ-5D-5L score at baseline (date of index surgery): -0.594 (SA lowest EQ-5D-5L baseline score). Four further sensitivity analyses (including a per protocol analysis) were conducted and are presented in the Supplemental Material (Appendix 3). “SA wider cost perspective” deviated from the HEAP, for reasons explained in the Supplemental Material (Appendix 4)

Patient and public involvement

The aforementioned patient self-report questionnaire was developed in consultation with non-trial patients.

RESULTS

Participants

Between September 2014 and April 2019 248 UK patients were recruited, 126 in the craniotomy arm and 122 in the DC arm. Compared with the 450 patients recruited to the full (international) trial (the baseline characteristics of which are summarised in Table 1 of Hutchinson et al. [5]), these UK patients are slightly older (3.5 years on average) and more likely to be on antithrombotic medication (Table 1).

Levels of missing data were slightly lower in the craniotomy group compared with the DC group for cost variables and outcome variables (except at baseline) (Supplemental Table S2).

Costs

Levels of resource use by intervention arm are summarised in Table 2, under three main categories: (i) Hospital-recorded index-admission; (ii) Hospital-recorded cranioplasties and shunts; (iii) Patient-reported (PSRQ) post-discharge.

For peer review only - http://bmjopen.bmj.com/site/about/guidelines.xhtml

The hospital-recorded index-admission data shows that, length of stay in ICU and NSU was slightly lower in the craniotomy group compared with the DC group, but not significantly so. Only small numbers of other neurosurgical operations were reported. With regard to cranioplasties and shunts (index admission and post-discharge), as expected, more patients in the DC group had cranioplasties than in the craniotomy group (DC is pre-requisite to a cranioplasty). There were, however, patients who were randomised to craniotomy that went on to have a DC, 21 of which had a cranioplasty in the 12-month follow-up period. Most cranioplasties used a synthetic material. Shunts were uncommon and occurred at a similar frequency between the groups. In terms of the patient-reported (PSRQ) post-discharge resource use, there were no significant differences between the groups for any of the parameters measured.

Mean cost estimates are summarised in Table 3 and divided into the same three main categories. As expected, given the procedure complexity and recovery time, total NHS and PSS costs are high in both groups. High index admission costs particularly accounted for this, largely due to the high cost of ICU stays, along with post-discharge costs, largely due to the high cost of overnight stays on a rehabilitation unit. There were however few significant differences between groups, the only notable one being the cost of cranioplasty procedures which, for aforementioned reasons, was significantly higher in the DC group. As the number of post-discharge hospital-recorded cranioplasty/shunt procedures exceeded patient-reported over-night stays with an associated skull/brain operation (Table 2), the latter has not been costed.

Outcomes

Outcomes are summarised in Table 4. Follow-up mean EQ-5D-5L scores were higher in the craniotomy group compared with the DC group, significantly so at 12 months. Furthermore, the change (increase) in EQ-5D-5L score from baseline was significantly higher at both 6 and 12 months in the craniotomy group compared with the DC group. There was no significant difference between groups for the total QALY score, based on available data.

At 12 months the percentage of favourable GOSE scores was higher, but not significantly, in the craniotomy group compared with the DC group.

Analyses

Cost-utility analysis

For the base-case (based on ITT/MI), the mean difference in cost for the craniotomy group compared with the DC group was -£5,520 (95% CI -£18,060 to £7,020) with a mean QALY difference of 0.093 (95% CI 0.029 to 0.156) (Table 5). Craniotomy therefore dominated DC; it was estimated to be associated with both lower costs and more benefit.

For peer review only - http://bmjopen.bmj.com/site/about/guidelines.xhtml

Cost-effectiveness analysis

For the craniotomy group compared with the DC group, the mean difference in cost was -£4,536 (95% CI -£17,374 to £8,301) with an odds ratio of favourable outcome on the GOSE score of 1.682 (95% CI 0.995 to 2.842) (Table 5). Again, craniotomy therefore dominated DC.

Decision uncertainty

The base-case probability that craniotomy was cost-effective compared with DC, at a threshold of £20,000/QALY, was 87% (Table 5). This indicates a high degree of certainty associated with the cost-utility analysis decision that craniotomy compared with DC is cost-effective at that threshold.

Sensitivity analyses

In the sensitivity analyses, from a wider cost perspective and using the lowest EQ-5D-5L baseline score (for the cost-utility analysis only), craniotomy was again found to dominate DC (see Table 5). Results of further sensitivity analyses, all of which are consistent with the base-case results, are presented in Supplemental Table A1 (Supplemental Material, Appendix 3).

DISCUSSION

Main findings

In this UK population of patients with traumatic ASDH that warrants surgical evacuation, based on the results of the cost-utility and cost-effectiveness analyses, craniotomy dominated DC as it was estimated to have a lower mean cost, a higher mean QALY gain / higher probability of a more favourable outcome on the GOSE. Craniotomy was therefore estimated to be cost-effective, on the basis that the associated level of significance is considered to be irrelevant.[25,26] In the cost-utility analysis (QALY outcome), there was only an estimated 13% probability (at a threshold of £20,000/QALY) of making the wrong decision by choosing craniotomy. The results of the sensitivity analyses are in keeping with this result.

Within this study it is important to highlight that costs were estimated from the viewpoint of the UK NHS and PSS and that associated resource use and outcome data was based only on participants from UK sites. As, for example, unit costs may differ outside the UK it is important to note that it is unclear whether these results are generalisable to sites outside the UK. Further associated research may therefore be warranted in relation to this and that ≥20% of patients who were randomised to craniotomy went on to have a DC (as an ITT

For peer review only - http://bmjopen.bmj.com/site/about/guidelines.xhtml

approach was adopted these patients were included in the craniotomy arm in the base-case analysis).

Strengths and limitations

Regarding health-related quality-of-life, QALY scores (EQ-5D-5L recorded at all time points) were available for 53% of participants only and the amount of missing data was greater at discharge than at 6 and 12 months (Supplemental Table S2). Some missing EQ-5D-5L baseline (NSU discharge) data may be due to participants being discharged at short notice or at the weekend when a research nurse was not available. As some patients had not yet been discharged from hospital by 6 months, this may explain the higher rates of missing data at this time point compared with 12 months. Post-discharge costs (based on patient self-report data) were also missing for 27.4% of patients at 12 months (Supplemental Table S2). Such missing data is a limitation, but we did impute missing data and take an ITT approach, which meant that all patients were still included in the analysis.

A further limitation is that, for ethical reasons, baseline EQ-5D-5L scores were taken at discharge from NSU, rather than at randomisation. Therefore, any benefits could be underestimated by assuming this score is the baseline score. To test the potential impact of this, a sensitivity analysis (SA lowest EQ-5D-5L baseline score) assumed the baseline EQ-5D-5L score to be that of worst possible health state (-0.594). The results differ little from the base-case (Table 5) with craniotomy still dominating DC. It should also be noted that, in the cost-effectiveness analysis, as the cost and outcome regressions are performed separately any correlation between the cost and outcome variables would not be accounted for. A final limitation is that the 12 month follow-up period may not be sufficient to capture all expected cranioplasties. For example, of those randomised to DC (122), only 62 had received a cranioplasty within the 12 month follow-up period. As such, further cranioplasties (aside from those who were randomised to but did not receive DC (8/122) and those who had died (31/122)) could take place beyond the 12 month period. Though this is a limitation, the inclusion of such costs would only be expected to increase the long term incremental cost of DC and therefore not change the conclusion that craniotomy dominated DC.

Comparisons with other studies

We are not aware of any previous economic evaluations that have specifically compared craniotomy with DC for patients with ASDH. Previous economic evaluations of DC have been undertaken,[28–32] but these have had different comparators, and used a variety of different populations/methods (most developed a decision analytical model to estimate costs and benefits,[30–32] and the two papers[28,29] that used actual patient data were not based on randomised data and were of a smaller sample size than used here, with different cost

For peer review only - http://bmjopen.bmj.com/site/about/guidelines.xhtml

perspectives and timeframes. Thus, it is difficult to make direct comparisons to our study, and the use of different methods may explain why there were differences in the results as to whether DC was estimated to be cost-effective or not.[28–32]

Implications

In a UK population of patients with traumatic ASDH, craniotomy was estimated to have a lower mean cost, a higher mean QALY gain and a higher probability of a more favourable outcome on the GOSE, dominating DC. Based on the QALY, there was a high probability that craniotomy, compared with DC, was cost-effective (at a threshold of £20,000/QALY). When sensitivity analyses were conducted, the main conclusion (that craniotomy was therefore estimated to be cost-effective) remained unchanged. Consequently, the health economic analysis supports the recommendation, based on the primary outcome,[5] that a craniotomy should be undertaken, rather than a DC, if it is operatively feasible to replace the bone-flap.

For peer review only

For peer review only - http://bmjopen.bmj.com/site/about/guidelines.xhtml

Contributors:

GRB, DAT, HM, BG, AGK, CT, HA, MM, CJM, AB, ATK, DKM and PJH contributed to the conception/design of the work. SCP, GRB, DAT, HM, BG, AGK, CT, HA, MM, CU, SH, MW, DB, AZ, CJM, MGS, YZA, ST, EV, AEH, IST, DKM, PJH contributed to the acquisition of data. SCP, GRB and DT conducted the analysis. All authors contributed to the interpretation of data/drafting of the paper (led by SCP and GRB) and approved the final manuscript.

Competing interests: No support from any organisation other than the National Institute for Health and Care Research was received for the submitted work. Barbara Gregson has received consulting fees from Cambridge University Hospitals NHS Foundation Trust. Angelos Kolias is supported by a Senior Lectureship at the School of Clinical Medicine, University of Cambridge, the Wellcome Trust, and the Royal College of Surgeons of England. Mark Wilson has received support for attending meetings and/or travel for presentations with the Wilderness Medical Society and Royal College of Surgeons of Edinburgh, is a member of the Trauma Clinical Reference group for the NHS, meetings secretary for the Society of British Neurosurgeons and a non-salaried medical director of GoodSAM. Peter Hutchinson is supported by a Research professorship and Senior Investigator award from the NIHR, the NIHR Cambridge Biomedical Research Centre, and the Royal College of Surgeons of England.

Funding statement: This project was supported by the Health Technology Assessment (HTA) Programme (project number 12/35/57) and will be published in full in the HTA journal at https://fundingawards.nihr.ac.uk/award/12/35/57; The RESCUE-ASDH trial is an “embedded study” linked with the CENTER-TBI project (https://www.center-tbi.eu/) of the European Brain Injury Consortium. CENTER-TBI was a large-scale collaborative project, supported by the FP7 Program of the European Union (grant number 602150); RESCUE-ASDH ISRCTN Registry number, ISRCTN87370545.

Study protocol is available at https://fundingawards.nihr.ac.uk/award/12/35/57

The views expressed are those of the authors and not necessarily those of the NHS, the National Institute for Health and Care Research (NIHR), or the Department of Health and Social Care.

We thank the patients who participated in the RESCUE-ASDH trial, their families, and all the collaborating clinicians and research staff, and we thank the staff of the Cambridge Clinical Trials Unit for their support.

Data availability statement: Reasonable requests to make relevant anonymised participant level data available will be considered by the trial team. The trial protocol[15] and a pre-

For peer review only - http://bmjopen.bmj.com/site/about/guidelines.xhtml

specified health economic analysis plan (HEAP) (see: https://www.rescueasdh.org/trial-documents) are also available.

REFERENCES

1. Parsonage M. *Traumatic brain injury and offending. An economic analysis*. 2016.
2. Wilberger JE, Harris M, Diamond DL. Acute subdural hematoma: morbidity, mortality, and operative timing. *J Neurosurg* 1991; 74: 212–8.
3. Phan K, Moore JM, Griessenauer C, et al. Craniotomy versus decompressive craniectomy for acute subdural hematoma: systematic review and meta-analysis. *World Neurosurg* 2017; 101: 677-685.e2.
4. NICE. *NICE health technology evaluations: the manual (PMG 36)*, https://www.nice.org.uk/process/pmg36/chapter/introduction-to-health-technology-evaluation (2022).
5. Hutchinson PJ, Adams H, Mohan M, et al. Decompressive Craniectomy versus Craniotomy for Acute Subdural Hematoma. *NEJM* 2023; 388: 2219–2229.
6. Drummond M, Sculpher M, Claxton K, et al. *Methods for the economic evaluation of health care programmes*. 4th ed. Oxford, UK: Oxford University Press, 2015.
7. NICE. *Guide to the methods of technology appraisal 2013*. 2013. Epub ahead of print 2013. DOI: 10.2165/00019053-200826090-00002.
8. Ramsey SD, Willke RJ, Glick H, et al. Cost-effectiveness analysis alongside clinical trials II - An ISPOR good research practices task force report. *Value in Health* 2015; 18: 161–172.
9. Byford S, Leese M, Knapp M, et al. Comparison of alternative methods of collection of service use data for the economic evaluation health care interventions. *Health Econ* 2007; 16: 531–536.
10. NICE. *Guide to the methods of technology appraisal 2013*. National Institute for Health and Care Excellence, 2013.
11. Herdman M, Gudex C, Lloyd A, et al. Development and preliminary testing of the new five-level version of EQ-5D (EQ-5D-5L). *Qual Life Res* 2011; 20: 1727–36.
12. NICE. *Position statement on use of the EQ-5D-5L value set for England (updated October 2019)*. National Institute for Health and Care Excellence, 2019.

For peer review only - http://bmjopen.bmj.com/site/about/guidelines.xhtml

13. Van Hout B, Janssen MF, Feng YS, et al. Interim scoring for the EQ-5D-5L: Mapping the EQ-5D-5L to EQ-5D-3L value sets. *Value in Health* 2012; 15: 708–715.

14. Manca A, Hawkins N, Sculpher MJ. Estimating mean QALYs in trial-based cost-effectiveness analysis: The importance of controlling for baseline utility. *Health Econ* 2005; 14: 487–496.

15. Hutchinson P. *Randomised Evaluation of Surgery with Craniectomy for patients Undergoing Evacuation of Acute Subdural Haematoma (RESCUE-ASDH) trial protocol version: 3.0*, https://www.fundingawards.nihr.ac.uk/award/12/35/57 (2018, accessed 12 April 2024).

16. Jennett B, Bond M. Assessment of outcome after severe brain damage. *The Lancet* 1975; 1: 480–484.

17. Teasdale GM, Pettigrew LEL, Wilson JTL, et al. Analyzing outcome of treatment of severe head injury: a review and update on advancing the use of the Glasgow Outcome Scale. *J Neurotrauma* 1998; 15: 587–597.

18. Wilson JTL, Slieker FJA, Legrand V, et al. Observer variation in the assessment of outcome in traumatic brain injury: experience from a multicenter, international randomized clinical trial. *Neurosurgery* 2007; 61: 123–128.

19. Wilson JTL, Pettigrew LEL, Teasdale GM. Structured interviews for the Glasgow Outcome Scale and the extended Glasgow Outcome Scale: guidelines for their use. *J Neurotrauma* 1998; 15: 573–580.

20. Faria R, Gomes M, Epstein D, et al. A Guide to Handling Missing Data in Cost-Effectiveness Analysis Conducted Within Randomised Controlled Trials. *Pharmacoeconomics* 2014; 32: 1157–1170.

21. Little R, Rubin D. *Statistical analysis with missing data*. 2nd ed. Hoboken, New Jersey: Wiley, 2002.

22. Teasdale G, Jennett B. Assessment of coma and impaired consciousness. A practical scale. *Lancet* 1974; 2: 81–4.

23. Teasdale G, Murray G, Parker L, et al. Adding up the Glasgow Coma Score. *Acta Neurochir Suppl (Wien)* 1979; 28: 13–6.

24. Willan AR, Briggs AH, Hoch JS. Regression methods for covariate adjustment and subgroup analysis for non-censored cost-effectiveness data. *Health Econ* 2004; 13: 461–475.

For peer review only - http://bmjopen.bmj.com/site/about/guidelines.xhtml

25. Claxton K. The irrelevance of inference: A decision-making approach to the stochastic evaluation of health care technologies. *J Health Econ* 1999; 18: 341–364.

26. Fenwick E, O'Brien BJ, Briggs AH. Cost-effectiveness acceptability curves-facts, fallacies and frequently asked questions. *Health Econ* 2004; 13: 405–415.

27. Hosmer Jr DW, Lemeshow S, Sturdivant RX. *Applied logistic regression*. John Wiley & Sons, 2013.

28. Malmivaara K, Kivisaari R, Hernesniemi J, et al. Cost-effectiveness of decompressive craniectomy in traumatic brain injuries. *Eur J Neurol* 2011; 18: 656–662.

29. Ho KM, Honeybul S, Lind CRP, et al. Cost-effectiveness of decompressive craniectomy as a lifesaving rescue procedure for patients with severe traumatic brain injury. *J Trauma* 2011; 71: 1637–1644.

30. Whitmore RG, Thawani JP, Grady MS, et al. Is aggressive treatment of traumatic brain injury cost-effective? *J Neurosurg* 2012; 116: 1106–1113.

31. Alali AS, Naimark DMJ, Wilson JR, et al. Economic evaluation of decompressive craniectomy versus barbiturate coma for refractory intracranial hypertension following traumatic brain injury. *Crit Care Med* 2014; 42: 2235–2243.

32. Behranwala R, Aojula N, Hagana A, et al. An economic evaluation for the use of decompressive craniectomy in the treatment of refractory traumatic intracranial hypertension. *Brain Inj* 2021; 35: 444–452.

For peer review only

For peer review only - http://bmjopen.bmj.com/site/about/guidelines.xhtml

Table 1. Baseline characteristics of UK patients

Characteristics	Craniotomy (N=126)	DC (N=122)
Age (mean±SD) – yr, n	52.3±16.4, 126	51.7±15.9, 122
Male sex – No. /total n (%)	96/126 (76.2)	101/122 (82.8)
Any antithrombotic medication — No. / n (%) [a]	21/115 (18.3)	22/110 (20.0)
Presence of major extracranial injury requiring admission — No. / n (%)	66/123 (53.7)	57/120 (47.5)
Glasgow Coma Scale (GCS) 3-8*	85/120 (70.8)	72/119 (60.5)
Initial CT brain findings		
Presence of midline shift > 5mm — No. / n (%)	106/124 (85.5)	105/121 (86.8)
Compression / absence of basal cisterns — No. / n (%)	101/124 (81.5)	102/121 (84.3)
Presence of parenchymal contusions <25cc — No. / n (%)	58/125 (46.4)	60/121 (49.6)

DC= decompressive craniectomy; **N**=number allocated to that trial arm; No.=number of associated patients; n=number of patients for whom data were available;*A GCS score of 3–8 is defined as 'severe brain injury'.

For peer review only

For peer review only - http://bmjopen.bmj.com/site/about/guidelines.xhtml

Table 2. Levels of resource use according to intervention arm over 12-month treatment period for all UK patients (based on available data)

Resource use	Craniotomy (N=126)	DC (N=122)	P-value‡
Hospital-recorded, index admission			
Primary intervention received, not as randomised, No.	13 (n = 126)	8 (n = 122)	-
Duration of index surgery (hours), mean ± SD (No. / n)	2.57 ± 0.89 (122/122)	2.50 ± 0.93 (110/110)	0.603
ICU length of stay (index admission) (days), mean ± SD (No. / n)	11.85 ± 8.61 (123/126)	13.52 ± 11.28 (121/122)	0.189
NSU length of stay (index admission) (days), mean ± SD (No. / n)	16.75 ± 24.92 (93/122)	21.30 ± 31.10 (99/120)	0.210
Further DCs (index admission), No.	15 (n=116)	4 (n = 116)	-
Further haematoma evacuations (index admission), No.	9 (n = 116)	2 (n = 116)	-
Further wound revisions (index admission), No.	1 (n = 116)	6 (n = 116)	-
Further other cranial operations (index admission)*, No.	3 (n = 116)	2 (n = 2)	-
Hospital-recorded (cranioplasties and shunts (index admission and post-discharge)			
Primary cranioplasties, No.	21 (n = 124)	62 (n = 121)	-
Cranioplasties requiring synthetic plate, No. (%)	17 (81.0%) (n = 21)	46 (74.2%) (n = 62)	-
Cranioplasty revisions, No.	5 (n = 124)	7 (n = 121)	-
Cranioplasties (primary/revisions) requiring re-admission, No.	17 (n = 124)	58 (n = 121)	-
Primary shunts, No.	5 (n = 126)	4 (n = 118)	-
Shunt revisions, No.	5 (n = 126)	2 (n = 118)	-
Shunts (primary/revisions) requiring re-admission, No.	4 (n = 126)	4 (n = 118)	-
Post-discharge cranioplasty/shunt related procedures (combined), No.	21 (n = 124)	61 (n = 118)	-
Patient-reported, post-discharge			
Overnight stay with associated skull/brain operation, No.	13 (n = 111)	32 (n = 95)	-
Any overnight stay excluding skull/brain operation, No. reporting ≥1 stay	61 (n = 111)	54 (n = 95)	-
Overnight stay on rehabilitation unit,† (days), mean ± SD (No. / n)	32.51 ± 63.09 (45/111)	35.10 ± 68.80 (36/90)	0.782

For peer review only - http://bmjopen.bmj.com/site/about/guidelines.xhtml

Overnight stay on NSU,† (days), mean ± SD (No. / n)	0.49 ± 2.00 (8/111)	1.14 ± 5.57 (5/95)	0.252
Overnight stay on ICU,† (days), mean ± SD (No. / n)	0.13 ± 1.33 (1/111)	0.07 ± 0.72 (1/95)	0.731
Overnight stay on other ward,† (days), mean ± SD (No. / n)	4.94 ± 18.08 (20/109)	3.04 ± 17.00 (10/93)	0.447
Healthcare professional contact, N reporting ≥1 visit	64 (n = 109)	47 (n = 94)	-
Hospital doctor (visits), mean ± SD (No. / n)	0.60 ± 1.33 (28/106)	0.61 ± 1.46 (24/92)	0.980
Nurse (visits), mean ± SD (No. / n)	2.20 ± 16.53 (8/107)	0.76 ± 5.28 (7/92)	0.426
General Practitioner (visits), mean ± SD (No. / n)	1.23 ± 2.44 (36/106)	1.09 ± 1.93 (30/93)	0.656
Physiotherapist (visits), mean ± SD (No. / n)	2.38 ± 7.11 (29/105)	4.03 ± 11.19 (19/91)	0.213
Occupational therapist (visits), mean ± SD (No. / n)	1.56 ± 3.41 (32/105)	2.22 ± 7.22 (19/92)	0.407
Speech therapist (visits), mean ± SD (No. / n)	0.55 ± 2.29 (10/107)	0.31 ± 1.40 (9/90)	0.386
Social worker (visits), mean ± SD (No. / n)	0.16 ± 0.77 (6/107)	0.12 ± 0.44 (7/92)	0.665
Community care assistant (visits), mean ± SD (No. / n)	2.68 ± 21.44 (3/106)	2.84 ± 20.45 (3/92)	0.958
Emergency department (visits), mean ± SD (No. / n)	0.10 ± 0.53 (5/107)	0.18 ± 0.61 (10/93)	0.321
Psychologist/neuropsychologist (visits), mean ± SD (No. / n)	0.27 ± 1.24 (7/107)	0.46 ± 2.72 (7/93)	0.514
Other health care professional (visits), mean ± SD (No. / n)	0.03 ± 0.22 (2/107)	0.04 ± 0.33 (2/93)	0.699
Head/brain scan, No. reporting ≥1 scan	47 (n = 111)	44 (n = 93)	-
MRI scans, mean ± SD (No. / n)	0.31 ± 0.62 (27/111)	0.33 ± 0.56 (28/93)	0.745
CT scans, mean ± SD (No. / n)	0.33 ± 0.67 (27/111)	0.45 ± 0.73 (33/93)	0.228
Other scans, mean ± SD (No. / n)	0.04 ± 0.19 (4/111)	0.02 ± 0.15 (2/93)	0.543
Patient-reported, post-discharge (wider resource use)			
Time in a care home (weeks), mean ± SD (No. / n)	1.79 ± 7.14 (10/109)	3.53 ± 10.40 (12/91)	0.164
Help from carer (hours), mean ± SD (No. / n)	971 ± 2,017 (46/99)	1,000 ± 2,225 (36/86)	0.925

DC= decompressive craniectomy; **N**=number allocated to that trial arm; No.=number of patients in receipt of the resource item in question i.e. excluding zero values; n=number of patients for whom data were available; SD=standard deviation; ‡ for the mean cost difference between groups; *Excluding cranioplasties and shunts; †Excluding those reported (by the patient) to be associated with a skull/brain operation (estimates were instead based on hospital-recorded data, see Table S1).

For peer review only - http://bmjopen.bmj.com/site/about/guidelines.xhtml

For peer review only

Table 3. Estimates of mean cost (UK £ sterling, 2018/19) by treatment group over 12-month treatment period for all patients (based on available data)

Cost component	Craniotomy (N=126)	DC(N=122)	P-value‡
Hospital-recorded, index admission			
Index neurosurgical procedure, mean cost ± SD	3,648 ± 1,264 (n=122)	3,560 ± 1,315 (n = 110)	0.603
Length of stay in NSU (index admission), mean cost ± SD	6,109 ± 9,085 (n = 122)	7,766 ± 11,339 (n = 120)	0.210
Length of stay in ICU (index admission), mean cost ± SD	20,039 ± 14,566 (n = 126)	22,873 ± 19,077 (n = 122)	0.189
Further DCs (index admission), mean cost ± SD*	307 ± 859 (n = 116)	82 ± 536 (n = 116)	0.017
Further haematoma evacuations (index admission), mean cost ± SD	165 ± 638 (n = 116)	37 ± 279 (n = 116)	0.048
Further wound revision (index admission), mean cost ± SD	18 ± 198 (n = 116)	110 ± 551 (n = 116)	0.092
Further other cranial operations (index admission),† mean cost ± SD	55 ± 340 (n = 116)	37 ± 279 (n = 116)	0.653
**Total cost per patient (index admission), mean cost ± SD**	**30,790 ± 19,710 (n = 109)**	**34,759 ± 24,481 (n = 102)**	**0.195**
Hospital-recorded cranioplasties and shunts (index admission and post-discharge)			
Cranioplasty procedures, mean cost ± SD	1,059 ± 2,485 (n = 124)	3,055 ± 3,352 (n = 122)	<0.0001
Shunt procedures, mean cost ± SD	212 ± 1,121 (n = 126)	150 ± 834 (n = 118)	0.626
Cranioplasty/shunt same day discount, mean cost ± SD §	-17 ± 132 (n = 124)	0 ± 0 (n = 118)	0.167
**Total cost per patient (cranioplasties and shunts), mean cost ± SD**	**1,258 ± 2,983 (n = 124)**	**3,228 ± 3,677 (n = 118)**	**<0.0001**
Patient-reported, post-discharge			
Overnight stays on rehabilitation unit, mean cost ± SD**	16,375 ± 31,784 (n = 111)	17,677 ± 34,660 (n = 90)	0.782
Overnight stays on NSU, mean cost ± SD**	177 ± 729 (n = 111)	415 ± 2,029 (n = 95)	0.252
Overnight stays on ICU/HDU, mean cost ± SD**	213 ± 2,247 (n = 111)	125 ± 1,215 (n = 95)	0.731
Overnight stays on 'other' ward, mean cost ± SD**	1,746 ± 6,396 (n = 109)	1,076 ± 6,015 (n = 93)	0.447
All healthcare professional visits, mean cost ± SD	682 ± 1,108 (n = 103)	782 ± 1,578 (n = 88)	0.612
All head/brain scans, mean cost ± SD	66 ± 105 (n = 111)	93 ± 101 (n = 93)	0.436
**Total cost per patient (post-discharge PSRQ), mean cost ± SD**	**19,699 ± 34,193 (n = 99)**	**17,948 ± 32,183 (n = 81)**	**0.726**

For peer review only - http://bmjopen.bmj.com/site/about/guidelines.xhtml

For peer review only

Time in a care home (wider perspective only), mean cost ± SD	3,321 ± 13,230 (n = 109)	6,550 ± 19,272 (n = 91)	0.164
Carer time (wider perspective only), mean cost ± SD	16,762 ± 34,828 (n = 99)	17,271 ± 38,419 (n = 86)	0.925
**Overall NHS and PSS cost per patient, mean cost ± SD**	**48,509 ± 46,934 (n = 86)**	**53,573 ± 47,092 (n = 67)**	**0.510**

DC= decompressive craniectomy; **N**=number allocated to that trial arm; n=number of patients for whom data were available; SD=standard deviation; PSS=Personal Social Services; ‡ for the mean cost difference between groups; *Based on mean duration of DC (from all index procedures) of 2.50 (n=110) hours for all randomized patients; †Excluding cranioplasties and shunts; §A discount was applied to account for those shunt and cranioplasty procedures that occurred on the same day and were therefore assumed to be associated with a slightly shorter operation duration and NSU stay; **Overnight stays excluding those associated with a skull/brain operation.

For peer review only

Table 4. Estimates of mean outcomes by treatment group over 12-month treatment period for all patients (based on available data)

Item	Craniotomy (N=126)	DC (N=122)	P-value‡
Baseline EQ-5D-5L score, mean ± SD	0.260 ± 0.353 (n = 87)	0.302 ± 0.366 (n = 91)	0.441
6-month EQ5D-5L score, mean ± SD	0.427 ± 0.392 (n = 102)	0.370 ± 0.393) (n = 94)	0.311
6-month change in EQ5D-5L score, mean ± SD	0.184 ± 0.345 (n = 74)	0.073 ± 0.319 (n = 71)	0.046
12-month EQ5D-5L score, mean ± SD	0.471 ± 0.402 (n = 111)	0.336 ± 0.414 (n = 103)	0.016
12-month change in EQ5D-5L score, mean ± SD	0.218 ± 0.367 (n = 79)	0.073 ± 0.361 (n = 78)	0.013
Total QALY score, mean ± SD	0.351 ± 0.335 (n = 68)	0.338 ± 0.366 (n = 64)	0.830
12-month GOSE score, % favourable**	47.9 (n = 121)	37.4 (n = 115)	0.102

N=number allocated to that trial arm; n=number for whom data were available; DC= decompressive craniectomy; SD=standard deviation; QALY=Quality Adjusted Life Years. GOSE= Extended Glasgow Outcome Scale; GCS= Glasgow Coma Score; ‡ for the mean difference between groups; *Favourable for the GCS score was defined as 9–15 points (moderate to minor brain injury) while unfavourable was defined as 3–8 points (severe brain injury); **Favourable for the GOSE score was defined as upper severe disability or better; †If GCS at baseline is between 3 and 8, a favourable outcome will be defined as upper severe disability or better on 12-month GOSE. If GCS at baseline is between 9 and 15, a favourable outcome will be defined as lower moderate disability or better on 12-month GOSE.

For peer review only

Table 5. Estimates of the mean incremental cost, incremental effect (QALY gain or odds ratio), and cost effectiveness of craniotomy compared with DC in the base-case and two sensitivity analyses (based on imputed data)

**Cost utility analysis**	**Incremental cost (95% CI) (N=126)**	**QALY gain (95% CI) (N=122)**	**ICER**	**CEAC***
Base-case: imputed	-£5,520 (-£18,060 to £7,020)	0.093 (0.029 to 0.156)	Dominant	87%
SA wider cost perspective	-£17,793 (-34,658 to -928)	0.094 (0.030 to 0.159)	Dominant	99%
SA lowest EQ-5D-5L baseline score	-£5,445 (-£17,547 to £6,658)	0.089 (0.025 to 0.152)	Dominant	87%
**Cost effectiveness analysis**	**Incremental cost (95% CI)**	**Odds ratio (95% CI)‡**	**ICER**	
Base-case	-£4,536 (-£17,374 to £8,301)	1.682 (0.995 to 2.842)	Dominant	-
SA wider cost perspective	-£16,900 (-£33,807 to £7)	1.693 (0.998 to 2.871)	Dominant	-

DC= decompressive craniectomy; **N**=number allocated to that trial arm and included in the analysis – imputation was undertaken as part of all presented analyses; 95% CI=95% confidence interval; ICER =incremental cost-effectiveness ratio, described in the Methods; Dominant = lower mean costs and higher mean effect; SA:sensitivity analysis, described in the Methods; QALY=Quality Adjusted Life Years; ‡ for a favourable outcome for craniotomy compared with DC, based on the GOSE (Extended Glasgow Outcome Scale), as described in the Methods; *Probability of being cost-effective on the cost-effectiveness acceptability curve (CEAC) at a threshold of £20,000 per QALY.

For peer review only

For peer review only - http://bmjopen.bmj.com/site/about/guidelines.xhtml

SUPPLEMENTAL MATERIAL (APPENDICES)

APPENDIX 1:

AUTHOR AFFILIATIONS: Norwich Medical School, University of East Anglia, Norwich (Sarah C. Pyne, Garry R. Barton, David A Turner), the Division of Neurosurgery, Addenbrooke's Hospital (Angelos G. Kolias, Carole Turner, Hadie Adams, Midhun Mohan, Edoardo Viaroli, Adel E. Helmy, Ivan S. Timofeev, Peter J. Hutchinson), the Department of Clinical Neurosciences (Harry Mee, Angelos G. Kolias, Carole Turner, Hadie Adams, Midhun Mohan, Matthew G. Stovell, Edoardo Viaroli, Adel E. Helmy, Ivan S. Timofeev, Peter J. Hutchinson), and the Division of Anaesthesia (David K. Menon), University of Cambridge, and the Cambridge Clinical Trials Unit, Cambridge University Hospitals NHS Foundation Trust, Cambridge (Carole Turner), the Neurosurgery Department, Royal London Hospital (Christopher Uff, Shumaila Hasan), the Department of Neurosurgery, Imperial Neurotrauma Centre, Imperial College Academic Health Sciences Centre, St. Mary's Hospital (Mark H. Wilson), London, Wessex Neurological Centre, University Hospitals Southampton NHS Foundation Trust, Southampton (Diederik Bulters, Ardalan Zolnourian), the Department of Neurosurgery, Walton Centre NHS Foundation Trust, Liverpool (Catherine J. McMahon, Matthew G. Stovell), the Department of Neurosurgery and the Academic Directorate of Neurosciences, Sheffield Teaching Hospital NHS Foundation Trust, Sheffield (Yahia Z. Al-Tamimi), the Department of Neurosurgery, Leeds General Infirmary, Leeds (Simon Thomson), the Department of Neurosurgery, University Hospitals Birmingham NHS Foundation Trust, Birmingham (Antonio Belli), the Department of Neurosurgery, Manchester Centre for Clinical Neurosciences, Manchester (Andrew T. King), and the Neurosurgical Trials Group, Wolfson Research Centre, Newcastle University, Newcastle upon Tyne (Barbara Gregson) — all in the United Kingdom.

INVESTIGATORS AND TRIAL OVERSIGHT COMMITTEES

RESCUE-ASDH trial collaborators (to be indexed on PubMed):

- Harry Mee, Angelos G. Kolias, Carole Turner, Hadie Adams, Midhun Mohan, Edoardo Viaroli, Adel E. Helmy, Ivan S. Timofeev, David K. Menon, Peter J. Hutchinson – *University of Cambridge, UK*
- Peter J. Kirkpatrick, Hadie Adams, Nicola Owen, Kirsty Grieve, Alexis Joannides, Ellie Edlmann, Karen Caldwell, Silvia Tarantino, Francesca Hill, Liz Corteen, Janet Corn, Matthew G Stovell, Mathew Guilfoyle, Mohammad Naushahi, Richard Mair, Ibrahim Jalloh, Mark Kotter, Kamila Walker, Tamara Tajsic, Sara Venturini, Selma Tülü – *Cambridge University Hospital, Cambridge, UK*

For peer review only

- Christopher Uff, Shumaila Hasan, Chipo Chitsenega, Geetha Boyapati – *Royal London Hospital, London, UK*
- Muhammad Bhatti, Malik Zaben, Belinda Gunning, Natalia Ermalai, Dmitri Shastin, Mutwakil Abdulla, Liudmila, Joseph Merola – *University Hospital of Wales, Cardiff, UK*
- Stuart Smith, Laurence Glanz, Ashwin Kumaria, Lani Patterson – *Queen's Medical Centre, Nottingham, UK*
- Antonio Belli – *1. NIHR Surgical Reconstruction and Microbiology Research Centre, Birmingham, UK. 2. Institute of Inflammation and Ageing, University of Birmingham, Birmingham, UK*
- Colin Bergin, Maximina Ventura, Emma Toman – *NIHR Surgical Reconstruction and Microbiology Research Centre, Birmingham, UK*
- Giles Critchley, Laura Ortiz-Ruiz de Gordoa, Prasanna Epaliyanage, Husam Georges – *Royal Sussex County Hospital, Brighton, UK*
- Peter Whitfield, Sam Jeffrey, Charlotte Eglinton, Natasha Wilmhurst – *Derriford, Hospital, Plymouth, UK*
- Manjunath Prasad, Shahid Khan, Philip Kane, Emanuel Cirstea – *James Cook University Hospital, Middlesbrough, UK*
- Catherine McMahon, Geraint Sunderland, John Kitchen, Rushid Zakaria – *The Walton Centre, Liverpool, UK*
- Simon Thomson, Soumya Mukherjee, Ian Anderson, Linetty Makawa, Mary Kambafwile – *Leeds General Infirmary, Leeds, UK*
- Marios C. Papadopoulos, Mathew Joseph Gallagher, Siobhan Kearney, Sarah Trippier – *St. George's Hospital, London, UK*
- Mark Wilson, Hani Marcus, Rhys Thomas, Sonia Fernandez Lopez, Louise Young, Andrea D'Mello, Jo-Anna Conyngham – *St. Mary's Hospital, London, UK*
- Diederik Bulters, Ardalan Zolnourian, Patrick Holton, Miriam Taylor, Charlaine Reeve – *Southampton University Hospital, Southampton, UK*
- Jonathan Pollock, Ciaran Hill, Elisa Visentin, Laura Parker – *Queen's Hospital, Romford, UK*
- Kismet Hossain-Ibrahim, Paul Johnston – *Ninewells Hospital, Dundee, UK*
- Vasileios Arzoglou, Emma Clarkson, Arif Zafar, Efosa Ukponmwan – *Hull Royal Infirmary, Hull, UK*
- Christos Tolias, Joseph Frantzias, Anastasios Giamouriadis – *King's College London, London, UK*
- Yahia Al-Tamimi, Adam Wahba, Patrick Easton, Rose Clegg, Grace Cole – *Royal Hallamshire Hospital, Sheffield, UK*

For peer review only

- Damian Holliman, Louise Finlay – *Royal Victoria Infirmary, Newcastle, UK*
- Daniel Holsgrove, Alex Leggate, Jane Perez, Louise Harrison – *Salford Royal Hospital, Manchester, UK*
- Gareth Roberts, Terrie-Louise Cromie, Marianne Hare, Sonia Raj – *Royal Preston Hospital, Preston, UK*
- Tim Lawrence, Ruichong Ma, Javier Magan Ventura, Neil Davidson – *John Radcliffe Hospital, Oxford, UK*
- Richard Nelson, Ruth Womer, Beverley Fulkner – *Frenchay Hospital/North Bristol NHS Trust, Bristol, UK*
- Shabin Joshi, Geraldine Ward – *Walsgrave General Hospital, Coventry, UK*
- Ioannis Fouyas, Julie Woodfield, Himanshu Shekhar, Kareen Damley, Emma Fleming – *Western General Hospital, Edinburgh, UK*
- Roddy O'Kane, Michael Canty – *Queen Elizabeth University Hospital, Glasgow, UK*
- Indira Devi Bhagavatula, Dhananjaya Ishwar Bhat, Dhaval Prem Shukla, Kanti Konar, Nagesh Shanhag, Vaishali NI Valluri – *National Institute of Mental Health and Neurosciences (NIMHANS), Bangalore, India*
- Manoj Kumar Tewari, Manjul Tripathi – *Post Graduate Institute of Medical Education and Research (PGIMER), Chandigarh, India*
- Deepak Gupta, Ashish Bindra, Kaveri Sharma – *All India Institute of Medical Sciences (AIIMS), Delhi, India*
- Mathew Joseph – *Christian Medical College, Vellore, India*
- Nicole Keong, Christine Lock, Chen Min Wei, Julian Han, Janell Kwok, Nicolas Kon Kam King – *National Neuroscience Institute, Singapore*
- Martina Stippler, Emmalin Nelton – *Beth Israel Medical Center, USA*
- Louis Anthony Whitworth, Sonja Stutzman, Christopher Madden, Caryn Harper, Tracey Moore, Vin Shen Ban, Alice Salazar – *The University of Texas Southwestern Medical Center, USA*
- Rocco Armonda, Jason J. Chang, George B. Moses, Patricia Tanjucto – *MedStar Washington Hospital Center, Washington, USA*
- Jamie Ullman, Orseola Arapi, Betsy Moclair – *Northwell Shore University Hospital (Northwell Health), New York, USA*
- Nrupen Baxi, Erin Lewis, Melvin Stone – *Jacobi Medical Center and Montefiore Medical Center, New York, USA*
- John Adair Prall, Meghan Baldwin, Jamie Jones – *Littleton Adventist Hospital (Centura Health), Colorado, USA*
- Clare Gallagher, Ish Bains – *Foothills Medical Centre, Calgary, Canada*

For peer review only

- Kesava Reddy, Paula Carroll – *Hamilton Health Sciences, Ontario, Canada*
- Leodante Da Costa, Nadia Scantlebury – *Sunnybrook Hospital, University of Toronto, Toronto, Canada*
- Fahad Alkherayf, Rafael Ochoa Sanchez, Ioana Moldovan – *The Ottawa Hospital, Ottawa, Canada*
- Sean Christie, Lisa Julien – *Queen Elizabet II Health Sciences Centre, Halifax, Canada*
- Kostas Fountas, Thanasis Paschalis – *University Hospital of Larissa, Medical School (University of Thessaly), Larissa, Greece*
- Sandro Krieg, Sebastian Ille – *Technische Universität München, Munich, Germany*
- Maria Luisa Gandia Gomez – *Hospital Universitario La Paz, Madrid, Spain*
- Alfonso Legares, Ana Maria Castaño Leon – *Hospital Universitario 12 de Octubre, Madrid, Spain*
- András Büki, Gábor Lenzsér – *University of Pécs, Pécs, Hungary*
- Tariq Khan, Mukhtar Khan – *Northwest General Hospital and Research Center, Peshawar, Pakistan*
- Franco Servadei, Massimo Tomei – *Humanitas Research Hospital, Milan, Italy*
- Vairavan Narayanan, Ronie Romelean Jayapalan – *University of Malaya Medical Centre, Kuala Lumpur, Malaysia*
- Martin Hunn, Panagiota Gkolia, Emily Galea – *The Alfred Hospital, Melbourne, Australia*
- Sarah C. Pyne, Garry R. Barton, David A Turner – *Health Economics Group, Norwich Medical School, University of East Anglia, UK*
- Barbara Gregson – *Neurosurgical Trials Group, Wolfson Research Centre, Newcastle University, Newcastle upon Tyne, UK*

<u>Trial co-applicants and/or protocol contributors (to be indexed on PubMed):</u>
Peter J. Hutchinson, Garry R. Barton, Clare Gallagher, Paula Kareclas, Angelos G. Kolias, Peter J. Kirkpatrick Sridevi Nagarajan, David Turner, James Piercy, Peter Kirkpatrick, A. David Mendelow, David K. Menon, Andrew King, Antonio Belli, John D. Pickard, Paul Brennan, Christopher Cowie

<u>Cambridge Clinical Trials Unit staff (to be indexed on PubMed):</u>
Carol Davis-Wilkie, Tapiwa Tungamirai, Kerstin Wolf, Kamila Walker, Natalia Igosheva, Alicia Gore, Paula Kareclas, Michele Jillings, Christopher Bushell

<u>Trial Steering Committee members (to be indexed on PubMed):</u>
Anthony Bell (chair), Allison Hirst, Peter McCabe, Simon Shaw <u>Independent Data Monitoring and Ethics Committee members (to be indexed on PubMed):</u>
Martin Smith (chair), Joan Grieve, Jonathan Cook

For peer review only

APPENDIX 2: BASELINE GCS-ADJUSTED GOSE (SLIDING-DICHOTOMY ANALYSIS)

Methods

In addition to the extended Glasgow Outcome Scale (GOSE) fixed dichotomy analysis described in the main paper, a further GOSE sliding dichotomy analysis was undertaken, in which the favourable/unfavourable categorisation was defined as follows: if GCS (Glasgow Coma Scale) at randomisation was between 3 and 8 (patient comatosed) (1), a favourable outcome was defined as upper severe disability or better but if GCS at randomisation was between 9 and 15 (responsive patient), a favourable outcome was defined as lower moderate disability or better. The cost-effectiveness analysis using this sliding dichotomy, replicated that described in the main paper for the fixed dichotomy, with a view to estimating the cost per additional favourable outcome.

Results and conclusions

For the craniotomy group compared with the DC group, the mean difference in cost was -£6,091 (95% CI -£18,857 to £6,675) with an odds ratio of favourable outcome on the GOSE score of 1.741 (95% CI 1.019 to 2.977). Craniotomy therefore dominated DC.

For peer review only

APPENDIX 3: FURTHER SENSITIVITY ANALYSES

Methods

In addition to the two sensitivity analyses described in the main paper, a further four were defined in the Health Economic Analysis Plan (HEAP) and analysed. The consequence of excluding patient self-reported resource use data (more missing data was expected from this source), and using only hospital-recorded costs was assessed as a sensitivity analysis (SA hospital-recorded post-discharge operations only). Another sensitivity analysis (SA patient-reported post-discharge operations only) included only patient-reported post-discharge skull/brain operations (with associated length of stay) instead of hospital-reported post-discharge cranioplasties and shunts. A further sensitivity analysis (SA per protocol) re-analysed the data on a per protocol basis, excluding patients whose primary treatment was not as allocated, e.g. allocated to DC but received craniotomy and vice versa. A complete case analysis based on the base-case was also undertaken (SA complete case analysis), where participants were only included if they have complete hospital records, participant self-report and QALY data, with no imputation undertaken. These sensitivity analyses were undertaken for both the cost-utility analysis and cost-effectiveness analysis.

Results and Conclusions

The results of the four sensitivity analyses described are presented in Table A1. In all sensitivity analyses, craniotomy was found to dominate DC. This is in keeping with the base-case cost-utility and cost-effectiveness analyses and other sensitivity analyses presented in this paper.

For peer review only

Supplemental Table A1 | Estimates of the mean incremental cost, incremental effect (QALY gain or odds ratio), and cost effectiveness of craniotomy compared with DC for additional sensitivity analyses.

Analysis (N craniotomy,N DC)	Incremental cost (95% CI)	QALY gain (95% CI)	ICER	CEAC*
SA hospital-recorded post-discharge operations only: (126,122) MI	-£6,252 (-£12,180 to -£325)	0.092 (0.031 to 0.153)	Dominant	99%
SA patient-reported post-discharge operations only: (126,122) MI	-£6,328 (-19,389 to £6,733)	0.093 (0.032 to 0.154)	Dominant	89%
SA per protocol: (113,114) MI	-£10,711 (-£23,361 to £1,939)	0.121 (0.056 to 0.185)	Dominant	98%
SA complete case analysis: (60,44)	-£1,917 (-£15,564 to £11,729)	0.071 (-0.0106 to 0.153)	Dominant	68%
**Analysis (N craniotomy,N DC)**	**Incremental cost (95% CI)**	**Odds ratio (95% CI) ‡**	**ICER**	
SA hospital-recorded post-discharge operations only: (126,122)	-£5,709 (-£11,783 to £365)	1.704 (1.010 to 2.888)	Dominant	-
SA patient-reported post-discharge operations only: (126,122)	-£5,374 (-£18,782 to £8,033)	1.687 (0.999 to 2.849	Dominant	-
SA per protocol: (113,114)	-£10,567 (-£23,434 to £2,299)	2.189 (1.252 to 3.827)	Dominant	-
SA complete case analysis: (83,67)	-£4,335 (-£18,545 to £9,876)	1.360 (0.698 to 2.649)	Dominant	-

DC= decompressive craniectomy; 95% CI=95% confidence interval; ICER =incremental cost-effectiveness ratio; Dominant = lower mean costs and higher mean effect; N craniotomy (N DC) = number Randomized to craniotomy/decompressive craniectomy who were included in the analysis; SA:sensitivity analysis, described in the Methods; QALY=Quality Adjusted Life Years; ICER =incremental cost-effectiveness ratio, described in the Methods; *Probability of being cost-effective on the CEAC at a threshold of £20,000 per QALY;‡ for a favourable outcome for craniotomy compared with DC, based on the GOSE (Extended Glasgow Outcome Scale), as described in the Methods.

For peer review only

APPENDIX 4: DEVIATION FROM THE HEAP IN "SA WIDER COST PERSPECTIVE"

Within the Health Economic Analysis Plan (HEAP) it was stated that lost productivity costs would be estimated. Below we explain why this was not undertaken.

The following was stated within the 'Costs' section for the HEAP:

"…Participants were asked to report a) whether they were currently working (paid or unpaid), with the following additional questions (if applicable); b) how many hours per week they work (paid or unpaid); c) whether the number of hours was the same as before their brain injury; d) whether they currently work fewer or more hours per week than before your brain injury; e) when they returned to work following the brain injury; f) whether they have taken any days off due to sickness since returning; g) if they have had to leave work / change job since their brain injury and why. In order to estimate lost productivity, in line with the opportunity cost method (2), the mean lost work time over the 12 month follow-up period (regardless of whether a payment was made) will be estimated and valued at the 2019 UK mean hourly gross wage (£17.25) (3)…"

Within the 'Analysis' section for the HEAP we stated that the base-case analysis would be from the cost perspective of the NHS and PSS. However, it was stated that the first sensitivity analysis (SA) ("SA wider cost perspective" in this paper) would take a more societal perspective and include lost productivity costs, as well as care home and carer costs.

We attempted to include lost productivity costs at the analysis stage but found that we did not have information as to the number of hours participants were working before their brain injury, as intended. The main reason for this was that if a participant reported that they were *not* currently working in response to the above question a) they were not asked to complete questions b-f. In hindsight, this was an error in how the questionnaire was formulated, and they should have been asked to complete questions c and d as well. Considering this error in the framing of the questionnaire we chose to deviate from the HEAP and not estimate lost productivity costs. Consequently, as detailed in the paper, in "SA wider cost perspective" only the care home and carer costs were added to the (base-case) NHS and PSS costs.

For peer review only - http://bmjopen.bmj.com/site/about/guidelines.xhtml

References

1. Bullock MR, Chesnut R, Ghajar J, et al.: Surgical management of acute subdural hematomas. *Neurosurgery* 2006; 58:S2-16-S2-24

2. Hoefman RJ, Van Exel J, Brouwer W: How to include informal care in economic evaluations. *Pharmacoeconomics* 2013; 31:1105–1119

3. Office for National Statistics: Annual Survey of Hours and Earnings (ASHE) Table 1 (revised) [Internet]. 2019. Available from: https://www.ons.gov.uk/employmentandlabourmarket/peopleinwork/earningsandworkinghours/datasets/allemployeesashetable1

For peer review only

SUPPLEMENTAL MATERIAL (TABLES)

Supplemental Table S1. Unit costs, for the 2018/19 financial year

Resource use	Unit cost (£)			Assumptions
Neurosurgical costs				
Index craniotomy or DC (hourly rate)	1,422[1]			Hourly rate applied to the duration of the operation, whether craniotomy or DC. Includes the time from entering pre-med until leaving theatre.
DC, not index procedure (hourly rate)	1,422[1]			Hourly rate applied to two-thirds of the mean length recorded for index DC. This accounts for the presence of previous skin incision and bone cuts.
Cranioplasty (operation cost, index or revision)	2,464[1,2]			Based on hourly rate above and 104 min duration, with an additional cost for both any synthetic material (if applicable, see below) and an additional associated NSU length of stay of 4 days if post-discharge (see below rates).
Haematoma evacuation (all types)	2,132[1]			Based on hourly rate above and 90 min duration (expert opinion)
Wound revision	2,132[1]			Based on hourly rate above and 90 min duration (expert opinion)
'Other' neurosurgical intervention	2,132[1]			Based on hourly rate above and 90 min duration (expert opinion)
Shunt placement (index or revision)	2,132[1]			Based on hourly rate above and 90 min duration (expert opinion) with an additional material cost (see below) and an associated NSU length of stay of 2 days if post-discharge
Synthetic material costs (design/parts) for cranioplasty	2,500			Estimated based on expert opinion (only added if the use of synthetic material was indicated on the relevant form). Not applicable for revisions.
Material costs for shunt	500			Estimated based on expert opinion. Not applicable for revisions.
Over-night stay costs				
Cost per bed day in Neuro-rehabilitation unit	504[3]			
Cost per bed day in NSU	365[4,5]			
Cost per bed day in ICU	1,691[6]			Assumes neurosciences adult patient in critical care, 2 or more organs supported (ICU)
Cost per bed day (other ward type)	354[4,5]			Weighted average of elective and non-elective excess bed days
Health professional visit costs	*Community*	*Hospital*	*Home*	*Assumptions*

For peer review only

Hospital doctor	33.00[4]	186.74[6]	59.40[4,7]	Community: as hospital doctors do not work in the community, the unit cost for a community GP visit was applied.<br>Home: as hospital doctors do not usually visit homes, the unit cost for a home GP visit was applied.
Nurse	12.31[4,7]	69.51[6]	19.64[4,7]	Home: costed as for community visit, plus 12 mins travel time
General Practitioner	33.00[4]	186.74[6]	59.40[4,7]	Hospital: as GPs do not work in hospitals, the unit cost for a hospital doctor visit was applied. Home: costed as for community visit, plus 12 mins travel time.
Physiotherapist	62.90[6]	54.96[6]	69.67[4,6,7]	Home: costed as for community visit, plus 12 mins travel time.
Occupational therapist	83.17[6]	65.54[6]	89.94[4,6,7]	Home: costed as for community visit, plus 12 mins travel time.
Speech therapist	106.51[6]	100.06[6]	113.28[4,6,7]	Home: costed as for community visit, plus 12 mins travel time
Social worker	118.81[4,8]	118.81[4,8]	127.72[4,7,8]	Home: costed as for community visit, plus 12 mins travel time.
Community care assistant	19.87[4,9]	19.87[4,9]	24.64[4,7,9]	Home: costed as for community visit, plus 12 mins travel time.
Emergency department	166.05[6]	166.05[6]	166.05[6]	Single rate costed for an emergency visit
Psychologist/neuropsychologist	141.17[4,10]	146.67[4,10]	156.57[4,7,10]	Home: costed as for community visit, plus 12 mins travel time.
Other	33.00[4]	186.74[6]	69.67[4,6,7]	The cost of the most commonly reported visits from each location are assigned. Community: GP, Hospital: hospital doctor, home: physiotherapist
Other costs				*Assumptions*
MRI scan		120.83[6]		
CT scan		77.95[6]		
Unknown scan		77.95[6]		Assumed the cost of a CT scan
Care home (cost per week in residence)		1,854[11]		As no cost for adults with these specific needs has been estimated, we have used a cost for adults with autism and complex needs.
Carer time		17.27[12]		Gross hourly rate. Used to value carer time whether paid or not
Work time		17.27[12]		Gross hourly rate. Used to value lost work time, assigned to estimated time worked since their brain injury

DC, decompressive craniectomy; ICU, intensive care unit; NSU, neurosurgical care unit; MRI, magnetic resonance imaging; CT, computed tomography
Inflated to 2018/19 financial year prices, where necessary, using the NHSCII pay and prices.[4]

For peer review only

Supplemental Table S2. Proportion of Missing values (%) for key variables

Variable	Craniotomy	DC	Total
Baseline variables			
Treatment allocation	0	0	0
Age	0	0	0
Sex	0	0	0
EQ-5D-5L at baseline	39/126 (31.0%)	31/122 (25.4%)	70/248 (28.2%)
GCS score	6/126 (4.8%)	3/122 (2.5%)	9/248 (3.6%)
Cost variables			
Index admission costs (hospital-recorded data)*	17/126 (13.5%)	20/122 (16.4%)	37/248 (14.9%)
Cranioplasty and shunt costs (hospital-recorded data)†	2/126 (1.6%)	4/122 (3.3%)	6/248 (2.4%)
Post-discharge costs (patient self-report data)	27/126 (21.4%)	41/122 (33.6%)	68/248 (27.4%)
Outcome variables for health-related quality of life			
EQ-5D at 6 months	24/126 (19.1%)	28/122 (23.0%)	52/248 (21.0%)
EQ-5D at 12 months	15/126 (11.9%)	19/122 (15.6%)	34/248 (13.7%)
Outcome variables for GOSE			
GOSE at 6 months	13/126 (10.3%)	16/122 (13.1%)	29/248 (11.7%)
GOSE at 12 months	5/126 (4.0%)	7/122 (5.7%)	12/248 (4.8%)
Outcomes for cost-utility and cost-effectiveness analyses			
Total costs	40/126 (31.8%)	55/122 (45.1%)	95/248 (38.3%)
Total QALYS	58/126 (46.0%)	58/122 (47.5%)	116/248 (46.8%)
Binary GOSE at 12 months	5/126 (4.0%)	7/122 (5.7%)	12/248 (4.8%)
Binary GOSE dependent on GCS at 12 months	11/126 (8.7%)	10/122 (8.2%)	21/248 (8.5%)

*Includes index surgery, length of stay, neurosurgical interventions (excluding cranioplasties and shunts) during index admission.
†Includes cranioplasties and shunts (including revisions) during index admission and post-discharge.

For peer review only

References

1. Public Health Scotland. Scottish health service costs. Costs Book 19 (April 2018 to March 2019). See file R142X, available on the archive web-site, https://webarchive.nrscotland.gov.uk/20231203022410/https:/www.isdscotland.org/Health-Topics/Finance/Costs/File-Listings-2019.asp (2019, accessed 24 April 2024).

2. Fountain DM, Henry J, Honeyman S, et al. First Report of a Multicenter Prospective Registry of Cranioplasty in the United Kingdom and Ireland. *Neurosurgery* 2021; 89: 518–526.

3. Turner-Stokes L, Williams H, Bill A, et al. Cost-efficiency of specialist inpatient rehabilitation for working-aged adults with complex neurological disabilities: a multicentre cohort analysis of a national clinical data set. *BMJ Open* 2016; 6: e010238.

4. Curtis L, Burns A. *Unit costs of health and social care 2019*. Personal Social Services Research Unit, University of Kent, Canterbury, 2019. Epub ahead of print 2019. DOI: 10.22024/UniKent/01.02.79286.

5. Health and social Care Information Centre. *NHS Schedule of Reference Costs 2017/18*. London: Department of Health, 2018.

6. Health and Social Care Information Centre. *NHS Schedule of Reference Costs 2018/19*. London: Department of Health, 2019.

7. Curtis L, Burns A. *Unit costs of health and social care 2015*. Personal Social Services Research Unit, University of Kent, Canterbury, 2015.

8. Curtis L. *Unit costs of health and social care 2010*. Personal Social Services Research Unit, University of Kent, Canterbury, 2010.

9. Curtis L, Burns A. *Unit costs of health and social care 2017*. Personal Social Services Research Unit, University of Kent, Canterbury, 2017. Epub ahead of print 2017. DOI: 10.22024/UniKent/01.02/65559.

For peer review only

10. Curtis L, Burns A. *Unit costs of health and social care 2016*. Personal Social Services Research Unit, University of Kent, Canterbury, 2016.

11. Curtis L, Burns A. *Unit costs of health and social care 2018*. Personal Social Services Research Unit, University of Kent, Canterbury, 2018. Epub ahead of print 2018. DOI: 10.22024/UniKent/01.02.70995.

12. Office for National Statistics. *Annual Survey of Hours and Earnings (ASHE) Table 1 (revised)*, https://www.ons.gov.uk/employmentandlabourmarket/peopleinwork/earningsandworkinghours/datasets/allemployeesashetable1 (2019).

For peer review only

CHEERS 2022 Checklist

Topic	No.	Item	Location where item is reported
**Title**			
	1	Identify the study as an economic evaluation and specify the interventions being compared.	Title page: "Cost-effectiveness of craniotomy versus decompressive craniectomy, for patients with traumatic acute subdural hematoma"
**Abstract**			
	2	Provide a structured summary that highlights context, key methods, results, and alternative analyses.	See Abstract
**Introduction**			
**Background and objectives**	3	Give the context for the study, the study question, and its practical relevance for decision making in policy or practice.	See the 'Background' section
**Methods**			
**Health economic analysis plan**	4	Indicate whether a health economic analysis plan was developed and where available.	In the 'sensitivity analyses' section of the Methods we state that there was "…a pre-specified health economic analysis plan (HEAP) (see: https://www.rescueasdh.org/trial-documents)."
**Study population**	5	Describe characteristics of the study population (such as age range, demographics, socioeconomic, or clinical characteristics).	See the 'Participants' section of the Results and Table 1.
**Setting and location**	6	Provide relevant contextual information that may influence findings.	See the 'Participants' section of the Methods
**Comparators**	7	Describe the interventions or strategies being compared and why chosen.	The interventions are described in the 'Treatment and randomisation' section of the Methods. The rationale is covered in the 'Background' section

Topic	No.	Item	Location where item is reported
**Perspective**	8	State the perspective(s) adopted by the study and why chosen.	Costs were estimated from a UK National Health Service (NHS) and Personal and Social Services (PSS) perspective, as stated in the 'Measuring Costs' section of the Methods
**Time horizon**	9	State the time horizon for the study and why appropriate.	12 month follow-up period (which aligns for that for the trial) is stated in both the 'Measuring Costs' and 'Measuring Outcomes' section of the Methods
**Discount rate**	10	Report the discount rate(s) and reason chosen.	Given the 12 month follow-up period, no discounting was undertaken, as stated in the 'Incremental analyses' section of the Methods
**Selection of outcomes**	11	Describe what outcomes were used as the measure(s) of benefit(s) and harm(s).	See the 'Measuring outcomes' section of the Methods
**Measurement of outcomes**	12	Describe how outcomes used to capture benefit(s) and harm(s) were measured.	See the 'Measuring outcomes' section of the Methods
**Valuation of outcomes**	13	Describe the population and methods used to measure and value outcomes.	See the 'Measuring outcomes' section of the Methods
**Measurement and valuation of resources and costs**	14	Describe how costs were valued.	See the 'Measuring costs' section of the Methods and Supplemental Table S1 for unit costs
**Currency, price date, and conversion**	15	Report the dates of the estimated resource quantities and unit costs, plus the currency and year of conversion.	The dates of the estimated resource quantities are reported in the 'Participants' section of the Results section. Other items are reported in the 'Measuring costs' section of the same chapter
**Rationale and description of model**	16	If modelling is used, describe in detail and why used. Report if the model is publicly available and where it can be accessed.	Not applicable, a within trial cost effectiveness analysis was conducted
**Analytics and assumptions**	17	Describe any methods for analysing or statistically transforming data, any extrapolation methods, and approaches for validating any model used.	See the 'Incremental analyses' section of the Methods

Topic	No.	Item	Location where item is reported
**Characterising heterogeneity**	18	Describe any methods used for estimating how the results of the study vary for subgroups.	See the fifth sensitivity analysis (SA per protocol) in 'Appendix 3: further sensitivity analyses' of the Supplemental Material (Appendices)
**Characterising distributional effects**	19	Describe how impacts are distributed across different individuals or adjustments made to reflect priority populations.	Not reported (Not conducted) – the HEAP was developed before this updated CHEERS checklist was available
**Characterising uncertainty**	20	Describe methods to characterise any sources of uncertainty in the analysis.	See the 'Decision uncertainty' and 'Sensitivity analyses' section of the Methods
**Approach to engagement with patients and others affected by the study**	21	Describe any approaches to engage patients or service recipients, the general public, communities, or stakeholders (such as clinicians or payers) in the design of the study.	The patient self-report questionnaire was developed in consultation with non-trial patients. See the 'Measuring Costs' section of the Methods
**Results**			
**Study parameters**	22	Report all analytic inputs (such as values, ranges, references) including uncertainty or distributional assumptions.	Not applicable, a within trial cost effectiveness analysis was conducted
**Summary of main results**	23	Report the mean values for the main categories of costs and outcomes of interest and summarise them in the most appropriate overall measure.	See Tables 2 (Costs) and 3 (Outcomes), these are referred to in the 'Costs' and 'Outcomes' section of the Results.
**Effect of uncertainty**	24	Describe how uncertainty about analytic judgments, inputs, or projections affect findings. Report the effect of choice of discount rate and time horizon, if applicable.	Sensitivity analyses are reported in Table 5 and Supplemental Table A1. Within these Tables the estimated probability values for the cost-effectiveness acceptability curve (CEAC) are also reported for base-case and sensitivity analyses.
**Effect of engagement with patients and others affected by the study**	25	Report on any difference patient/service recipient, general public, community, or stakeholder involvement made to the approach or findings of the study	The patient self-report questionnaire was developed in consultation with non-trial patients. See the 'Measuring Costs' section of the Methods
**Discussion**			
**Study findings, limitations, generalisability, and current knowledge**	26	Report key findings, limitations, ethical or equity considerations not captured, and how these could affect patients, policy, or practice.	See the Discussion

For peer review only - http://bmjopen.bmj.com/site/about/guidelines.xhtml

Topic	No.	Item	Location where item is reported
**Other relevant information**			
**Source of funding**	27	Describe how the study was funded and any role of the funder in the identification, design, conduct, and reporting of the analysis	See the 'Conflicts of Interest and Source of Funding' section
**Conflicts of interest**	28	Report authors conflicts of interest according to journal or International Committee of Medical Journal Editors requirements.	See the 'Conflicts of Interest and Source of Funding' section

From: Husereau D, Drummond M, Augustovski F, et al. Consolidated Health Economic Evaluation Reporting Standards 2022 (CHEERS 2022) Explanation and Elaboration: A Report of the ISPOR CHEERS II Good Practices Task Force. Value Health 2022;25. doi:10.1016/j.jval.2021.10.008

For peer review only

BMJ Open

Cost-effectiveness of craniotomy versus decompressive craniectomy, for UK patients with traumatic acute subdural hematoma

Journal:	*BMJ Open*
Manuscript ID	bmjopen-2024-085084.R2
Article Type:	Original research
Date Submitted by the Author:	06-May-2024

Complete List of Authors:	Pyne, Sarah; University of East Anglia, Norwich Medical School<br>Barton, Garry ; University of East Anglia Faculty of Medicine and Health Sciences, Norwich Medical School<br>Turner, David; University of East Anglia, Norwich Medical School<br>Mee, Harry; University of Cambridge, Department of Clinical Neurosciences<br>Gregson, Barbara; Newcastle University, Neurosurgical Trials Group, Wolfson Research Centre<br>Kolias, Angelos; Cambridge University, Division of Neurosurgery<br>Turner, Carole; University of Cambridge, Department of Clinical Neurosciences; Addenbrooke's Hospital, Division of Neurosurgery<br>Adams, Hadie; University of Cambridge, Department of Clinical Neurosciences; Addenbrooke's Hospital, Division of Neurosurgery<br>Mohan, Midhun; University of Cambridge, Department of Clinical Neurosciences; Addenbrooke's Hospital, Division of Neurosurgery<br>Uff, Christopher; The Royal London Hospital<br>Hasan, Shumaila; The Royal London Hospital<br>Wilson, Mark; St Mary's Hospital, Department of Neurosurgery<br>Bulters, Diederik ; University Hospital Southampton NHS Foundation Trust<br>Zolnourian, Ardalan; University Hospital Southampton NHS Foundation Trust<br>McMahon, Catherine; The Walton Centre NHS Foundation Trust, Department of Neurosurgery<br>Stovell, Matthew G; University of Cambridge, Department of Clinical Neurosciences<br>Al-Tamimi, YAHIA; Sheffield Teaching Hospitals NHS Foundation Trust, Department of Neurosurgery; Sheffield Teaching Hospitals NHS Foundation Trust, Academic Directorate of Neurosciences<br>Thomson, Simon; Leeds General Infirmary, Department of Neurosurgery<br>Viaroli, Edoardo; Addenbrooke's Hospital, Division of Neurosurgery; University of Cambridge, Department of Clinical Neurosciences<br>Belli, Antonio; University Hospitals Birmingham NHS Foundation Trust, Department of Neurosurgery<br>King, Andrew; Salford Royal Hospital Manchester Centre for Clinical Neurosciences, Department of Neurosurgery<br>Helmy, Adel E; Addenbrooke's Hospital, Division of Neurosurgery; University of Cambridge, Department of Clinical Neurosciences<br>Timofeev, Ivan; University of Cambridge, Department of Clinical

	Neurosciences; Addenbrooke's Hospital, Division of Neurosurgery<br>Menon, David; University of Cambridge, Division of Anaesthesia<br>Hutchinson, Peter; University of Cambridge, Department of Clinical Neurosciences; Addenbrooke's Hospital, Division of Neurosurgery<br>RESCUE-ASDH, trial collaborators; University of Cambridge, Department of Clinical Neurosciences
**Primary Subject Heading**:	Health economics
Secondary Subject Heading:	Neurology, Surgery
Keywords:	HEALTH ECONOMICS, Health economics < HEALTH SERVICES ADMINISTRATION & MANAGEMENT, NEUROSURGERY, Randomized Controlled Trial, Neurosurgery < SURGERY, Brain Injuries

I, the Submitting Author has the right to grant and does grant on behalf of all authors of the Work (as defined in the below author licence), an exclusive licence and/or a non-exclusive licence for contributions from authors who are: i) UK Crown employees; ii) where BMJ has agreed a CC-BY licence shall apply, and/or iii) in accordance with the terms applicable for US Federal Government officers or employees acting as part of their official duties; on a worldwide, perpetual, irrevocable, royalty-free basis to BMJ Publishing Group Ltd ("BMJ") its licensees and where the relevant Journal is co-owned by BMJ to the co-owners of the Journal, to publish the Work in this journal and any other BMJ products and to exploit all rights, as set out in our licence.

The Submitting Author accepts and understands that any supply made under these terms is made by BMJ to the Submitting Author unless you are acting as an employee on behalf of your employer or a postgraduate student of an affiliated institution which is paying any applicable article publishing charge ("APC") for Open Access articles. Where the Submitting Author wishes to make the Work available on an Open Access basis (and intends to pay the relevant APC), the terms of reuse of such Open Access shall be governed by a Creative Commons licence – details of these licences and which Creative Commons licence will apply to this Work are set out in our licence referred to above.

Other than as permitted in any relevant BMJ Author's Self Archiving Policies, I confirm this Work has not been accepted for publication elsewhere, is not being considered for publication elsewhere and does not duplicate material already published. I confirm all authors consent to publication of this Work and authorise the granting of this licence.

For peer review only

Cost-effectiveness of craniotomy versus decompressive craniectomy, for UK patients with traumatic acute subdural hematoma

Authors:

Sarah C Pyne, Garry R Barton*, David A Turner, Harry Mee, Barbara Gregson, Angelos G Kolias, Carole Turner, Hadie Adams, Midhun Mohan, Christopher Uff, Shumaila Hasan, Mark H Wilson, Diederik Bulters, Ardalan Zolnourian, Catherine J McMahon, Matthew G Stovell, Yahia Z Al-Tamimi, Simon Thomson, Edoardo Viaroli, Antonio Belli, Andrew T King, Adel E Helmy, Ivan S Timofeev, David K Menon, Peter J Hutchinson, for the RESCUE-ASDH trial collaborators†

*Corresponding author: Professor Garry Barton, Health Economics Group, Norwich Medical School, University of East Anglia, Norwich NR4 7TJ. Email: g.barton@uea.ac.uk

†Author affiliations, along with a complete list of the investigators and members of the trial oversight committees is provided in the Supplemental Material (Appendix 1).

For peer review only

ABSTRACT

Objective: To estimate the cost-effectiveness of craniotomy, compared with decompressive craniectomy (DC) in UK patients undergoing evacuation of acute subdural haematoma (ASDH).

Design: Economic evaluation undertaken using health resource use and outcome data from the 12-month multi-centre, pragmatic, parallel-group, randomised, RESCUE-ASDH trial.

Setting: UK secondary care.

Participants: 248 UK patients undergoing surgery for traumatic ASDH randomised to craniotomy (N=126) or DC (N=122).

Interventions: Surgical evacuation via craniotomy (bone flap replaced) or decompressive craniectomy (bone flap left out with a view to replace later cranioplasty surgery).

Main outcome measures: In the base-case analysis costs were estimated from an NHS and personal social services perspective. Outcomes were assessed via the Quality-Adjusted Life Years (QALY) derived from the EQ-5D-5L questionnaire (cost-utility analysis) and the Extended Glasgow Outcome Scale (GOSE) (cost-effectiveness analysis). Multiple imputation and regression analyses were conducted to estimate the mean incremental cost and effect of craniotomy compared to DC. The most cost-effective option was selected, irrespective of the level of statistical significance as is argued by economists.

Results: In the cost-utility analysis the mean incremental cost of craniotomy compared to DC was estimated to be -£5,520 (95% confidence interval (CI) -£18,060 to £7,020) with a mean QALY gain of 0.093 (95% CI 0.029 to 0.156). In the cost-effectiveness analysis, the mean incremental cost was estimated to be -£4,536 (95% CI -£17,374 to £8,301) with an odds ratio of 1.682 (95% CI 0.995 to 2.842) for a favourable outcome on the GOSE.

Conclusions: In a UK population with traumatic ASDH craniotomy was estimated to be cost-effective compared to decompressive craniectomy: craniotomy was estimated to have a lower mean cost, higher mean QALY gain and higher probability of a more favourable outcome on the GOSE (though not all estimated differences between the two approaches were statistically significant).

For peer review only - http://bmjopen.bmj.com/site/about/guidelines.xhtml

Trial registration and ethics: Ethical approval for the trial was obtained from the North West – Haydock Research Ethics Committee in the United Kingdom on 17th July 2014 (14/NW/1076). The trial was registered prospectively: ISRCTN87370545.

STRENGTHS AND LIMITATIONS OF THIS STUDY

- This study is based on individual patient-level data from a large, pragmatic, multi-centre randomised trial.
- It is both the first randomised trial and the first economic evaluation to compare craniotomy to decompressive craniectomy.
- Multiple imputation was undertaken to account for missing data.
- For ethical reasons, baseline EQ-5D-5L scores were taken at discharge from neurosurgical unit (NSU), rather than at randomisation.
- A number of sensitivity analyses were undertaken to assess the robustness of conclusions to different assumptions in relation to these and other aspects.

BACKGROUND

In the UK an estimated 1.3 million people live with a traumatic brain injury related disability and the annual societal cost has been estimated to be £15 billion (2015 cost levels).[1] Acute subdural hematoma (ASDH) is a common consequence[2] where craniotomy and decompressive craniectomy (DC) are the two mainstay treatments for surgical evacuation of the hematoma.[3] Both involve the removal of a piece of skull (bone-flap) to evacuate the hematoma. With craniotomy the bone-flap is replaced, whereas with DC it is not. DC may help alleviate brain swelling and is undertaken with the view to a further operation being performed to rebuild the skull (cranioplasty). Craniotomy has the advantage that a patient will not need a later operation to rebuild the skull, but it may fail to control brain swelling in some patients. A systematic review found few studies comparing the two procedures, none of which were randomised, with contrasting evidence as to which was superior.[3]

Given this uncertainty as to whether craniotomy or DC is the more effective treatment for patients with ASDH, the choice of treatment is generally left to the discretion of the surgeon.[3] However, guidance/recommendations for the provision of different treatment options are now often based on estimated levels of cost-effectiveness.[4] Moreover, levels of cost-effectiveness may differ between these two surgical procedures as, for example, DC often requires cranial reconstruction by means of cranioplasty, which has additional costs and a

For peer review only - http://bmjopen.bmj.com/site/about/guidelines.xhtml

significant complication profile,[5] but may better alleviate brain swelling, translating into quality-of-life benefits.[5] Thus, here we report an economic evaluation[6] that was conducted alongside the RESCUE-ASDH (Randomized Evaluation of Surgery with Craniectomy for Patients Undergoing Evacuation of Acute Subdural Hematoma) trial,[5] to compare the cost-effectiveness of craniotomy versus DC for UK patients with traumatic ASDH.

METHODS

Participants

The RESCUE-ASDH trial[5] is a multicentre, international, pragmatic, parallel-group, randomised trial that compared craniotomy with DC. Patients were eligible if they were ≥16 years, had an ASDH on CT scan, and the admitting surgeon felt that the hematoma needed evacuating either by craniotomy or DC. The economic evaluation was nested within the RESCUE-ASDH trial and based on UK participants only.

Treatment and randomisation

Enrolled patients had their ASDH evacuated in the operating room under general anaesthesia. The bone-flap was raised, the dura opened, and the hematoma evacuated, after which patients were randomly assigned to receive either craniotomy (bone-flap restored before skin closure) or DC (bone-flap removed prior to skin closure with a view to being restored later). Patients were only randomised if either treatment was feasible, those patients whose brain was too swollen to allow replacement of the bone flap were not randomised. These patients would have the bone flap left out and were not included in the ITT analysis presented within this paper. As a pragmatic study, management of patients pre-, intra-, and post-operatively was undertaken according to each centre's standard of care.

Blocked randomisation (block size 4) with 1:1 ratio was used, with allocation stratified by geographical region, age group, severity of injury and CT findings.[5] Patients randomised to craniotomy could have a DC at a later stage if their condition deteriorated and at the discretion of the treating clinician. It was not possible to blind patients, relatives and treating clinicians but the primary outcome (see below) was adjudicated centrally by blinded investigators.

Measuring costs

Costs were estimated from a UK NHS and Personal Social Services (PSS) perspective.[7] Resource use data was collected via two methods: hospital-recorded data and a patient self-report (12-month follow-up) questionnaire (PSRQ). Both methods of data collection were developed in consultation with hospital staff/patients and focussed on big cost drivers/resources that were expected to differ between arms.[8] All resource use items that

For peer review only - http://bmjopen.bmj.com/site/about/guidelines.xhtml

were costed (see below) were estimated in £ Sterling for the 2018/19 financial year, resource use items undertaken for research purposes were not costed.

The hospital-recorded data included the following resource use items: details of the intervention (craniotomy or DC) including length of operation and graft details; time spent in the intensive care unit (ICU) and neurosurgical unit (NSU) during initial (index) admission; cranioplasties and shunt placements (these could be received as part of the index admission and/or after discharge from the NSU); any further neurosurgical procedures received during index admission.

The 12-month follow-up PSRQ could be completed by a relative/friend/carer if the participant was unable to complete it and referred only to the time since discharge from NSU. Information requested included the following resource use items: overnight stays in a hospital or other healthcare facility (length of stay, ward type, any associated skull/brain operation); healthcare professional visits (professional seen, frequency and most common location); head/brain scans (MRI, CT or 'other'); time in a care home; help received from a family member/friend or carer.

After assigning unit costs to the resource use items (see Supplemental Table S1 for unit costs), the costs associated with both the hospital-recorded data and PSRQ resource use items, excluding wider societal costs (care home and help/carer costs), were summed to estimate the total NHS and PSS cost per participant. For each group the mean total costs were estimated over the 12-month follow-up period, along with the associated p-value for the mean cost difference between groups. An exception to the above was that, to avoid double-counting, patient self-reported post-discharge overnight stays with an associated skull/brain operation would not be costed if the total reported number was less than the total reported number of hospital-recorded post-discharge cranioplasty and shunt procedures (including revisions). In line with previous work,[9] the higher of the two values was considered the most accurate. It should also be noted that patients who were known to have died post-discharge (mortality is collected as part of the primary outcome, see below) were not sent the PSRQ. As such, post-discharge costs for these participants would have been treated as missing and estimated via imputation (see below for details of the imputation methods, where time post-discharge was included in the MI model). In contrast, cost data for participants who died before discharge from their index admission would not have been considered missing as hospital-recorded data would still have been available for such participants (post-discharge costs were set as equal to zero for such participants).

For peer review only - http://bmjopen.bmj.com/site/about/guidelines.xhtml

Measuring outcomes

To estimate health-related quality-of-life, and conduct a cost-utility analysis,[6] in line with UK NICE guidance,[4,7] the five-dimension EuroQoL five-level questionnaire (EQ-5D-5L)[10] was combined with mortality data to estimate quality-adjusted life-year (QALY)[6] scores. Participants completed the EQ-5D-5L at discharge from NSU (assumed baseline score), 6- and 12-months follow-up (if discharged from NSU by these time points). As recommended at the time of analysis,[11] the crosswalk mapping function[12] was used to convert responses into utility scores (range: -0.594 (worse than death) to 1 (full health)). Participants who died were assigned a utility score of 0 on their date of death (death was collected as part of the hospital-recorded data as it was required for the primary outcome, see below). Utility values were used to estimate QALYs over 12 months, based on the total area under the curve method and linear interpolation.[13]

For ease of interpretation, as is convention,[14] the trial primary outcome measure, the (ordinal) extended Glasgow Outcome Scale (GOSE),[15–18] at 12 months, was converted into a binary scale using a fixed dichotomy analysis (i.e. favourable vs. unfavourable)[5] to enable a cost-effectiveness analysis[6] to be undertaken. Favourable outcomes were defined as upper severe disability or better; while unfavourable outcomes included death, vegetative state and lower severe disability. A sliding dichotomy analysis[5] was also undertaken and is described in the Supplemental Material (Appendix 2).

Missing data

Missing data is common in randomised trials and can lead to bias and lack of precision.[19] As recommended, patterns of missing data were examined to explore the mechanism of missingness.[19] Accordingly, multiple imputation (MI) with chained equations (MICE) under MAR (missing at random) was used to impute missing costs and outcome data, by treatment group. The “mi impute chained” command (Stata 17.0 [StataCorp LP, College Station, TX]) was used to create 30 data sets (based on recommendations in relation to the level of missing data[19]) that were then pooled using Rubin’s rules.[20]

Due to the way data was collected/different levels of missing data, missing data for costs was imputed for total index admission costs (hospital-recorded data collection), total cranioplasty and shunt costs (hospital-recorded data collection over 12-month trial period), and total post-discharge costs (patient self-report questionnaire data collection at 12 months). These three costs were then combined to estimate total NHS and PSS costs. For outcomes, missing data was imputed for utility scores (EQ-5D-5L) at baseline, 6 and 12 months, Glasgow Coma Scale (GCS) score[21,22] at baseline and GOSE score at 12 months.

For peer review only - http://bmjopen.bmj.com/site/about/guidelines.xhtml

In addition to these costs and outcomes, the MI model also included age (years), sex and time post-discharge (the number of days from discharge to the 12-month point or death).

Incremental analyses

For both the cost-utility and cost-effectiveness analyses, a 12-month within-trial, intention-to-treat (ITT) approach was adopted. In this base-case analysis, patients were analysed according to the treatment to which they were randomised, regardless of treatment received. No discounting was undertaken.

For the cost-utility analysis, to estimate the mean incremental cost and incremental effect (QALY gain) associated with craniotomy compared with DC, seemingly unrelated regression (SUR) analysis was undertaken.[23] Regressions included those baseline variables expected to be predictive of total costs and outcomes: age (years), sex and baseline utility score. Assuming dominance,[6] where an intervention was both more costly and less effective, did not occur the incremental cost-effectiveness ratio (ICER = mean incremental cost / mean incremental QALY),[7] for craniotomy versus DC, would be estimated.[6] In the UK, NICE refers to a cost-effectiveness threshold of £20,000 to £30,000 per QALY.[7] As such, if craniotomy had an ICER below this level, this would suggest it is cost-effective, compared with DC. It should be noted that economists have argued that decisions about treatment adoption should be made based on mean estimates, irrespective of whether such differences are statistically significant.[24] Therefore, the treatment option which is estimated to be most cost-effective should be provided.[25] This approach is consistent with the objective of maximizing benefits from a given budget.

For the cost-effectiveness analysis, in terms of the incremental effect, the outcome (based on the GOSE) had a binary scale (favourable/unfavourable) and logistic regression[26] was undertaken to estimate the odds ratio (95% CI) of a favourable outcome for craniotomy compared with DC. Separately, the mean incremental cost associated with craniotomy compared with DC was estimated using linear regression. Both regressions included variables age (years) and sex, which were expected to be predictive of total costs and GOSE outcomes. Together, in the absence of dominance, the incremental cost and incremental effect would enable the ICER to be estimated in terms of the cost per percentage increase in the odds of a favourable outcome.

Decision uncertainty

To estimate the level of uncertainty associated with the decision,[19] the probability of craniotomy being cost-effective, compared with DC, at a threshold of £20,000/QALY on the cost-effectiveness acceptability curve (CEAC)[25] was calculated. This was estimated by

For peer review only - http://bmjopen.bmj.com/site/about/guidelines.xhtml

combining the mean coefficients and covariance matrix from the SUR model, as described in Faria et al.[19] The CEAC was only estimated in relation to the cost-utility analysis.

Sensitivity analyses

The above analysis constituted the base-case analysis[6] and was carried out in accordance with a pre-specified health economic analysis plan (HEAP) (see: https://www.rescueasdh.org/trial-documents). To assess the robustness of conclusions, sensitivity analyses (SA) were undertaken.[6] To analyse the data from a wider cost perspective the care home and carer costs (which were excluded from the base-case analysis) were added to the total NHS and PSS costs (SA wider cost perspective). A further sensitivity analysis (for the cost-utility analysis only) tested the use of the EQ-5D-5L score at discharge from NSU as the baseline for QALY calculations. As any benefits could already have been partially/wholly achieved by discharge, QALY scores were re-estimated with the assumption that, given the grave nature of the condition and following expert advice, participants had the lowest possible EQ-5D-5L score at baseline (date of index surgery): -0.594 (SA lowest EQ-5D-5L baseline score). Four further sensitivity analyses (including a per protocol analysis) were conducted and are presented in the Supplemental Material (Appendix 3). “SA wider cost perspective” deviated from the HEAP, for reasons explained in the Supplemental Material (Appendix 4)

Patient and public involvement

The aforementioned patient self-report questionnaire was developed in consultation with non-trial patients.

RESULTS

Participants

Between September 2014 and April 2019 248 UK patients were recruited, 126 in the craniotomy arm and 122 in the DC arm. Compared with the 450 patients recruited to the full (international) trial (the baseline characteristics of which are summarised in Table 1 of Hutchinson et al. [5]), these UK patients are slightly older (3.5 years on average) and more likely to be on antithrombotic medication (Table 1).

Levels of missing data were slightly lower in the craniotomy group compared with the DC group for cost variables and outcome variables (except at baseline) (Supplemental Table S2).

For peer review only - http://bmjopen.bmj.com/site/about/guidelines.xhtml

Costs

Levels of resource use by intervention arm are summarised in Table 2, under three main categories: (i) Hospital-recorded index-admission; (ii) Hospital-recorded cranioplasties and shunts; (iii) Patient-reported (PSRQ) post-discharge.

The hospital-recorded index-admission data shows that, length of stay in ICU and NSU was slightly lower in the craniotomy group compared with the DC group, but not significantly so. Only small numbers of other neurosurgical operations were reported. With regard to cranioplasties and shunts (index admission and post-discharge), as expected, more patients in the DC group had cranioplasties than in the craniotomy group (DC is pre-requisite to a cranioplasty). There were, however, patients who were randomised to craniotomy that went on to have a DC, 21 of which had a cranioplasty in the 12-month follow-up period. Most cranioplasties used a synthetic material. Shunts were uncommon and occurred at a similar frequency between the groups. In terms of the patient-reported (PSRQ) post-discharge resource use, there were no significant differences between the groups for any of the parameters measured.

Mean cost estimates are summarised in Table 3 and divided into the same three main categories. As expected, given the procedure complexity and recovery time, total NHS and PSS costs are high in both groups. High index admission costs particularly accounted for this, largely due to the high cost of ICU stays, along with post-discharge costs, largely due to the high cost of overnight stays on a rehabilitation unit. There were however few significant differences between groups, the only notable one being the cost of cranioplasty procedures which, for aforementioned reasons, was significantly higher in the DC group. As the number of post-discharge hospital-recorded cranioplasty/shunt procedures exceeded patient-reported over-night stays with an associated skull/brain operation (Table 2), the latter has not been costed.

Outcomes

Outcomes are summarised in Table 4. Follow-up mean EQ-5D-5L scores were higher in the craniotomy group compared with the DC group, significantly so at 12 months. Furthermore, the change (increase) in EQ-5D-5L score from baseline was significantly higher at both 6 and 12 months in the craniotomy group compared with the DC group. There was no significant difference between groups for the total QALY score, based on available data.

At 12 months the percentage of favourable GOSE scores was higher, but not significantly, in the craniotomy group compared with the DC group.

For peer review only - http://bmjopen.bmj.com/site/about/guidelines.xhtml

Analyses

Cost-utility analysis

For the base-case (based on ITT/MI), the mean difference in cost for the craniotomy group compared with the DC group was -£5,520 (95% CI -£18,060 to £7,020) with a mean QALY difference of 0.093 (95% CI 0.029 to 0.156) (Table 5). Craniotomy therefore dominated DC; it was estimated to be associated with both lower costs and more benefit.

Cost-effectiveness analysis

For the craniotomy group compared with the DC group, the mean difference in cost was -£4,536 (95% CI -£17,374 to £8,301) with an odds ratio of favourable outcome on the GOSE score of 1.682 (95% CI 0.995 to 2.842) (Table 5). Again, craniotomy therefore dominated DC.

Decision uncertainty

The base-case probability that craniotomy was cost-effective compared with DC, at a threshold of £20,000/QALY, was 87% (Table 5). This indicates a high degree of certainty associated with the cost-utility analysis decision that craniotomy compared with DC is cost-effective at that threshold.

Sensitivity analyses

In the sensitivity analyses, from a wider cost perspective and using the lowest EQ-5D-5L baseline score (for the cost-utility analysis only), craniotomy was again found to dominate DC (see Table 5). Results of further sensitivity analyses, all of which are consistent with the base-case results, are presented in Supplemental Table A1 (Supplemental Material, Appendix 3).

DISCUSSION

Main findings

In this UK population of patients with traumatic ASDH that warrants surgical evacuation, based on the results of the cost-utility and cost-effectiveness analyses, craniotomy dominated DC as it was estimated to have a lower mean cost, a higher mean QALY gain / higher probability of a more favourable outcome on the GOSE. Craniotomy was therefore estimated to be cost-effective, on the basis that the associated level of significance is considered to be irrelevant.[24,25] In the cost-utility analysis (QALY outcome), there was only an estimated 13% probability (at a threshold of £20,000/QALY) of making the wrong decision by choosing craniotomy. The results of the sensitivity analyses are in keeping with this result.

For peer review only - http://bmjopen.bmj.com/site/about/guidelines.xhtml

Within this study it is important to highlight that costs were estimated from the viewpoint of the UK NHS and PSS and that associated resource use and outcome data was based only on participants from UK sites. As, for example, unit costs may differ outside the UK it is important to note that it is unclear whether these results are generalisable to sites outside the UK. Further associated research may therefore be warranted in relation to this and that ≥20% of patients who were randomised to craniotomy went on to have a DC (as an ITT approach was adopted these patients were included in the craniotomy arm in the base-case analysis).

Strengths and limitations

Regarding health-related quality-of-life, QALY scores (EQ-5D-5L recorded at all time points) were available for 53% of participants only and the amount of missing data was greater at discharge than at 6 and 12 months (Supplemental Table S2). Some missing EQ-5D-5L baseline (NSU discharge) data may be due to participants being discharged at short notice or at the weekend when a research nurse was not available. As some patients had not yet been discharged from hospital by 6 months, this may explain the higher rates of missing data at this time point compared with 12 months. Post-discharge costs (based on patient self-report data) were also missing for 27.4% of patients at 12 months (Supplemental Table S2). Such missing data is a limitation, but we did impute missing data and take an ITT approach, which meant that all patients were still included in the analysis.

A further limitation is that, for ethical reasons, baseline EQ-5D-5L scores were taken at discharge from NSU, rather than at randomisation. Therefore, any benefits could be underestimated by assuming this score is the baseline score. To test the potential impact of this, a sensitivity analysis (SA lowest EQ-5D-5L baseline score) assumed the baseline EQ-5D-5L score to be that of worst possible health state (-0.594). The results differ little from the base-case (Table 5) with craniotomy still dominating DC. It should also be noted that, in the cost-effectiveness analysis, as the cost and outcome regressions are performed separately any correlation between the cost and outcome variables would not be accounted for. A final limitation is that the 12 month follow-up period may not be sufficient to capture all expected cranioplasties. For example, of those randomised to DC (122), only 62 had received a cranioplasty within the 12 month follow-up period. As such, further cranioplasties (aside from those who were randomised to but did not receive DC (8/122) and those who had died (31/122)) could take place beyond the 12 month period. Though this is a limitation, the inclusion of such costs would only be expected to increase the long term incremental cost of DC and therefore not change the conclusion that craniotomy dominated DC.

For peer review only - http://bmjopen.bmj.com/site/about/guidelines.xhtml

Comparisons with other studies

We are not aware of any previous economic evaluations that have specifically compared craniotomy with DC for patients with ASDH. Previous economic evaluations of DC have been undertaken,[27–31] but these have had different comparators, and used a variety of different populations/methods (most developed a decision analytical model to estimate costs and benefits,[29–31] and the two papers[27,28] that used actual patient data were not based on randomised data and were of a smaller sample size than used here, with different cost perspectives and timeframes. Thus, it is difficult to make direct comparisons to our study, and the use of different methods may explain why there were differences in the results as to whether DC was estimated to be cost-effective or not.[27–31]

Implications

In a UK population of patients with traumatic ASDH, craniotomy was estimated to have a lower mean cost, a higher mean QALY gain and a higher probability of a more favourable outcome on the GOSE, dominating DC. Based on the QALY, there was a high probability that craniotomy, compared with DC, was cost-effective (at a threshold of £20,000/QALY). When sensitivity analyses were conducted, the main conclusion (that craniotomy was therefore estimated to be cost-effective) remained unchanged. Consequently, the health economic analysis supports the recommendation, based on the primary outcome,[5] that a craniotomy should be undertaken, rather than a DC, if it is operatively feasible to replace the bone-flap.

For peer review only

For peer review only - http://bmjopen.bmj.com/site/about/guidelines.xhtml

Contributors:

GRB, DAT, HM, BG, AGK, CT, HA, MM, CJM, AB, ATK, DKM and PJH contributed to the conception/design of the work. SCP, GRB, DAT, HM, BG, AGK, CT, HA, MM, CU, SH, MW, DB, AZ, CJM, MGS, YZA, ST, EV, AEH, IST, DKM, PJH contributed to the acquisition of data. SCP, GRB and DT conducted the analysis. All authors contributed to the interpretation of data/drafting of the paper (led by SCP and GRB) and approved the final manuscript.

Competing interests: No support from any organisation other than the National Institute for Health and Care Research was received for the submitted work. Barbara Gregson has received consulting fees from Cambridge University Hospitals NHS Foundation Trust. Angelos Kolias is supported by a Senior Lectureship at the School of Clinical Medicine, University of Cambridge, the Wellcome Trust, and the Royal College of Surgeons of England. Mark Wilson has received support for attending meetings and/or travel for presentations with the Wilderness Medical Society and Royal College of Surgeons of Edinburgh, is a member of the Trauma Clinical Reference group for the NHS, meetings secretary for the Society of British Neurosurgeons and a non-salaried medical director of GoodSAM. Peter Hutchinson is supported by a Research professorship and Senior Investigator award from the NIHR, the NIHR Cambridge Biomedical Research Centre, and the Royal College of Surgeons of England.

Funding statement: This project was supported by the Health Technology Assessment (HTA) Programme (project number 12/35/57) and will be published in full in the HTA journal at https://fundingawards.nihr.ac.uk/award/12/35/57; The RESCUE-ASDH trial is an "embedded study" linked with the CENTER-TBI project (https://www.center-tbi.eu/) of the European Brain Injury Consortium. CENTER-TBI was a large-scale collaborative project, supported by the FP7 Program of the European Union (grant number 602150); RESCUE-ASDH ISRCTN Registry number, ISRCTN87370545.

Study protocol is available at https://fundingawards.nihr.ac.uk/award/12/35/57

The views expressed are those of the authors and not necessarily those of the NHS, the National Institute for Health and Care Research (NIHR), or the Department of Health and Social Care.

We thank the patients who participated in the RESCUE-ASDH trial, their families, and all the collaborating clinicians and research staff, and we thank the staff of the Cambridge Clinical Trials Unit for their support.

Data availability statement: Reasonable requests to make relevant anonymised participant level data available will be considered by the trial team. The trial protocol[14] and a pre-

For peer review only - http://bmjopen.bmj.com/site/about/guidelines.xhtml

specified health economic analysis plan (HEAP) (see: https://www.rescueasdh.org/trial-documents) are also available.

REFERENCES

1. Parsonage M. *Traumatic brain injury and offending. An economic analysis*. 2016.

2. Wilberger JE, Harris M, Diamond DL. Acute subdural hematoma: morbidity, mortality, and operative timing. *J Neurosurg* 1991; 74: 212–8.

3. Phan K, Moore JM, Griessenauer C, et al. Craniotomy versus decompressive craniectomy for acute subdural hematoma: systematic review and meta-analysis. *World Neurosurg* 2017; 101: 677-685.e2.

4. NICE. *NICE health technology evaluations: the manual (PMG 36)*. 2022.

5. Hutchinson PJ, Adams H, Mohan M, et al. Decompressive Craniectomy versus Craniotomy for Acute Subdural Hematoma. *NEJM* 2023; 388: 2219–2229.

6. Drummond M, Sculpher M, Claxton K, et al. *Methods for the economic evaluation of health care programmes*. 4th ed. Oxford, UK: Oxford University Press, 2015.

7. NICE. *Guide to the methods of technology appraisal 2013*, https://www.nice.org.uk/process/pmg9 (2013, accessed 24 April 2024).

8. Ramsey SD, Willke RJ, Glick H, et al. Cost-effectiveness analysis alongside clinical trials II - An ISPOR good research practices task force report. *Value in Health* 2015; 18: 161–172.

9. Byford S, Leese M, Knapp M, et al. Comparison of alternative methods of collection of service use data for the economic evaluation health care interventions. *Health Econ* 2007; 16: 531–536.

10. Herdman M, Gudex C, Lloyd A, et al. Development and preliminary testing of the new five-level version of EQ-5D (EQ-5D-5L). *Qual Life Res* 2011; 20: 1727–36.

11. NICE. *Position statement on use of the EQ-5D-5L value set for England (updated October 2019)*. National Institute for Health and Care Excellence, 2019.

12. Van Hout B, Janssen MF, Feng YS, et al. Interim scoring for the EQ-5D-5L: Mapping the EQ-5D-5L to EQ-5D-3L value sets. *Value in Health* 2012; 15: 708–715.

For peer review only - http://bmjopen.bmj.com/site/about/guidelines.xhtml

13. Manca A, Hawkins N, Sculpher MJ. Estimating mean QALYs in trial-based cost-effectiveness analysis: The importance of controlling for baseline utility. *Health Econ* 2005; 14: 487–496.

14. Hutchinson P. *Randomised Evaluation of Surgery with Craniectomy for patients Undergoing Evacuation of Acute Subdural Haematoma (RESCUE-ASDH) trial protocol version: 3.0*, https://www.fundingawards.nihr.ac.uk/award/12/35/57 (2018, accessed 12 April 2024).

15. Jennett B, Bond M. Assessment of outcome after severe brain damage. *The Lancet* 1975; 1: 480–484.

16. Teasdale GM, Pettigrew LEL, Wilson JTL, et al. Analyzing outcome of treatment of severe head injury: a review and update on advancing the use of the Glasgow Outcome Scale. *J Neurotrauma* 1998; 15: 587–597.

17. Wilson JTL, Slieker FJA, Legrand V, et al. Observer variation in the assessment of outcome in traumatic brain injury: experience from a multicenter, international randomized clinical trial. *Neurosurgery* 2007; 61: 123–128.

18. Wilson JTL, Pettigrew LEL, Teasdale GM. Structured interviews for the Glasgow Outcome Scale and the extended Glasgow Outcome Scale: guidelines for their use. *J Neurotrauma* 1998; 15: 573–580.

19. Faria R, Gomes M, Epstein D, et al. A Guide to Handling Missing Data in Cost-Effectiveness Analysis Conducted Within Randomised Controlled Trials. *Pharmacoeconomics* 2014; 32: 1157–1170.

20. Little R, Rubin D. *Statistical analysis with missing data*. 2nd ed. Hoboken, New Jersey: Wiley, 2002.

21. Teasdale G, Jennett B. Assessment of coma and impaired consciousness. A practical scale. *Lancet* 1974; 2: 81–4.

22. Teasdale G, Murray G, Parker L, et al. Adding up the Glasgow Coma Score. *Acta Neurochir Suppl (Wien)* 1979; 28: 13–6.

23. Willan AR, Briggs AH, Hoch JS. Regression methods for covariate adjustment and subgroup analysis for non-censored cost-effectiveness data. *Health Econ* 2004; 13: 461–475.

24. Claxton K. The irrelevance of inference: A decision-making approach to the stochastic evaluation of health care technologies. *J Health Econ* 1999; 18: 341–364.

For peer review only - http://bmjopen.bmj.com/site/about/guidelines.xhtml

25. Fenwick E, O'Brien BJ, Briggs AH. Cost-effectiveness acceptability curves-facts, fallacies and frequently asked questions. *Health Econ* 2004; 13: 405–415.

26. Hosmer Jr DW, Lemeshow S, Sturdivant RX. *Applied logistic regression*. John Wiley & Sons, 2013.

27. Malmivaara K, Kivisaari R, Hernesniemi J, et al. Cost-effectiveness of decompressive craniectomy in traumatic brain injuries. *Eur J Neurol* 2011; 18: 656–662.

28. Ho KM, Honeybul S, Lind CRP, et al. Cost-effectiveness of decompressive craniectomy as a lifesaving rescue procedure for patients with severe traumatic brain injury. *J Trauma* 2011; 71: 1637–1644.

29. Whitmore RG, Thawani JP, Grady MS, et al. Is aggressive treatment of traumatic brain injury cost-effective? *J Neurosurg* 2012; 116: 1106–1113.

30. Alali AS, Naimark DMJ, Wilson JR, et al. Economic evaluation of decompressive craniectomy versus barbiturate coma for refractory intracranial hypertension following traumatic brain injury. *Crit Care Med* 2014; 42: 2235–2243.

31. Behranwala R, Aojula N, Hagana A, et al. An economic evaluation for the use of decompressive craniectomy in the treatment of refractory traumatic intracranial hypertension. *Brain Inj* 2021; 35: 444–452.

For peer review only

For peer review only - http://bmjopen.bmj.com/site/about/guidelines.xhtml

Table 1. Baseline characteristics of UK patients

Characteristics	Craniotomy (N=126)	DC (N=122)
Age (mean±SD) – yr, n	52.3±16.4, 126	51.7±15.9, 122
Male sex – No. /total n (%)	96/126 (76.2)	101/122 (82.8)
Any antithrombotic medication — No. / n (%) [a]	21/115 (18.3)	22/110 (20.0)
Presence of major extracranial injury requiring admission — No. / n (%)	66/123 (53.7)	57/120 (47.5)
Glasgow Coma Scale (GCS) 3-8*	85/120 (70.8)	72/119 (60.5)
Initial CT brain findings		
Presence of midline shift > 5mm — No. / n (%)	106/124 (85.5)	105/121 (86.8)
Compression / absence of basal cisterns — No. / n (%)	101/124 (81.5)	102/121 (84.3)
Presence of parenchymal contusions <25cc — No. / n (%)	58/125 (46.4)	60/121 (49.6)

DC= decompressive craniectomy; **N**=number allocated to that trial arm; No.=number of associated patients; n=number of patients for whom data were available;*A GCS score of 3–8 is defined as 'severe brain injury'.

For peer review only

Table 2. Levels of resource use according to intervention arm over 12-month treatment period for all UK patients (based on available data)

**Resource use**	**Craniotomy (N=126)**	**DC (N=122)**	**P-value‡**
Hospital-recorded, index admission			
Primary intervention received, not as randomised, No.	13 (n = 126)	8 (n = 122)	-
Duration of index surgery (hours), mean ± SD (No. / n)	2.57 ± 0.89 (122/122)	2.50 ± 0.93 (110/110)	0.603
ICU length of stay (index admission) (days), mean ± SD (No. / n)	11.85 ± 8.61 (123/126)	13.52 ± 11.28 (121/122)	0.189
NSU length of stay (index admission) (days), mean ± SD (No. / n)	16.75 ± 24.92 (93/122)	21.30 ± 31.10 (99/120)	0.210
Further DCs (index admission), No.	15 (n=116)	4 (n = 116)	-
Further haematoma evacuations (index admission), No.	9 (n = 116)	2 (n = 116)	-
Further wound revisions (index admission), No.	1 (n = 116)	6 (n = 116)	-
Further other cranial operations (index admission)*, No.	3 (n = 116)	2 (n = 2)	-
Hospital-recorded (cranioplasties and shunts (index admission and post-discharge)			
Primary cranioplasties, No.	21 (n = 124)	62 (n = 121)	-
Cranioplasties requiring synthetic plate, No. (%)	17 (81.0%) (n = 21)	46 (74.2%) (n = 62)	-
Cranioplasty revisions, No.	5 (n = 124)	7 (n = 121)	-
Cranioplasties (primary/revisions) requiring re-admission, No.	17 (n = 124)	58 (n = 121)	-
Primary shunts, No.	5 (n = 126)	4 (n = 118)	-
Shunt revisions, No.	5 (n = 126)	2 (n = 118)	-
Shunts (primary/revisions) requiring re-admission, No.	4 (n = 126)	4 (n = 118)	-
Post-discharge cranioplasty/shunt related procedures (combined), No.	21 (n = 124)	61 (n = 118)	-
Patient-reported, post-discharge			
Overnight stay with associated skull/brain operation, No.	13 (n = 111)	32 (n = 95)	-
Any overnight stay excluding skull/brain operation, No. reporting ≥1 stay	61 (n = 111)	54 (n = 95)	-
Overnight stay on rehabilitation unit,† (days), mean ± SD (No. / n)	32.51 ± 63.09 (45/111)	35.10 ± 68.80 (36/90)	0.782

For peer review only - http://bmjopen.bmj.com/site/about/guidelines.xhtml

Overnight stay on NSU,† (days), mean ± SD (No. / n)	0.49 ± 2.00 (8/111)	1.14 ± 5.57 (5/95)	0.252
Overnight stay on ICU,† (days), mean ± SD (No. / n)	0.13 ± 1.33 (1/111)	0.07 ± 0.72 (1/95)	0.731
Overnight stay on other ward,† (days), mean ± SD (No. / n)	4.94 ± 18.08 (20/109)	3.04 ± 17.00 (10/93)	0.447
Healthcare professional contact, N reporting ≥1 visit	64 (n = 109)	47 (n = 94)	-
Hospital doctor (visits), mean ± SD (No. / n)	0.60 ± 1.33 (28/106)	0.61 ± 1.46 (24/92)	0.980
Nurse (visits), mean ± SD (No. / n)	2.20 ± 16.53 (8/107)	0.76 ± 5.28 (7/92)	0.426
General Practitioner (visits), mean ± SD (No. / n)	1.23 ± 2.44 (36/106)	1.09 ± 1.93 (30/93)	0.656
Physiotherapist (visits), mean ± SD (No. / n)	2.38 ± 7.11 (29/105)	4.03 ± 11.19 (19/91)	0.213
Occupational therapist (visits), mean ± SD (No. / n)	1.56 ± 3.41 (32/105)	2.22 ± 7.22 (19/92)	0.407
Speech therapist (visits), mean ± SD (No. / n)	0.55 ± 2.29 (10/107)	0.31 ± 1.40 (9/90)	0.386
Social worker (visits), mean ± SD (No. / n)	0.16 ± 0.77 (6/107)	0.12 ± 0.44 (7/92)	0.665
Community care assistant (visits), mean ± SD (No. / n)	2.68 ± 21.44 (3/106)	2.84 ± 20.45 (3/92)	0.958
Emergency department (visits), mean ± SD (No. / n)	0.10 ± 0.53 (5/107)	0.18 ± 0.61 (10/93)	0.321
Psychologist/neuropsychologist (visits), mean ± SD (No. / n)	0.27 ± 1.24 (7/107)	0.46 ± 2.72 (7/93)	0.514
Other health care professional (visits), mean ± SD (No. / n)	0.03 ± 0.22 (2/107)	0.04 ± 0.33 (2/93)	0.699
Head/brain scan, No. reporting ≥1 scan	47 (n = 111)	44 (n = 93)	-
MRI scans, mean ± SD (No. / n)	0.31 ± 0.62 (27/111)	0.33 ± 0.56 (28/93)	0.745
CT scans, mean ± SD (No. / n)	0.33 ± 0.67 (27/111)	0.45 ± 0.73 (33/93)	0.228
Other scans, mean ± SD (No. / n)	0.04 ± 0.19 (4/111)	0.02 ± 0.15 (2/93)	0.543
Patient-reported, post-discharge (wider resource use)			
Time in a care home (weeks), mean ± SD (No. / n)	1.79 ± 7.14 (10/109)	3.53 ± 10.40 (12/91)	0.164
Help from carer (hours), mean ± SD (No. / n)	971 ± 2,017 (46/99)	1,000 ± 2,225 (36/86)	0.925

DC= decompressive craniectomy; **N**=number allocated to that trial arm; No.=number of patients in receipt of the resource item in question i.e. excluding zero values; n=number of patients for whom data were available; SD=standard deviation; ‡ for the mean cost difference between groups; *Excluding cranioplasties and shunts; †Excluding those reported (by the patient) to be associated with a skull/brain operation (estimates were instead based on hospital-recorded data, see Table S1).

For peer review only - http://bmjopen.bmj.com/site/about/guidelines.xhtml

Table 3. Estimates of mean cost (UK £ sterling, 2018/19) by treatment group over 12-month treatment period for all patients (based on available data)

Cost component	Craniotomy (N=126)	DC(N=122)	P-value‡
Hospital-recorded, index admission			
Index neurosurgical procedure, mean cost ± SD	3,648 ± 1,264 (n=122)	3,560 ± 1,315 (n = 110)	0.603
Length of stay in NSU (index admission), mean cost ± SD	6,109 ± 9,085 (n = 122)	7,766 ± 11,339 (n = 120)	0.210
Length of stay in ICU (index admission), mean cost ± SD	20,039 ± 14,566 (n = 126)	22,873 ± 19,077 (n = 122)	0.189
Further DCs (index admission), mean cost ± SD*	307 ± 859 (n = 116)	82 ± 536 (n = 116)	0.017
Further haematoma evacuations (index admission), mean cost ± SD	165 ± 638 (n = 116)	37 ± 279 (n = 116)	0.048
Further wound revision (index admission), mean cost ± SD	18 ± 198 (n = 116)	110 ± 551 (n = 116)	0.092
Further other cranial operations (index admission),† mean cost ± SD	55 ± 340 (n = 116)	37 ± 279 (n = 116)	0.653
**Total cost per patient (index admission), mean cost ± SD**	**30,790 ± 19,710 (n = 109)**	**34,759 ± 24,481 (n = 102)**	**0.195**
Hospital-recorded cranioplasties and shunts (index admission and post-discharge)			
Cranioplasty procedures, mean cost ± SD	1,059 ± 2,485 (n = 124)	3,055 ± 3,352 (n = 122)	<0.0001
Shunt procedures, mean cost ± SD	212 ± 1,121 (n = 126)	150 ± 834 (n = 118)	0.626
Cranioplasty/shunt same day discount, mean cost ± SD §	-17 ± 132 (n = 124)	0 ± 0 (n = 118)	0.167
**Total cost per patient (cranioplasties and shunts), mean cost ± SD**	**1,258 ± 2,983 (n = 124)**	**3,228 ± 3,677 (n = 118)**	**<0.0001**
Patient-reported, post-discharge			
Overnight stays on rehabilitation unit, mean cost ± SD**	16,375 ± 31,784 (n = 111)	17,677 ± 34,660 (n = 90)	0.782
Overnight stays on NSU, mean cost ± SD**	177 ± 729 (n = 111)	415 ± 2,029 (n = 95)	0.252
Overnight stays on ICU/HDU, mean cost ± SD**	213 ± 2,247 (n = 111)	125 ± 1,215 (n = 95)	0.731
Overnight stays on 'other' ward, mean cost ± SD**	1,746 ± 6,396 (n = 109)	1,076 ± 6,015 (n = 93)	0.447
All healthcare professional visits, mean cost ± SD	682 ± 1,108 (n = 103)	782 ± 1,578 (n = 88)	0.612
All head/brain scans, mean cost ± SD	66 ± 105 (n = 111)	93 ± 101 (n = 93)	0.436
**Total cost per patient (post-discharge PSRQ), mean cost ± SD**	**19,699 ± 34,193 (n = 99)**	**17,948 ± 32,183 (n = 81)**	**0.726**

For peer review only - http://bmjopen.bmj.com/site/about/guidelines.xhtml

Time in a care home (wider perspective only), mean cost ± SD	3,321 ± 13,230 (n = 109)	6,550 ± 19,272 (n = 91)	0.164
Carer time (wider perspective only), mean cost ± SD	16,762 ± 34,828 (n = 99)	17,271 ± 38,419 (n = 86)	0.925
**Overall NHS and PSS cost per patient, mean cost ± SD**	**48,509 ± 46,934 (n = 86)**	**53,573 ± 47,092 (n = 67)**	**0.510**

DC= decompressive craniectomy; **N**=number allocated to that trial arm; n=number of patients for whom data were available; SD=standard deviation; PSS=Personal Social Services; ‡ for the mean cost difference between groups; *Based on mean duration of DC (from all index procedures) of 2.50 (n=110) hours for all randomized patients; †Excluding cranioplasties and shunts; §A discount was applied to account for those shunt and cranioplasty procedures that occurred on the same day and were therefore assumed to be associated with a slightly shorter operation duration and NSU stay; **Overnight stays excluding those associated with a skull/brain operation.

For peer review only

Table 4. Estimates of mean outcomes by treatment group over 12-month treatment period for all patients (based on available data)

Item	Craniotomy (N=126)	DC (N=122)	P-value‡
Baseline EQ-5D-5L score, mean ± SD	0.260 ± 0.353 (n = 87)	0.302 ± 0.366 (n = 91)	0.441
6-month EQ5D-5L score, mean ± SD	0.427 ± 0.392 (n = 102)	0.370 ± 0.393 (n = 94)	0.311
6-month change in EQ5D-5L score, mean ± SD	0.184 ± 0.345 (n = 74)	0.073 ± 0.319 (n = 71)	0.046
12-month EQ5D-5L score, mean ± SD	0.471 ± 0.402 (n = 111)	0.336 ± 0.414 (n = 103)	0.016
12-month change in EQ5D-5L score, mean ± SD	0.218 ± 0.367 (n = 79)	0.073 ± 0.361 (n = 78)	0.013
Total QALY score, mean ± SD	0.351 ± 0.335 (n = 68)	0.338 ± 0.366 (n = 64)	0.830
12-month GOSE score, % favourable**	47.9 (n = 121)	37.4 (n = 115)	0.102

N=number allocated to that trial arm; n=number for whom data were available; DC= decompressive craniectomy; SD=standard deviation; QALY=Quality Adjusted Life Years. GOSE= Extended Glasgow Outcome Scale; GCS= Glasgow Coma Score; ‡ for the mean difference between groups; *Favourable for the GCS score was defined as 9–15 points (moderate to minor brain injury) while unfavourable was defined as 3–8 points (severe brain injury); **Favourable for the GOSE score was defined as upper severe disability or better; [†]If GCS at baseline is between 3 and 8, a favourable outcome will be defined as upper severe disability or better on 12-month GOSE. If GCS at baseline is between 9 and 15, a favourable outcome will be defined as lower moderate disability or better on 12-month GOSE.

For peer review only

Table 5. Estimates of the mean incremental cost, incremental effect (QALY gain or odds ratio), and cost effectiveness of craniotomy compared with DC in the base-case and two sensitivity analyses (based on imputed data)

**Cost utility analysis**	**Incremental cost (95% CI) (N=126)**	**QALY gain (95% CI) (N=122)**	**ICER**	**CEAC***
Base-case: imputed	-£5,520 (-£18,060 to £7,020)	0.093 (0.029 to 0.156)	Dominant	87%
SA wider cost perspective	-£17,793 (-34,658 to -928)	0.094 (0.030 to 0.159)	Dominant	99%
SA lowest EQ-5D-5L baseline score	-£5,445 (-£17,547 to £6,658)	0.089 (0.025 to 0.152)	Dominant	87%
**Cost effectiveness analysis**	**Incremental cost (95% CI)**	**Odds ratio (95% CI)‡**	**ICER**	
Base-case	-£4,536 (-£17,374 to £8,301)	1.682 (0.995 to 2.842)	Dominant	-
SA wider cost perspective	-£16,900 (-£33,807 to £7)	1.693 (0.998 to 2.871)	Dominant	-

DC= decompressive craniectomy; **N**=number allocated to that trial arm and included in the analysis – imputation was undertaken as part of all presented analyses; 95% CI=95% confidence interval; ICER =incremental cost-effectiveness ratio, described in the Methods; Dominant = lower mean costs and higher mean effect; SA:sensitivity analysis, described in the Methods; QALY=Quality Adjusted Life Years; ‡ for a favourable outcome for craniotomy compared with DC, based on the GOSE (Extended Glasgow Outcome Scale), as described in the Methods; *Probability of being cost-effective on the cost-effectiveness acceptability curve (CEAC) at a threshold of £20,000 per QALY.

For peer review only - http://bmjopen.bmj.com/site/about/guidelines.xhtml

SUPPLEMENTAL MATERIAL (APPENDICES)

APPENDIX 1:

AUTHOR AFFILIATIONS: Norwich Medical School, University of East Anglia, Norwich (Sarah C. Pyne, Garry R. Barton, David A Turner), the Division of Neurosurgery, Addenbrooke's Hospital (Angelos G. Kolias, Carole Turner, Hadie Adams, Midhun Mohan, Edoardo Viaroli, Adel E. Helmy, Ivan S. Timofeev, Peter J. Hutchinson), the Department of Clinical Neurosciences (Harry Mee, Angelos G. Kolias, Carole Turner, Hadie Adams, Midhun Mohan, Matthew G. Stovell, Edoardo Viaroli, Adel E. Helmy, Ivan S. Timofeev, Peter J. Hutchinson), and the Division of Anaesthesia (David K. Menon), University of Cambridge, and the Cambridge Clinical Trials Unit, Cambridge University Hospitals NHS Foundation Trust, Cambridge (Carole Turner), the Neurosurgery Department, Royal London Hospital (Christopher Uff, Shumaila Hasan), the Department of Neurosurgery, Imperial Neurotrauma Centre, Imperial College Academic Health Sciences Centre, St. Mary's Hospital (Mark H. Wilson), London, Wessex Neurological Centre, University Hospitals Southampton NHS Foundation Trust, Southampton (Diederik Bulters, Ardalan Zolnourian), the Department of Neurosurgery, Walton Centre NHS Foundation Trust, Liverpool (Catherine J. McMahon, Matthew G. Stovell), the Department of Neurosurgery and the Academic Directorate of Neurosciences, Sheffield Teaching Hospital NHS Foundation Trust, Sheffield (Yahia Z. Al-Tamimi), the Department of Neurosurgery, Leeds General Infirmary, Leeds (Simon Thomson), the Department of Neurosurgery, University Hospitals Birmingham NHS Foundation Trust, Birmingham (Antonio Belli), the Department of Neurosurgery, Manchester Centre for Clinical Neurosciences, Manchester (Andrew T. King), and the Neurosurgical Trials Group, Wolfson Research Centre, Newcastle University, Newcastle upon Tyne (Barbara Gregson) — all in the United Kingdom.

INVESTIGATORS AND TRIAL OVERSIGHT COMMITTEES

<u>RESCUE-ASDH trial collaborators (to be indexed on PubMed):</u>

- Harry Mee, Angelos G. Kolias, Carole Turner, Hadie Adams, Midhun Mohan, Edoardo Viaroli, Adel E. Helmy, Ivan S. Timofeev, David K. Menon, Peter J. Hutchinson – *University of Cambridge, UK*
- Peter J. Kirkpatrick, Hadie Adams, Nicola Owen, Kirsty Grieve, Alexis Joannides, Ellie Edlmann, Karen Caldwell, Silvia Tarantino, Francesca Hill, Liz Corteen, Janet Corn, Matthew G Stovell, Mathew Guilfoyle, Mohammad Naushahi, Richard Mair, Ibrahim Jalloh, Mark Kotter, Kamila Walker, Tamara Tajsic, Sara Venturini, Selma Tülü – *Cambridge University Hospital, Cambridge, UK*

For peer review only

- Christopher Uff, Shumaila Hasan, Chipo Chitsenega, Geetha Boyapati – *Royal London Hospital, London, UK*
- Muhammad Bhatti, Malik Zaben, Belinda Gunning, Natalia Ermalai, Dmitri Shastin, Mutwakil Abdulla, Liudmila, Joseph Merola – *University Hospital of Wales, Cardiff, UK*
- Stuart Smith, Laurence Glanz, Ashwin Kumaria, Lani Patterson – *Queen's Medical Centre, Nottingham, UK*
- Antonio Belli – *1. NIHR Surgical Reconstruction and Microbiology Research Centre, Birmingham, UK. 2. Institute of Inflammation and Ageing, University of Birmingham, Birmingham, UK*
- Colin Bergin, Maximina Ventura, Emma Toman – *NIHR Surgical Reconstruction and Microbiology Research Centre, Birmingham, UK*
- Giles Critchley, Laura Ortiz-Ruiz de Gordoa, Prasanna Epaliyanage, Husam Georges – *Royal Sussex County Hospital, Brighton, UK*
- Peter Whitfield, Sam Jeffrey, Charlotte Eglinton, Natasha Wilmhurst – *Derriford, Hospital, Plymouth, UK*
- Manjunath Prasad, Shahid Khan, Philip Kane, Emanuel Cirstea – *James Cook University Hospital, Middlesbrough, UK*
- Catherine McMahon, Geraint Sunderland, John Kitchen, Rushid Zakaria – *The Walton Centre, Liverpool, UK*
- Simon Thomson, Soumya Mukherjee, Ian Anderson, Linetty Makawa, Mary Kambafwile – *Leeds General Infirmary, Leeds, UK*
- Marios C. Papadopoulos, Mathew Joseph Gallagher, Siobhan Kearney, Sarah Trippier – *St. George's Hospital, London, UK*
- Mark Wilson, Hani Marcus, Rhys Thomas, Sonia Fernandez Lopez, Louise Young, Andrea D'Mello, Jo-Anna Conyngham – *St. Mary's Hospital, London, UK*
- Diederik Bulters, Ardalan Zolnourian, Patrick Holton, Miriam Taylor, Charlaine Reeve – *Southampton University Hospital, Southampton, UK*
- Jonathan Pollock, Ciaran Hill, Elisa Visentin, Laura Parker – *Queen's Hospital, Romford, UK*
- Kismet Hossain-Ibrahim, Paul Johnston – *Ninewells Hospital, Dundee, UK*
- Vasileios Arzoglou, Emma Clarkson, Arif Zafar, Efosa Ukponmwan – *Hull Royal Infirmary, Hull, UK*
- Christos Tolias, Joseph Frantzias, Anastasios Giamouriadis – *King's College London, London, UK*
- Yahia Al-Tamimi, Adam Wahba, Patrick Easton, Rose Clegg, Grace Cole – *Royal Hallamshire Hospital, Sheffield, UK*

For peer review only

- Damian Holliman, Louise Finlay – *Royal Victoria Infirmary, Newcastle, UK*
- Daniel Holsgrove, Alex Leggate, Jane Perez, Louise Harrison – *Salford Royal Hospital, Manchester, UK*
- Gareth Roberts, Terrie-Louise Cromie, Marianne Hare, Sonia Raj – *Royal Preston Hospital, Preston, UK*
- Tim Lawrence, Ruichong Ma, Javier Magan Ventura, Neil Davidson – *John Radcliffe Hospital, Oxford, UK*
- Richard Nelson, Ruth Womer, Beverley Fulkner – *Frenchay Hospital/North Bristol NHS Trust, Bristol, UK*
- Shabin Joshi, Geraldine Ward – *Walsgrave General Hospital, Coventry, UK*
- Ioannis Fouyas, Julie Woodfield, Himanshu Shekhar, Kareen Damley, Emma Fleming – *Western General Hospital, Edinburgh, UK*
- Roddy O'Kane, Michael Canty – *Queen Elizabeth University Hospital, Glasgow, UK*
- Indira Devi Bhagavatula, Dhananjaya Ishwar Bhat, Dhaval Prem Shukla, Kanti Konar, Nagesh Shanhag, Vaishali NI Valluri – *National Institute of Mental Health and Neurosciences (NIMHANS), Bangalore, India*
- Manoj Kumar Tewari, Manjul Tripathi – *Post Graduate Institute of Medical Education and Research (PGIMER), Chandigarh, India*
- Deepak Gupta, Ashish Bindra, Kaveri Sharma – *All India Institute of Medical Sciences (AIIMS), Delhi, India*
- Mathew Joseph – *Christian Medical College, Vellore, India*
- Nicole Keong, Christine Lock, Chen Min Wei, Julian Han, Janell Kwok, Nicolas Kon Kam King – *National Neuroscience Institute, Singapore*
- Martina Stippler, Emmalin Nelton – *Beth Israel Medical Center, USA*
- Louis Anthony Whitworth, Sonja Stutzman, Christopher Madden, Caryn Harper, Tracey Moore, Vin Shen Ban, Alice Salazar – *The University of Texas Southwestern Medical Center, USA*
- Rocco Armonda, Jason J. Chang, George B. Moses, Patricia Tanjucto – *MedStar Washington Hospital Center, Washington, USA*
- Jamie Ullman, Orseola Arapi, Betsy Moclair – *Northwell Shore University Hospital (Northwell Health), New York, USA*
- Nrupen Baxi, Erin Lewis, Melvin Stone – *Jacobi Medical Center and Montefiore Medical Center, New York, USA*
- John Adair Prall, Meghan Baldwin, Jamie Jones – *Littleton Adventist Hospital (Centura Health), Colorado, USA*
- Clare Gallagher, Ish Bains – *Foothills Medical Centre, Calgary, Canada*

For peer review only

- Kesava Reddy, Paula Carroll – *Hamilton Health Sciences, Ontario, Canada*
- Leodante Da Costa, Nadia Scantlebury – *Sunnybrook Hospital, University of Toronto, Toronto, Canada*
- Fahad Alkherayf, Rafael Ochoa Sanchez, Ioana Moldovan – *The Ottawa Hospital, Ottawa, Canada*
- Sean Christie, Lisa Julien – *Queen Elizabet II Health Sciences Centre, Halifax, Canada*
- Kostas Fountas, Thanasis Paschalis – *University Hospital of Larissa, Medical School (University of Thessaly), Larissa, Greece*
- Sandro Krieg, Sebastian Ille – *Technische Universität München, Munich, Germany*
- Maria Luisa Gandia Gomez – *Hospital Universitario La Paz, Madrid, Spain*
- Alfonso Legares, Ana Maria Castaño Leon – *Hospital Universitario 12 de Octubre, Madrid, Spain*
- András Büki, Gábor Lenzsér – *University of Pécs, Pécs, Hungary*
- Tariq Khan, Mukhtar Khan – *Northwest General Hospital and Research Center, Peshawar, Pakistan*
- Franco Servadei, Massimo Tomei – *Humanitas Research Hospital, Milan, Italy*
- Vairavan Narayanan, Ronie Romelean Jayapalan – *University of Malaya Medical Centre, Kuala Lumpur, Malaysia*
- Martin Hunn, Panagiota Gkolia, Emily Galea – *The Alfred Hospital, Melbourne, Australia*
- Sarah C. Pyne, Garry R. Barton, David A Turner – *Health Economics Group, Norwich Medical School, University of East Anglia, UK*
- Barbara Gregson – *Neurosurgical Trials Group, Wolfson Research Centre, Newcastle University, Newcastle upon Tyne, UK*

Trial co-applicants and/or protocol contributors (to be indexed on PubMed):

Peter J. Hutchinson, Garry R. Barton, Clare Gallagher, Paula Kareclas, Angelos G. Kolias, Peter J. Kirkpatrick Sridevi Nagarajan, David Turner, James Piercy, Peter Kirkpatrick, A. David Mendelow, David K. Menon, Andrew King, Antonio Belli, John D. Pickard, Paul Brennan, Christopher Cowie

Cambridge Clinical Trials Unit staff (to be indexed on PubMed):

Carol Davis-Wilkie, Tapiwa Tungamirai, Kerstin Wolf, Kamila Walker, Natalia Igosheva, Alicia Gore, Paula Kareclas, Michele Jillings, Christopher Bushell

Trial Steering Committee members (to be indexed on PubMed):

Anthony Bell (chair), Allison Hirst, Peter McCabe, Simon Shaw Independent Data Monitoring and Ethics Committee members (to be indexed on PubMed):

Martin Smith (chair), Joan Grieve, Jonathan Cook

For peer review only

APPENDIX 2: BASELINE GCS-ADJUSTED GOSE (SLIDING-DICHOTOMY ANALYSIS)

Methods

In addition to the extended Glasgow Outcome Scale (GOSE) fixed dichotomy analysis described in the main paper, a further GOSE sliding dichotomy analysis was undertaken, in which the favourable/unfavourable categorisation was defined as follows: if GCS (Glasgow Coma Scale) at randomisation was between 3 and 8 (patient comatosed) (1), a favourable outcome was defined as upper severe disability or better but if GCS at randomisation was between 9 and 15 (responsive patient), a favourable outcome was defined as lower moderate disability or better. The cost-effectiveness analysis using this sliding dichotomy, replicated that described in the main paper for the fixed dichotomy, with a view to estimating the cost per additional favourable outcome.

Results and conclusions

For the craniotomy group compared with the DC group, the mean difference in cost was -£6,091 (95% CI -£18,857 to £6,675) with an odds ratio of favourable outcome on the GOSE score of 1.741 (95% CI 1.019 to 2.977). Craniotomy therefore dominated DC.

For peer review only

APPENDIX 3: FURTHER SENSITIVITY ANALYSES

Methods

In addition to the two sensitivity analyses described in the main paper, a further four were defined in the Health Economic Analysis Plan (HEAP) and analysed. The consequence of excluding patient self-reported resource use data (more missing data was expected from this source), and using only hospital-recorded costs was assessed as a sensitivity analysis (SA hospital-recorded post-discharge operations only). Another sensitivity analysis (SA patient-reported post-discharge operations only) included only patient-reported post-discharge skull/brain operations (with associated length of stay) instead of hospital-reported post-discharge cranioplasties and shunts. A further sensitivity analysis (SA per protocol) re-analysed the data on a per protocol basis, excluding patients whose primary treatment was not as allocated, e.g. allocated to DC but received craniotomy and vice versa. A complete case analysis based on the base-case was also undertaken (SA complete case analysis), where participants were only included if they have complete hospital records, participant self-report and QALY data, with no imputation undertaken. These sensitivity analyses were undertaken for both the cost-utility analysis and cost-effectiveness analysis.

Results and Conclusions

The results of the four sensitivity analyses described are presented in Table A1. In all sensitivity analyses, craniotomy was found to dominate DC. This is in keeping with the base-case cost-utility and cost-effectiveness analyses and other sensitivity analyses presented in this paper.

For peer review only

Supplemental Table A1 | Estimates of the mean incremental cost, incremental effect (QALY gain or odds ratio), and cost effectiveness of craniotomy compared with DC for additional sensitivity analyses.

Analysis (N craniotomy,N DC)	Incremental cost (95% CI)	QALY gain (95% CI)	ICER	CEAC*
SA hospital-recorded post-discharge operations only: (126,122) MI	-£6,252 (-£12,180 to -£325)	0.092 (0.031 to 0.153)	Dominant	99%
SA patient-reported post-discharge operations only: (126,122) MI	-£6,328 (-19,389 to £6,733)	0.093 (0.032 to 0.154)	Dominant	89%
SA per protocol: (113,114) MI	-£10,711 (-£23,361 to £1,939)	0.121 (0.056 to 0.185)	Dominant	98%
SA complete case analysis: (60,44)	-£1,917 (-£15,564 to £11,729)	0.071 (-0.0106 to 0.153)	Dominant	68%
**Analysis (N craniotomy,N DC)**	**Incremental cost (95% CI)**	**Odds ratio (95% CI) ‡**	**ICER**	
SA hospital-recorded post-discharge operations only: (126,122)	-£5,709 (-£11,783 to £365)	1.704 (1.010 to 2.888)	Dominant	-
SA patient-reported post-discharge operations only: (126,122)	-£5,374 (-£18,782 to £8,033)	1.687 (0.999 to 2.849	Dominant	-
SA per protocol: (113,114)	-£10,567 (-£23,434 to £2,299)	2.189 (1.252 to 3.827)	Dominant	-
SA complete case analysis: (83,67)	-£4,335 (-£18,545 to £9,876)	1.360 (0.698 to 2.649)	Dominant	-

DC= decompressive craniectomy; 95% CI=95% confidence interval; ICER =incremental cost-effectiveness ratio; Dominant = lower mean costs and higher mean effect; N craniotomy (N DC) = number Randomized to craniotomy/decompressive craniectomy who were included in the analysis; SA:sensitivity analysis, described in the Methods; QALY=Quality Adjusted Life Years; ICER =incremental cost-effectiveness ratio, described in the Methods; *Probability of being cost-effective on the CEAC at a threshold of £20,000 per QALY;‡ for a favourable outcome for craniotomy compared with DC, based on the GOSE (Extended Glasgow Outcome Scale), as described in the Methods.

For peer review only

APPENDIX 4: DEVIATION FROM THE HEAP IN “SA WIDER COST PERSPECTIVE”

Within the Health Economic Analysis Plan (HEAP) it was stated that lost productivity costs would be estimated. Below we explain why this was not undertaken.

The following was stated within the ‘Costs’ section for the HEAP:

“…Participants were asked to report a) whether they were currently working (paid or unpaid), with the following additional questions (if applicable); b) how many hours per week they work (paid or unpaid); c) whether the number of hours was the same as before their brain injury; d) whether they currently work fewer or more hours per week than before your brain injury; e) when they returned to work following the brain injury; f) whether they have taken any days off due to sickness since returning; g) if they have had to leave work / change job since their brain injury and why. In order to estimate lost productivity, in line with the opportunity cost method (2), the mean lost work time over the 12 month follow-up period (regardless of whether a payment was made) will be estimated and valued at the 2019 UK mean hourly gross wage (£17.25) (3)…“

Within the ‘Analysis’ section for the HEAP we stated that the base-case analysis would be from the cost perspective of the NHS and PSS. However, it was stated that the first sensitivity analysis (SA) (“SA wider cost perspective” in this paper) would take a more societal perspective and include lost productivity costs, as well as care home and carer costs.

We attempted to include lost productivity costs at the analysis stage but found that we did not have information as to the number of hours participants were working before their brain injury, as intended. The main reason for this was that if a participant reported that they were *not* currently working in response to the above question a) they were not asked to complete questions b-f. In hindsight, this was an error in how the questionnaire was formulated, and they should have been asked to complete questions c and d as well. Considering this error in the framing of the questionnaire we chose to deviate from the HEAP and not estimate lost productivity costs. Consequently, as detailed in the paper, in “SA wider cost perspective” only the care home and carer costs were added to the (base-case) NHS and PSS costs.

For peer review only

References

1. Bullock MR, Chesnut R, Ghajar J, et al.: Surgical management of acute subdural hematomas. *Neurosurgery* 2006; 58:S2-16-S2-24

2. Hoefman RJ, Van Exel J, Brouwer W: How to include informal care in economic evaluations. *Pharmacoeconomics* 2013; 31:1105–1119

3. Office for National Statistics: Annual Survey of Hours and Earnings (ASHE) Table 1 (revised) [Internet]. 2019. Available from: https://www.ons.gov.uk/employmentandlabourmarket/peopleinwork/earningsandworkinghours/datasets/allemployeesashetable1

For peer review only

SUPPLEMENTAL MATERIAL (TABLES)

Supplemental Table S1. Unit costs, for the 2018/19 financial year

Resource use	Unit cost (£)	Assumptions
Neurosurgical costs		
Index craniotomy or DC (hourly rate)	1,422[1]	Hourly rate applied to the duration of the operation, whether craniotomy or DC. Includes the time from entering pre-med until leaving theatre.
DC, not index procedure (hourly rate)	1,422[1]	Hourly rate applied to two-thirds of the mean length recorded for index DC. This accounts for the presence of previous skin incision and bone cuts.
Cranioplasty (operation cost, index or revision)	2,464[1,2]	Based on hourly rate above and 104 min duration, with an additional cost for both any synthetic material (if applicable, see below) and an additional associated NSU length of stay of 4 days if post-discharge (see below rates).
Haematoma evacuation (all types)	2,132[1]	Based on hourly rate above and 90 min duration (expert opinion)
Wound revision	2,132[1]	Based on hourly rate above and 90 min duration (expert opinion)
'Other' neurosurgical intervention	2,132[1]	Based on hourly rate above and 90 min duration (expert opinion)
Shunt placement (index or revision)	2,132[1]	Based on hourly rate above and 90 min duration (expert opinion) with an additional material cost (see below) and an associated NSU length of stay of 2 days if post-discharge
Synthetic material costs (design/parts) for cranioplasty	2,500	Estimated based on expert opinion (only added if the use of synthetic material was indicated on the relevant form). Not applicable for revisions.
Material costs for shunt	500	Estimated based on expert opinion. Not applicable for revisions.
Over-night stay costs		
Cost per bed day in Neuro-rehabilitation unit	504[3]	
Cost per bed day in NSU	365[4,5]	
Cost per bed day in ICU	1,691[6]	Assumes neurosciences adult patient in critical care, 2 or more organs supported (ICU)
Cost per bed day (other ward type)	354[4,5]	Weighted average of elective and non-elective excess bed days

Health professional visit costs	*Community*	*Hospital*	*Home*	*Assumptions*

For peer review only

Hospital doctor	33.00[4]	186.74[6]	59.40[4,7]	Community: as hospital doctors do not work in the community, the unit cost for a community GP visit was applied. Home: as hospital doctors do not usually visit homes, the unit cost for a home GP visit was applied.
Nurse	12.31[4,7]	69.51[6]	19.64[4,7]	Home: costed as for community visit, plus 12 mins travel time
General Practitioner	33.00[4]	186.74[6]	59.40[4,7]	Hospital: as GPs do not work in hospitals, the unit cost for a hospital doctor visit was applied. Home: costed as for community visit, plus 12 mins travel time.
Physiotherapist	62.90[6]	54.96[6]	69.67[4,6,7]	Home: costed as for community visit, plus 12 mins travel time.
Occupational therapist	83.17[6]	65.54[6]	89.94[4,6,7]	Home: costed as for community visit, plus 12 mins travel time.
Speech therapist	106.51[6]	100.06[6]	113.28[4,6,7]	Home: costed as for community visit, plus 12 mins travel time
Social worker	118.81[4,8]	118.81[4,8]	127.72[4,7,8]	Home: costed as for community visit, plus 12 mins travel time.
Community care assistant	19.87[4,9]	19.87[4,9]	24.64[4,7,9]	Home: costed as for community visit, plus 12 mins travel time.
Emergency department	166.05[6]	166.05[6]	166.05[6]	Single rate costed for an emergency visit
Psychologist/neuropsychologist	141.17[4,10]	146.67[4,10]	156.57[4,7,10]	Home: costed as for community visit, plus 12 mins travel time.
Other	33.00[4]	186.74[6]	69.67[4,6,7]	The cost of the most commonly reported visits from each location are assigned. Community: GP, Hospital: hospital doctor, home: physiotherapist

Other costs		*Assumptions*
MRI scan	120.83[6]	
CT scan	77.95[6]	
Unknown scan	77.95[6]	Assumed the cost of a CT scan
Care home (cost per week in residence)	1,854[11]	As no cost for adults with these specific needs has been estimated, we have used a cost for adults with autism and complex needs.
Carer time	17.27[12]	Gross hourly rate. Used to value carer time whether paid or not
Work time	17.27[12]	Gross hourly rate. Used to value lost work time, assigned to estimated time worked since their brain injury

DC, decompressive craniectomy; ICU, intensive care unit; NSU, neurosurgical care unit; MRI, magnetic resonance imaging; CT, computed tomography
Inflated to 2018/19 financial year prices, where necessary, using the NHSCII pay and prices.[4]

For peer review only

Supplemental Table S2. Proportion of Missing values (%) for key variables

Variable	Craniotomy	DC	Total
Baseline variables			
Treatment allocation	0	0	0
Age	0	0	0
Sex	0	0	0
EQ-5D-5L at baseline	39/126 (31.0%)	31/122 (25.4%)	70/248 (28.2%)
GCS score	6/126 (4.8%)	3/122 (2.5%)	9/248 (3.6%)
Cost variables			
Index admission costs (hospital-recorded data)*	17/126 (13.5%)	20/122 (16.4%)	37/248 (14.9%)
Cranioplasty and shunt costs (hospital-recorded data)†	2/126 (1.6%)	4/122 (3.3%)	6/248 (2.4%)
Post-discharge costs (patient self-report data)	27/126 (21.4%)	41/122 (33.6%)	68/248 (27.4%)
Outcome variables for health-related quality of life			
EQ-5D at 6 months	24/126 (19.1%)	28/122 (23.0%)	52/248 (21.0%)
EQ-5D at 12 months	15/126 (11.9%)	19/122 (15.6%)	34/248 (13.7%)
Outcome variables for GOSE			
GOSE at 6 months	13/126 (10.3%)	16/122 (13.1%)	29/248 (11.7%)
GOSE at 12 months	5/126 (4.0%)	7/122 (5.7%)	12/248 (4.8%)
Outcomes for cost-utility and cost-effectiveness analyses			
Total costs	40/126 (31.8%)	55/122 (45.1%)	95/248 (38.3%)
Total QALYS	58/126 (46.0%)	58/122 (47.5%)	116/248 (46.8%)
Binary GOSE at 12 months	5/126 (4.0%)	7/122 (5.7%)	12/248 (4.8%)
Binary GOSE dependent on GCS at 12 months	11/126 (8.7%)	10/122 (8.2%)	21/248 (8.5%)

*Includes index surgery, length of stay, neurosurgical interventions (excluding cranioplasties and shunts) during index admission.
†Includes cranioplasties and shunts (including revisions) during index admission and post-discharge.

For peer review only

References

1. Public Health Scotland. Scottish health service costs. Costs Book 19 (April 2018 to March 2019). See file R142X, available on the archive web-site, https://webarchive.nrscotland.gov.uk/20231203022410/https:/www.isdscotland.org/Health-Topics/Finance/Costs/File-Listings-2019.asp (2019, accessed 24 April 2024).

2. Fountain DM, Henry J, Honeyman S, et al. First Report of a Multicenter Prospective Registry of Cranioplasty in the United Kingdom and Ireland. *Neurosurgery* 2021; 89: 518–526.

3. Turner-Stokes L, Williams H, Bill A, et al. Cost-efficiency of specialist inpatient rehabilitation for working-aged adults with complex neurological disabilities: a multicentre cohort analysis of a national clinical data set. *BMJ Open* 2016; 6: e010238.

4. Curtis L, Burns A. *Unit costs of health and social care 2019*. Personal Social Services Research Unit, University of Kent, Canterbury, 2019. Epub ahead of print 2019. DOI: 10.22024/UniKent/01.02.79286.

5. Health and social Care Information Centre. *NHS Schedule of Reference Costs 2017/18*. London: Department of Health, 2018.

6. Health and Social Care Information Centre. *NHS Schedule of Reference Costs 2018/19*. London: Department of Health, 2019.

7. Curtis L, Burns A. *Unit costs of health and social care 2015*. Personal Social Services Research Unit, University of Kent, Canterbury, 2015.

8. Curtis L. *Unit costs of health and social care 2010*. Personal Social Services Research Unit, University of Kent, Canterbury, 2010.

9. Curtis L, Burns A. *Unit costs of health and social care 2017*. Personal Social Services Research Unit, University of Kent, Canterbury, 2017. Epub ahead of print 2017. DOI: 10.22024/UniKent/01.02/65559.

For peer review only

10. Curtis L, Burns A. *Unit costs of health and social care 2016*. Personal Social Services Research Unit, University of Kent, Canterbury, 2016.

11. Curtis L, Burns A. *Unit costs of health and social care 2018*. Personal Social Services Research Unit, University of Kent, Canterbury, 2018. Epub ahead of print 2018. DOI: 10.22024/UniKent/01.02.70995.

12. Office for National Statistics. *Annual Survey of Hours and Earnings (ASHE) Table 1 (revised)*, https://www.ons.gov.uk/employmentandlabourmarket/peopleinwork/earningsandworkinghours/datasets/allemployeesashetable1 (2019).

For peer review only

CHEERS 2022 Checklist

Topic	No.	Item	Location where item is reported
**Title**			
	1	Identify the study as an economic evaluation and specify the interventions being compared.	Title page: "Cost-effectiveness of craniotomy versus decompressive craniectomy, for patients with traumatic acute subdural hematoma"
**Abstract**			
	2	Provide a structured summary that highlights context, key methods, results, and alternative analyses.	See Abstract
**Introduction**			
**Background and objectives**	3	Give the context for the study, the study question, and its practical relevance for decision making in policy or practice.	See the 'Background' section
**Methods**			
**Health economic analysis plan**	4	Indicate whether a health economic analysis plan was developed and where available.	In the 'sensitivity analyses' section of the Methods we state that there was "…a pre-specified health economic analysis plan (HEAP) (see: https://www.rescueasdh.org/trial-documents)."
**Study population**	5	Describe characteristics of the study population (such as age range, demographics, socioeconomic, or clinical characteristics).	See the 'Participants' section of the Results and Table 1.
**Setting and location**	6	Provide relevant contextual information that may influence findings.	See the 'Participants' section of the Methods
**Comparators**	7	Describe the interventions or strategies being compared and why chosen.	The interventions are described in the 'Treatment and randomisation' section of the Methods. The rationale is covered in the 'Background' section

Topic	No.	Item	Location where item is reported
**Perspective**	8	State the perspective(s) adopted by the study and why chosen.	Costs were estimated from a UK National Health Service (NHS) and Personal and Social Services (PSS) perspective, as stated in the 'Measuring Costs' section of the Methods
**Time horizon**	9	State the time horizon for the study and why appropriate.	12 month follow-up period (which aligns for that for the trial) is stated in both the 'Measuring Costs' and 'Measuring Outcomes' section of the Methods
**Discount rate**	10	Report the discount rate(s) and reason chosen.	Given the 12 month follow-up period, no discounting was undertaken, as stated in the 'Incremental analyses' section of the Methods
**Selection of outcomes**	11	Describe what outcomes were used as the measure(s) of benefit(s) and harm(s).	See the 'Measuring outcomes' section of the Methods
**Measurement of outcomes**	12	Describe how outcomes used to capture benefit(s) and harm(s) were measured.	See the 'Measuring outcomes' section of the Methods
**Valuation of outcomes**	13	Describe the population and methods used to measure and value outcomes.	See the 'Measuring outcomes' section of the Methods
**Measurement and valuation of resources and costs**	14	Describe how costs were valued.	See the 'Measuring costs' section of the Methods and Supplemental Table S1 for unit costs
**Currency, price date, and conversion**	15	Report the dates of the estimated resource quantities and unit costs, plus the currency and year of conversion.	The dates of the estimated resource quantities are reported in the 'Participants' section of the Results section. Other items are reported in the 'Measuring costs' section of the same chapter
**Rationale and description of model**	16	If modelling is used, describe in detail and why used. Report if the model is publicly available and where it can be accessed.	Not applicable, a within trial cost effectiveness analysis was conducted
**Analytics and assumptions**	17	Describe any methods for analysing or statistically transforming data, any extrapolation methods, and approaches for validating any model used.	See the 'Incremental analyses' section of the Methods

Topic	No.	Item	Location where item is reported
**Characterising heterogeneity**	18	Describe any methods used for estimating how the results of the study vary for subgroups.	See the fifth sensitivity analysis (SA per protocol) in 'Appendix 3: further sensitivity analyses' of the Supplemental Material (Appendices)
**Characterising distributional effects**	19	Describe how impacts are distributed across different individuals or adjustments made to reflect priority populations.	Not reported (Not conducted) – the HEAP was developed before this updated CHEERS checklist was available
**Characterising uncertainty**	20	Describe methods to characterise any sources of uncertainty in the analysis.	See the 'Decision uncertainty' and 'Sensitivity analyses' section of the Methods
**Approach to engagement with patients and others affected by the study**	21	Describe any approaches to engage patients or service recipients, the general public, communities, or stakeholders (such as clinicians or payers) in the design of the study.	The patient self-report questionnaire was developed in consultation with non-trial patients. See the 'Measuring Costs' section of the Methods
**Results**			
**Study parameters**	22	Report all analytic inputs (such as values, ranges, references) including uncertainty or distributional assumptions.	Not applicable, a within trial cost effectiveness analysis was conducted
**Summary of main results**	23	Report the mean values for the main categories of costs and outcomes of interest and summarise them in the most appropriate overall measure.	See Tables 2 (Costs) and 3 (Outcomes), these are referred to in the 'Costs' and 'Outcomes' section of the Results.
**Effect of uncertainty**	24	Describe how uncertainty about analytic judgments, inputs, or projections affect findings. Report the effect of choice of discount rate and time horizon, if applicable.	Sensitivity analyses are reported in Table 5 and Supplemental Table A1. Within these Tables the estimated probability values for the cost-effectiveness acceptability curve (CEAC) are also reported for base-case and sensitivity analyses.
**Effect of engagement with patients and others affected by the study**	25	Report on any difference patient/service recipient, general public, community, or stakeholder involvement made to the approach or findings of the study	The patient self-report questionnaire was developed in consultation with non-trial patients. See the 'Measuring Costs' section of the Methods
**Discussion**			
**Study findings, limitations, generalisability, and current knowledge**	26	Report key findings, limitations, ethical or equity considerations not captured, and how these could affect patients, policy, or practice.	See the Discussion

For peer review only - http://bmjopen.bmj.com/site/about/guidelines.xhtml

Topic	No.	Item	Location where item is reported
**Other relevant information**			
**Source of funding**	27	Describe how the study was funded and any role of the funder in the identification, design, conduct, and reporting of the analysis	See the 'Conflicts of Interest and Source of Funding' section
**Conflicts of interest**	28	Report authors conflicts of interest according to journal or International Committee of Medical Journal Editors requirements.	See the 'Conflicts of Interest and Source of Funding' section

From: Husereau D, Drummond M, Augustovski F, et al. Consolidated Health Economic Evaluation Reporting Standards 2022 (CHEERS 2022) Explanation and Elaboration: A Report of the ISPOR CHEERS II Good Practices Task Force. Value Health 2022;25. doi:10.1016/j.jval.2021.10.008

For peer review only
